# Vaccine efficacy of NVX-CoV2373 against SARS-CoV-2 infection in adolescents in the USA: an ancillary study to a phase 3, observer-blinded, randomised, placebo-controlled trial

**DOI:** 10.1016/j.lanmic.2024.100984

**Published:** 2025-01-27

**Authors:** Meagan E Deming, Elizabeth R Brown, Monica A McArthur, Stephanie J Schrag, Melissa Arvay, Mike Humphrys, Jacques Ravel, Jeffrey Adelglass, Brandon Essink, David B Musante, Rebecca Maguire, Richard Gorman, Elizabeth Formentini, Robin Mason, Merlin L Robb, Kathleen M Neuzil, Rekha R Rapaka, Peter Wolff, Karen L Kotloff

**Affiliations:** Center for Vaccine Development and Global Health, University of Maryland School of Medicine, Baltimore, MD, USA; Fred Hutchinson Cancer Research Center, Seattle, WA, USA; Center for Vaccine Development and Global Health, University of Maryland School of Medicine, Baltimore, MD, USA; US COVID-19 Domestic Response and Division of Bacterial Diseases, National Center for Immunization and Respiratory Diseases, Centers for Disease Control and Prevention, Atlanta, GA, USA; US COVID-19 Domestic Response and Division of Bacterial Diseases, National Center for Immunization and Respiratory Diseases, Centers for Disease Control and Prevention, Atlanta, GA, USA; Institute for Genome Sciences, University of Maryland School of Medicine, Baltimore, MD, USA; Institute for Genome Sciences, University of Maryland School of Medicine, Baltimore, MD, USA; Research Your Health, Plano, TX, USA; Meridian Clinical Research, Omaha, NE, USA; M3-Emerging Medical Research, Durham, NC, USA; Center for Vaccine Development and Global Health, University of Maryland School of Medicine, Baltimore, MD, USA; Biomedical Advanced Research and Development Authority, Washington, DC, USA; Biomedical Advanced Research and Development Authority, Washington, DC, USA; Biomedical Advanced Research and Development Authority, Washington, DC, USA; Henry M Jackson Foundation for the Advancement of Military Medicine, Bethesda, MD, USA; Center for Vaccine Development and Global Health, University of Maryland School of Medicine, Baltimore, MD, USA; Center for Vaccine Development and Global Health, University of Maryland School of Medicine, Baltimore, MD, USA; National Institute of Allergy and Infectious Diseases, National Institutes of Health, Washington, DC, USA; Center for Vaccine Development and Global Health, University of Maryland School of Medicine, Baltimore, MD, USA

## Abstract

**Background:**

Although existing COVID-19 vaccines are known to be highly effective against severe disease and death, data are needed to assess their ability to reduce SARS-CoV-2 infection. We aimed to estimate the efficacy of the NVX-CoV2373 protein subunit vaccine against SARS-CoV-2 infection, regardless of symptoms, among adolescents.

**Methods:**

We performed an ancillary observational study (SNIFF) to the phase 3, observer-blinded, randomised, placebo-controlled PREVENT-19 trial that assessed vaccine efficacy against symptomatic COVID-19 in the USA. Participants in the PREVENT-19 trial included healthy adolescents aged 12–17 years and with no history of laboratory-confirmed SARS-CoV-2 infection. They were randomly assigned (2:1) to receive either the NVX-CoV2373 (Novavax, Gaithersburg, MD, USA) vaccine (immediate NVX-CoV2373 group) or placebo (delayed NVX-CoV2373 group) on days 0 and 21 (initial series). After 2 months, in a crossover series, participants received two doses, 21 days apart, of the intervention that they did not receive in their initial series. Participants at 47 of the PREVENT-19 sites were invited to participate in the SNIFF study and self-collect nasal swabs at home twice weekly for SARS-CoV-2 testing to assess vaccine efficacy against SARS-CoV-2 infection. This primary outcome was defined as the first identification of SARS-CoV-2 detected by RT-PCR, regardless of symptoms, with onset within 4 weeks after the second dose of the initial vaccination series until the second dose of the crossover series. Secondary outcomes were vaccine efficacy against asymptomatic and minimally symptomatic SARS-CoV-2 infection, durability of vaccine efficacy against SARS-CoV-2 infection, and durability of vaccine efficacy against asymptomatic and minimally symptomatic infections. Outcomes were analysed in the modified intention-to-treat population, which included all participants without previous SARS-CoV-2 infection and was restricted to participants enrolled within 4 weeks of the second dose of the primary (primary analysis population) or crossover (post-crossover analysis population) series. This study is registered with ClinicalTrials.gov (NCT04611802).

**Findings:**

Between June 1 and Dec 17, 2021, 1196 (53·2%) of the 2247 adolescent participants recruited in the PREVENT-19 trial enrolled in the SNIFF study. The primary analysis population included 471 participants in the immediate NVX-CoV2373 group and 220 in the delayed NVX-CoV2373 group. Incidence of SARS-CoV-2 infection was 14·9 cases per 100 person-years (95% CI 7·9–25·5) in the immediate group and 54·2 cases per 100 person-years (33·6–82·9) in the delayed group; vaccine efficacy was 73·5% (95% CI 47·1–86·7; p=0·0002). Incidence of minimally symptomatic or asymptomatic SARS-CoV-2 infection was 10·3 cases per 100 person-years (95% CI 4·7–19·6) in the immediate group and 36·1 cases per 100 person-years (19·8–60·7) in the delayed group; vaccine efficacy was 72·8% (95% CI 37·1–88·2; p=0·0023). After the second crossover dose, incidence of SARS-CoV-2 was 14·6 cases per 100 person-years (95% CI 8·6–23·0) in the immediate group (receiving placebo at crossover) and 9·1 cases per 100 person-years (3·0–21·3) in the delayed group, with a durability ratio of 160·3 (95% CI 59·5–431·6; p=0·35). Almost all infections after crossover were minimally symptomatic or asymptomatic, with a durability ratio of 151·4 (55·9–410·4; p=0·41).

**Interpretation:**

Among adolescents participating in the PREVENT-19 trial during the delta (B.1.617.2) variant wave of the COVID-19 pandemic, the NVX-CoV2373 vaccine was highly efficacious against SARS-CoV-2 infection regardless of symptoms, indicating its potential to reduce the reservoir of infections that contribute to community transmission.

**Funding:**

US Department of Health and Human Services, Administration for Strategic Preparedness and Response, Biomedical Advanced Research and Development Authority, National Institute of Allergy and Infectious Diseases, and National Institutes of Health.

## Introduction

The efficacy of SARS-CoV-2 vaccines in preventing clinically significant disease has been shown in phase 3, randomised, placebo-controlled trials and real-life effectiveness studies.^1–5^ Vaccination might also prevent infection; however, rigorous studies verifying this effect are scarce. Understanding the impact of vaccination on individuals with asymptomatic and minimally symptomatic infection is particularly important, given that unrecognised infections contribute to transmission events.^6–9^ Therefore, children and adolescents, in whom mild or asymptomatic SARS-CoV-2 infections are common, are especially relevant for understanding the impact of vaccination on onward transmission.^10^ Reducing infection as a strategy to reduce transmission has been identified as a priority for the next generation of COVID-19 vaccines.^11^ Improved understanding of vaccine-induced sterile immunity will help to inform these efforts.

NVX-CoV2373 (Novavax, Gaithersburg, MD, USA) is a SARS-CoV-2 recombinant spike protein vaccine co-formulated with the Matrix-M adjuvant (Novavax, Gaithersburg, MD, USA) and administered in two doses 21 days apart. This vaccine was reported to be safe and efficacious in a phase 3, randomised, observer-blinded, placebo-controlled trial (PREVENT-19) among adults in the USA and Mexico.^5^ Thereafter, a protocol expansion to include adolescents (aged 12–17 years) was initiated to assess vaccine efficacy against PCR-confirmed symptomatic COVID-19.^12^ We aimed to evaluate the efficacy of NVX-CoV2373 in preventing all SARS-CoV-2 infections, including those with minimal or no symptoms, among adolescents participating in the PREVENT-19 trial.

## Methods

### Study design and participants

We performed an ancillary observational study (SNIFF) to the PREVENT-19 paediatric expansion trial in the USA (appendix p 12). Briefly, the PREVENT-19 trial enrolled healthy adolescents aged 12–17 years with no history of laboratory-confirmed COVID-19 or SARS-CoV-2 infection between April 26, 2021, and June 5, 2021, from the USA and Mexico.^12^ Participants were randomly assigned to receive either the NVX-CoV2373 vaccine or placebo on days 0 and 21 (initial series).^12^ After 2 months (median 71 days [IQR 65–77]), a masked crossover was initiated on Aug 6, 2021, and completed on Oct 28, 2021 (crossover series), whereby participants then received two doses, 21 days apart, of the intervention that they did not receive in their initial series.^12^ Henceforth, we refer to participants who received the NVX-CoV2373 vaccine in the initial series (and placebo in the crossover series) as the immediate NVX-CoV2373 group, and to participants who received placebo in the initial series (and the vaccine in the crossover series) as the delayed NVX-CoV2373 group. Throughout the scheduled 24-month follow-up period, prespecified symptoms of COVID-19 were actively reported by participants’ parents or captured during weekly scheduled calls.

Adolescents participating in the PREVENT-19 trial belonging to one of 47 clinical sites participating in the SNIFF study across the USA were eligible to enrol in the SNIFF study at any time following the second dose of the initial series (appendix p 12). The study was planned to end when the second crossover injection was administered, but was extended for 3 additional months to further evaluate the duration of protection.

The SNIFF study protocol was approved by an independent central institutional review board, Western IRB–Copernicus Group (IRB00000533). Written informed consent was obtained from parents and assent from adolescent participants before any study procedures. This activity was reviewed by the US Centers for Disease Control and Prevention (CDC) and adhered to the applicable Code of Federal Regulations and CDC policy.

### Randomisation and masking

Participants in the PREVENT-19 trial were randomly assigned (2:1) without age stratification to receive two doses of NVX-CoV2373 or saline placebo on days 0 and 21 by use of a web-based interactive system.^12^ As an observer-blinded study, study participants, study personnel responsible for evaluating endpoints, and laboratories involved in immunogenicity testing were masked to allocation. To maintain masking, the personnel managing the vaccine preparation and injections were aware of the randomisation assignment and had no other role in the trial.^5^

### Procedures

For the SNIFF study, after a single in-person study enrolment visit, all procedures were performed remotely (appendix pp 4–5). Twice a week, participants used sterile flocked swabs (COPAN Diagnostics, Murrieta, CA, USA) or HydraFlok swabs (Puritan Guilford, ME, USA) to swab both anterior nares, before placing each swab in C2.1 buffer (Qiagen, Hilden, Germany) and scanning the accompanying tube label for tracking purposes using a customised phone app (MG Scanner app [version 1.2]). Participants kept samples at room temperature and returned them weekly to the Institute of Genome Sciences (Baltimore, MD, USA) by pre-paid mailer.

Swabs were tested for SARS-CoV-2 by RT-PCR with a modified version of the CDC’s 2019 nCoV real-time RT-PCR assay with emergency use authorisation by use of three single-plexed reactions for SARS-CoV-2 genes *N1* and *N3*, and human RNase P.^13^ Samples with cycle threshold (Ct) values of less than 40 were scored as detected, samples with no detectable RNase P were rejected as invalid, and samples with both *N1* and *N3* genes detected were considered positive for SARS-CoV-2 (appendix pp 4–5). Samples with *N1* Ct values of less than 32 were prepared for whole-genome sequencing and mapped to the Wuhan reference genome (NC_045512.2). Nextclade and Pangolin were used to generate variant calls, and clade and lineage assignments. Viral load was established by digital PCR by use of an *N3* target. The Institute of Genome Sciences performed the RT-PCR and genomic assays for the analyses in the SNIFF study; results from the PREVENT-19 trial were generated by a separate core laboratory.^12^ RT-PCR results from the SNIFF study were not shared with participants. On the rare occasion that participants with infection detected by RT-PCR neglected to scan their swabs appropriately, an adjudication committee imputed the probable timepoint of specimen collection (appendix p 5).

Aside from adverse events occurring within 15 min of swab collection, no clinical participant data were collected specifically for the SNIFF study. Instead, participant numbers were linked with data from the dataset of the PREVENT-19 trial to collect data (eg, study product assignment, daily symptomatology, and a derived variable classifying illnesses as symptomatic COVID-19) to inform the inclusion criteria for analysis populations, endpoint definitions, and summaries of participant characteristics. All cases of symptomatic COVID-19 in the PREVENT-19 trial were mild, defined as either fever, cough, or at least two other COVID-19 symptoms: dyspnoea, fatigue, myalgias, headache, anosmia, congestion, pharyngitis, rhinorrhoea, nausea, vomiting, or diarrhoea.^12^

### Outcomes

The primary outcome was the vaccine efficacy of NVX-CoV2373 against SARS-CoV-2 infection, defined as the first identification of SARS-CoV-2 detected by RT-PCR on a self-collected anterior nasal swab, regardless of symptoms, during follow-up accrued through the last negative test at or before the second dose of the crossover series, 4 weeks after the first dose of the crossover series, withdrawal from the study, or receipt of a SARS-CoV-2 vaccine outside of the trial, whichever occurred first. We included the three following secondary outcomes: vaccine efficacy against asymptomatic and minimally symptomatic SARS-CoV-2 infection, defined as SARS-CoV-2 infection not meeting the endpoints defined in the PREVENT-19 trial for symptomatic COVID-19; the durability of vaccine efficacy against SARS-CoV-2 infection; and the durability of vaccine efficacy against minimally or asymptomatic infections in the post-crossover analysis population. All three secondary outcomes were measured during follow-up as defined for the primary outcome.

As exploratory outcomes, we sought to estimate the viral load of SARS-CoV-2 as a proxy of transmission and the SARS-CoV-2 genomes were sequenced to identify variants (appendix pp 4–5). The publication of additional exploratory outcomes—namely, estimating vaccine efficacy on duration of SARS-CoV-2 infection and vaccine efficacy against a composite outcome of asymptomatic and presymptomatic SARS-CoV-2 infection—has been deferred pending available resources.

### Statistical analysis

This ancillary study was designed to enrol 2000 adolescents to detect a vaccine efficacy of at least 60% with an assumed attack rate in unvaccinated participants of 5·7 cases per 100 000 person-weeks (0·3 cases per 100 person-years) over 6 weeks of follow-up, with 80% power and a two-sided alpha level of 0·05 against a null hypothesis of vaccine efficacy equating 0. Vaccine efficacy was estimated as 1–HR, where HR is the hazard ratio, from a Cox proportional hazards model comparing randomisation with immediate versus delayed vaccination.

The intention-to-treat population included participants who submitted at least one swab. The SNIFF study protocol aimed to enrol participants without SARS-CoV-2 infection at the time of their second dose of the initial (masked) vaccination series, yielding a modified intention-to-treat population comprising participants who had at least one valid RT-PCR result while enrolled in the SNIFF study, but excluding those with known SARS-CoV-2 infection identified by RT-PCR or detection of anti-nucleocapsid antibodies on day 0. However, many participants enrolled after the second dose of the initial series, precluding the ability to establish if they had acquired infection between the second dose and the first swab. To account for this potentially confounding factor, we created a primary analysis population limited to participants in the modified intention-to-treat analysis who had enrolled within 4 weeks of receiving the second dose of the initial vaccination series. All primary analyses were performed on this population.

The post-crossover analysis population included the subset of participants in the modified intention-to-treat analysis who had enrolled within 4 weeks of receiving the second dose of the crossover series (ie, dose 4) without evidence of previous infection by the second dose of the crossover series. Vaccine efficacy is summarised for the post-crossover analysis population by the durability ratio, a ratio of COVID-19 incidence in the immediate NVX-CoV2373 group to incidence in the delayed NVX-CoV2373 group. The durability ratio was estimated by the HR from a Cox proportional hazards model based on follow-up time of the primary endpoint from the second dose of the crossover series to withdrawal from the study, receipt of a SARS-CoV-2 vaccine outside of the trial, or end of the study, whichever occurred first.

The sensitivity analyses of vaccine efficacy were conducted on the primary analysis population and the modified intention-to-treat population by use of a Cox proportional hazards model fit on a calendar time scale. These analyses aimed to characterise any differences in vaccine efficacy measurements when the period of observation for the primary analysis population and the modified intention-to-treat population was terminated at the first crossover dose compared with when the first crossover dose was included (primary outcome).

To estimate the exploratory objectives of SARS-CoV-2 viral load as a proxy of transmission and the sequences of infection, peak viral load was analysed in all participants with infection in the modified intention-to-treat population by use of a linear regression model comparing one and two doses with no dose, adjusted for age and sex. An additional model was fit comparing either one or two doses with no dose.

All statistical analyses were performed with R (version 4.0.4) and SAS (version 9.4). This study is registered with ClinicalTrials.gov (NCT04611802).

### Role of the funding source

The Biomedical Advanced Research and Development Authority (BARDA) and Health and Human Services representatives participated in the study design, regulatory and administrative processes of the study, and reviewed and approved the final version of the manuscript. The funders had no role in data collection, data analysis, data interpretation, or writing of the report.

## Results

**Figure 1:**
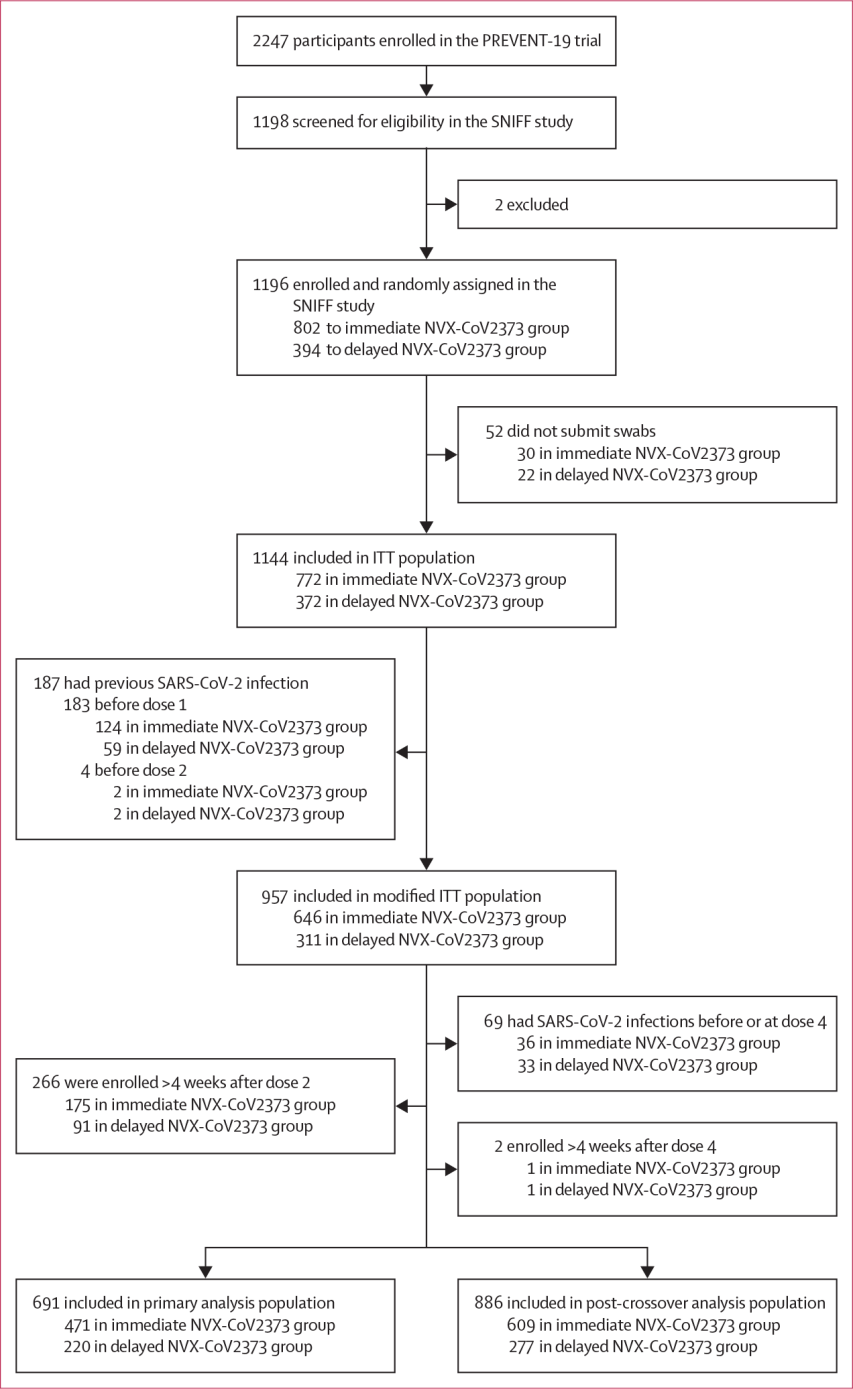
SNIFF study profile The ITT population included participants who submitted at least one swab, and the modified ITT population included those without evidence of SARS-CoV-2 infection before the second dose of the primary series. The primary analysis population included participants in the modified ITT population enrolled within 4 weeks of receiving the second dose of the initial vaccination series. The post-crossover analysis population included the subset of participants in the modified ITT population who had enrolled within 4 weeks of receiving the second dose of the crossover series (ie, dose 4) without evidence of previous infection by this second dose. ITT=intention-to-treat.

Between June 1 and Dec 17, 2021, among 2247 adolescent participants recruited in the PREVENT-19 trial, 1198 (53·3%) adolescents were screened and 1196 (53·2%) were enrolled in the SNIFF study (figure 1; appendix p 12). 52 participants who never submitted a swab were excluded to form the intention-to-treat population comprising 1144 participants. An additional 187 participants with previous SARS-CoV-2 infection were excluded, leaving 957 participants in the modified intention-to-treat population: 646 (67·5%) in the immediate NVX-CoV2373 group (receiving the vaccine in the initial series and placebo in the crossover series) and 311 (32·5%) in the delayed NVX-CoV2373 group (receiving placebo in the initial series and the vaccine in the crossover series). Among participants in the modified intention-to-treat population, 28 489 swabs were submitted within 45 252 windows of expected swab collection, yielding a 62·9% adherence with no significant difference between groups in either the number of swabs collected or participant-level compliance to swab collection windows (appendix p 9). The primary analysis population included 691 participants (471 [68·3%] in the immediate NVX-CoV2373 group and 220 [31·8%] in the delayed NVX-CoV2373 group) who enrolled within 4 weeks of receiving the second dose. The post-crossover analysis population included 886 participants without infection before the second dose of over the crossover series, of whom 609 (68·7%) had received the vaccine in the initial series (immediate NVX-CoV2373 group) and 277 (31·3%) had received the vaccine in the crossover series (delayed NVX-CoV2373 group). Demographics were well balanced between intervention groups (table 1; appendix pp 7–8). No adverse events from swabbing were reported.

**Figure 2:**
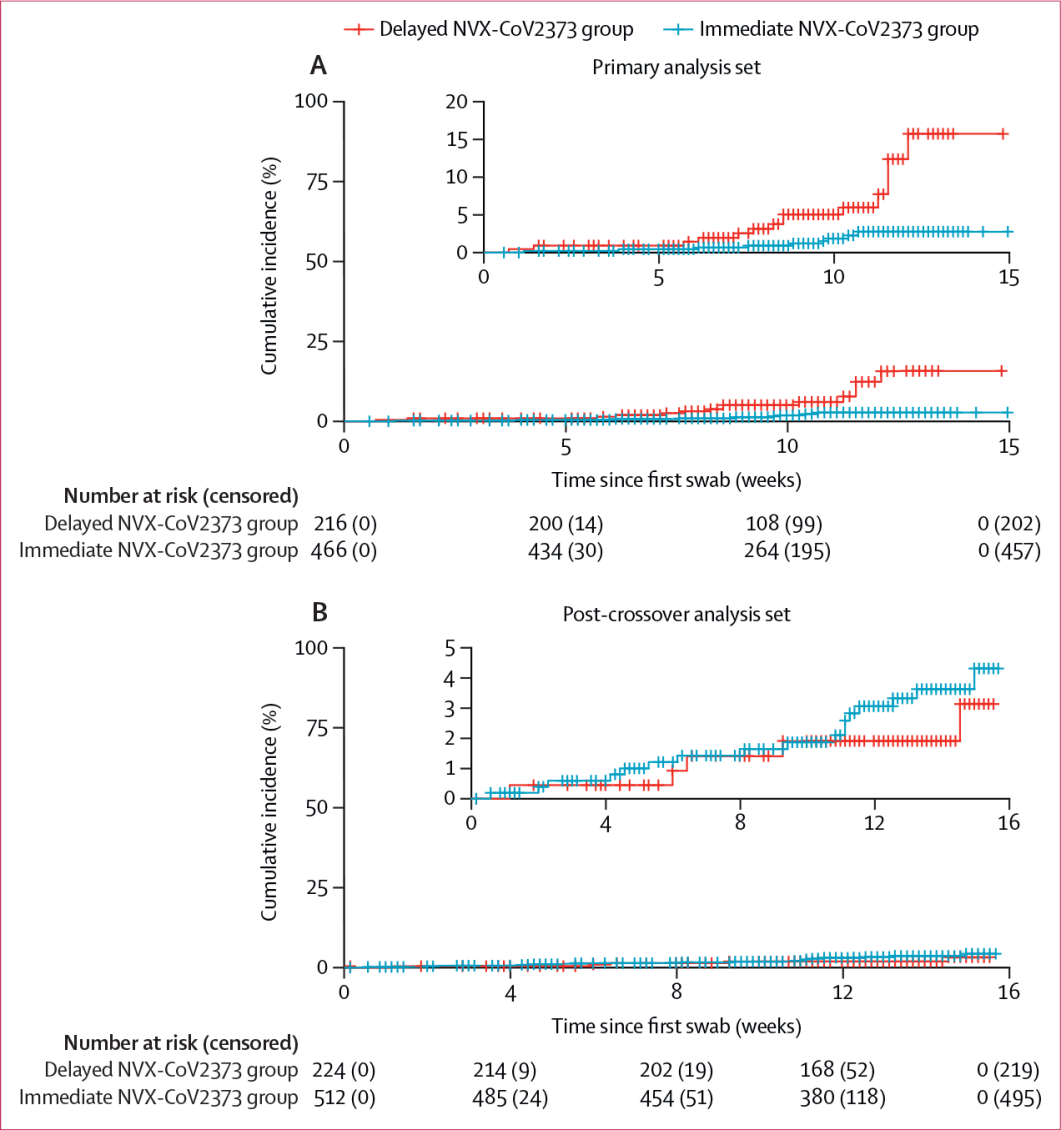
Incidence of SARS-CoV-2 infection by intervention group The cumulative incidence of any SARS-CoV-2 infection in the immediate NVX-CoV2373 group (receiving the vaccine in the initial series and placebo in the crossover series) and delayed NVX-CoV2373 group (receiving placebo in the initial series and the vaccine in the crossover series) in the primary analysis population (A) and post-crossover analysis population (B). Incidence was estimated by Kaplan–Meir curves based on follow-up time from the primary analysis. Insets show data on an enlarged y axis.

In the primary analysis population, SARS-CoV-2 was detected in 21 (9·5%) participants in the delayed NVX-CoV2373 group, with an incidence of 54·2 cases per 100 person-years (95% CI 33·6–82·9), and 13 (2·8%) in the immediate NVX-CoV2373 group, with an incidence of 14·9 cases per 100 person-years (7·9–25·5). The resultant vaccine efficacy was 73·5% (95% CI 47·1–86·7; p=0·0002; figure 2). Infections during the crossover period (Aug 6–Oct 28, 2021) increased substantially in line with the surge in delta (B.1.617.2) variant infections in children across the USA (figure 2; appendix p 11). The sensitivity analysis that was conducted to evaluate the impact of the first dose of vaccine received at crossover in the primary analysis population measured a vaccine efficacy of 77·8% (95% CI 35·0–92·4; p=0·0060; appendix p 8).

Minimally symptomatic or asymptomatic infections not meeting the endpoint of symptomatic COVID-19 in the PREVENT-19 trial were identified in 14 (66·6%) of 21 participants with SARS-CoV-2 infection in the delayed NVX-CoV2373 group, and in nine (69·2%) of 13 participants in the immediate NVX-CoV2373 group (table 2). The resultant incidence of minimally symptomatic or asymptomatic infections was 10·3 cases per 100 person-years (95% CI 4·7–19·6) in the immediate NVX-CoV2373 group, compared with 36·1 cases per 100 person-years (19·8–60·7) in the delayed NVX-CoV2373 group. Vaccine efficacy for minimally symptomatic or asymptomatic cases was 72·8% (95% CI 37·1–88·2; p=0·0023; table 2; figure 2B).

After the second crossover dose, the immediate NVX-CoV2373 group (receiving placebo at crossover) had 14·6 infections per 100 person-years (95% CI 8·6–23·0; table 3). Incidence of SARS-CoV-2 after crossover was lower in the delayed NVX-CoV2373 group at 9·1 cases per 100 person-years (3·0–21·3), resulting in a durability ratio of 160·3 (95% CI 59·5–431·6; p=0·35; figure 2B). Almost all infections in the post-crossover analysis population were minimally symptomatic orasymptomatic, with a similar non-significant durability ratio of 151·4 (55·9–410·4; p=0·41).

Of the 38 461 total swabs received by Institute of Genome Sciences, 36 428 (94·7%) had been registered by the participant in the MG Scanner app and 2033 (5·3%) were unmatched, with no registration event. Of these swabs, 247 (0·7%) of the 36 428 matched and 289 (14·2%) of the unmatched samples were rejected for undetectable RNase P transcripts. Including duplicates and unmatched swabs, RT-PCR results were obtained from 37 925 (98·6%) samples. Detectable SARS-CoV-2 RNA was identified in swabs from 71 participants, with viral loads available for 69 participants. Compared with no receipt of vaccine, SARS-CoV-2 infection following two doses of vaccine was associated with an age-adjusted and sex-adjusted decrease in peak viral load of 0·72 log_10_ copies per swab (95% CI –0·02 to 1·46; p=0·066). Of the 47 samples testing positive for SARS-CoV-2 with cycle thresholds below 34, three (6·4%) samples were identified as omicron (B.1.1.529) variants, 39 (83·0%) as delta variants, and five were uncategorised (poor quality reads).

## Discussion

By leveraging an ongoing phase 3 trial evaluating the vaccine efficacy of NVX-CoV2373 in preventing COVID-19, we were able to use a randomised, observer-blinded, placebo-controlled methodology to measure the ability of NVX-CoV2373 to prevent RT-PCR-confirmed SARS-CoV-2 infection among adolescents.^12^ The observed vaccine efficacy of 73·5% for the prevention of any SARS-CoV-2 infection and 72·8% for minimally symptomatic or asymptomatic infection (not meeting the COVID-19 case definition) during the delta variant wave in the current study are similar to the vaccine efficacy of 79·5% (95% CI 46·8–92·1) measured contemporaneously against PCR-confirmed symptomatic COVID-19 in the same study population as part of the PREVENT-19 trial. The relative efficacy of immediate versus delayed vaccination showed no significant difference in the incidence of SARS-CoV-2 over the follow-up period of the study. These findings expand the benefits of NVX-CoV2373 to include the induction of sterilising immunity in the upper airway, and suggest that parenteral administration of a SARS-CoV-2 protein vaccine has the potential to significantly reduce the reservoir of subclinical infections that contribute to community transmission.

Few studies have systematically characterised vaccine efficacy against SARS-CoV-2 infection with specific attention to asymptomatic infection. Variations in methodology, circulating variants, and study populations have contributed to the variable results that have been observed to date.^14^ Several phase 3 trials have assessed vaccine efficacy against asymptomatic infection by performing PCR on nasal swabs, anti-nucleocapsid seroconversion, or both at prespecified study visits. The phase 3 trial exploring the safety and efficacy of NVX-CoV2373 among adults reported a vaccine efficacy of 76·3% (95% CI 57·4 to 86·8) against asymptomatic infection, as measured by anti-nucleocapsid seroconversion, which corroborates our findings.^15^ A trial of mRNA-1273 in the USA during the alpha (B.1.1.7) variant wave measured a lower estimate of vaccine efficacy against asymptomatic infection in adolescents (39·2% [95% CI –24·7 to 69·7]) compared with adults (63·0% [56·6 to 68·5]), albeit with confidence limits that were wide and overlapping.^16,17^ Factors contributing to this age discrepancy probably included infrequent swab collection, potentially missing infections of shorter duration, and use of anti-nucleocapsid antibody seroconversion as a diagnostic criterion for infection, which is less reliable in vaccinated individuals than unvaccinated individuals.^18^ Other phase 3 trials have investigated the vaccine efficacy of adenovirus vector vaccines in adults during the circulation of multiple variants in the pre-delta era, reporting a range between 41·7% (95% CI 36·3 to 46·7) for the AD26.COV2.S vaccine (Janssen, Beerse, Belgium) and 64·3% (95% CI 56·1 to 71·0) for the AZD1222 (ChAdOx1 nCoV-19) vaccine (AstraZeneca, Cambridge, UK) against combined asymptomatic and symptomatic infection, as measured by anti-nucleocapsid antibodies.^19,20^ The efficacy of Ad26.COV2.S specifically against asymptomatic infection was 28·9% (20·0 to 36·8).^20^ During the delta variant wave, the BNT162b2 mRNA vaccine (Pfizer-BioNTech, Mainz, Germany) showed an efficacy against the outcome of combined symptomatic and asymptomatic infection of 87% (49 to 97) among a cohort of adolescents who submitted weekly symptom reports and nasal swabs for RT-PCR.^21^ Efficacy data in the context of omicron variants remain sparse. Several cohort studies that controlled for time since vaccination (to differentiate the effects of immune escape from divergent variants due to waning immunity) suggest that mRNA vaccines are less effective against omicron variants than pre-omicron variants.^21,22^ Before the current study, the efficacy and durability of well matched recombinant protein booster vaccines in preventing SARS-CoV-2 infection had not been established.

Our study shows the feasibility of conducting a streamlined ancillary study concurrent with a pivotal COVID-19 efficacy trial. The remote procedures permitted frequent contact with participants, averted supply shortfalls, encouraged adherence to sample collection, and allowed for the tracking of samples without interfering with the conduct of the PREVENT-19 trial. Our findings suggest these remote swabbing studies are feasible and can provide valuable information about the ability of vaccines to confer sterile immunity.

The onward transmission of SARS-CoV-2 seems to be lower from fully vaccinated individuals than from unvaccinated individuals,^23^ although the reported confidence intervals are wide and overlapping.^24,25^ Mean peak viral load, a possible proxy for infectivity, was found to be similar between vaccinated and unvaccinated individuals and between those with and without symptoms during the delta and omicron variant waves.^25–28^ The non-significant reduction in peak viral load among fully vaccinated participants in our study extends these observations to recipients of a recombinant protein COVID-19 vaccine.

A strength of the SNIFF study is the observer-blind, randomised, placebo-controlled design that more reliably estimates vaccine efficacy by minimising confounding due to unmeasured differences between vaccinated and unvaccinated participants that might be seen with cohort and retrospective observational studies. Other strengths include the exclusion of participants with previous SARS-CoV-2 infection from the analysis, as well as frequent swab collection and symptom reporting to mitigate the risk of misclassifying symptomatic and asymptomatic infections. Infection was diagnosed prospectively by PCR in systematically collected swabs. Our study population was geographically and demographically diverse. Because of the unexpectedly high incidence of SARS-CoV-2, we accrued sufficient statistical power to assess the primary and secondary outcomes despite the initial sample size being smaller than expected. To our knowledge, this is the first study to assess the efficacy of a recombinant protein vaccine against SARS-CoV-2 infection.

The current study has several limitations. First, the incidence of SARS-CoV-2 infection could have been overestimated if participants were more likely to swab when even minimal symptoms were present; however, symptomatology was equally distributed between intervention groups and estimates of vaccine efficacy were not likely affected. Second, weekly shipping of nasal swab specimens at room temperature could have potentially resulted in false negative results. However, the stability of detecting SARS-CoV-2 RNA has been shown previously for 14 days at room temperature;^29^ the Institute of Genome Sciences assay was validated for 10 days; and human RNase P amplification was required for swab results to be considered valid (appendix pp 4–5). Third, this study was not powered for a durability analysis; therefore, despite numerical differences in incidence rates after crossover, we could not evaluate with precision the extended trajectory of vaccine efficacy. The post-crossover analysis is further limited without including a placebo group. Nearly all infections in this group were reported as minimally symptomatic or asymptomatic, suggesting that participants under-reported their symptoms due to participant bias or study fatigue. Finally, the SNIFF study was conducted during the delta variant wave and might have limited generalisability to more divergent omicron variants.^21^

High vaccine efficacy in preventing infection can efficiently prevent the onward transmission of SARS-CoV-2. Increasing evidence emphasises the importance of children and adolescents in transmitting SARS-CoV-2.^30^ This age group in particular have asymptomatic or mild infections, and detection of SARS-CoV-2 by PCR frequently precedes symptoms.^31^ Secondary attack rates are higher among individuals exposed to unvaccinated children and adolescents than vaccinated individuals in this age group.^31,32^ A population-based study in Germany attributed up to 20% of infections to schoolchildren, the delta variant outbreak in Israel was traced to unvaccinated adolescents, and a study of case-ascertained household transmission found that 10% of exposed household contacts developed symptomatic COVID-19 after exposure to asymptomatic children.^33–35^ Despite high seroprevalence rates among children and adolescents, as of May, 2024, only 14% of this age group had received the updated 2023–24 COVID-19 vaccine in the USA.^36^

We found that the vaccine efficacy of NVX-CoV2373 against SARS-CoV-2 infection among adolescents across the USA was 73·5% against all infections and 72·8% against asymptomatic or minimally symptomatic infections. With only a third of SARS-CoV-2 infections in the SNIFF study meeting symptomatic criteria, these data will be informative in assessing the risks and benefits of booster vaccination schedules in adolescents.

## Supplementary Material

MMC1

## Figures and Tables

**Table 1:** Participant characteristics in the modified intention-to-treat population

	Immediate NVX-CoV2373 group (n=646)	Delayed NVX-CoV2373 group (n=311)	Total (n=957)

**Age, years**			
Mean (SD)	14·0 (1·4)	13·9 (1·4)	14·0 (1·4)
**Age group, years**			
12 to <15	413 (63·9%)	200 (64·3%)	613 (64·1%)
15 to <18	233 (36·1%)	111 (35·7%)	344 (35·9%)
**Sex at birth**			
Male	334 (51·7%)	162 (52·1%)	496 (51·8%)
Female	312 (48·3%)	149 (47·9%)	461 (48·2%)
**Latinx or Hispanic ethnicity**			
Yes	114 (17·6%)	59 (19·0%)	173 (18·1%)
No	530 (82·0%)	252 (81·0%)	782 (81·7%)
**Race**			
White	496 (76·8%)	227 (73·0%)	723 (75·5%)
Black or African American	77 (11·9%)	40 (12·9%)	117 (12·2%)
American Indian or Alaska Native	8 (1·2%)	5 (1·6%)	13 (1·4%)
Native Hawaiian or other Pacific Islander	1 (0·2%)	1 (0·3%)	2 (0·2%)
Asian	22 (3·4%)	15 (4·8%)	37 (3·9%)
Mixed origin	39 (6·0%)	21 (6·8%)	60 (6·3%)
Not reported	3 (0·5%)	2 (0·6%)	5 (0·5%)
**Height, cm**			
Mean (SD; range)	164·9 (10·3; 99–196)	163·9 (10·3; 124–193)	164·5 (10·3; 99–196)
**Weight, kg**			
Mean (SD; range)	66·5 (21·1; 32–154)	62·8 (19·0; 26–174)	65·3 (20·5; 26–174)
**BMI, kg/cm^2^**			
Mean (SD; range)	24·3 (6·8; 14–53)	23·3 (6·4; 10–64)	24·0 (6·7; 10–64)
**Student attending school in person**			
Yes	424 (65·6%)	205 (65·9%)	629 (65·7%)
No	222 (34·4%)	106 (34·1%)	328 (34·3%)

Data are n (%), unless otherwise indicated.

**Table 2:** NVX-CoV2373 vaccine efficacy against SARS-CoV-2 infection by symptom status in the primary analysis set

	Participants	Events	Person-years	Incidence per 100 person-years (95% CI)	Vaccine efficacy (95% CI)	p value

**All infections**						
Immediate NVX-CoV2373 group	466	13	87·2	14·9 (7·9–25·5)	73·5% (47·1–86·7)	0·0002
Delayed NVX-CoV2373 group	216	21	38·7	54·2 (33·6–82·9)	··	··
**Minimally symptomatic or asymptomatic infections**						
Immediate NVX-CoV2373 group	466	9	87·2	10·3 (4·7–19·6)	72·8% (37·1–88·2)	0·0023
Delayed NVX-CoV2373 group	216	14	38·7	36·1 (19·8–60·7)	··	··

**Table 3:** NVX-CoV2373 vaccine durability by symptom status in the post-crossover analysis set

	Participants	Events	Person-years	Incidence per 100 person-years (95% CI)	Durability ratio (95% CI)	p value

**All infections**						
Immediate NVX-CoV2373 group	512	18	123·5	14·6 (8·6–23·0)	160·3 (59·5–431·6)	0·35
Delayed NVX-CoV2373 group	224	5	54·9	9·1 (3·0–21·3)	··	··
**Minimally symptomatic or asymptomatic infections**						
Immediate NVX-CoV2373 group	512	17	123·5	13·8 (8·0–22·0)	151·4 (55·9–410·4)	0·41
Delayed NVX-CoV2373 group	224	5	54·9	9·1 (3·0–21·3)	··	··

## Data Availability

Participant data will not be available from this study because these data remain linked to the results of the PREVENT-19 trial.^5^ The study protocol, statistical analysis plan, informed consent form, and study materials are available in the appendix (pp 27–197).

## Research in context

### Evidence before this study

To contextualise the results of this study, we sought the available literature for studies evaluating the ability of COVID-19 vaccines to prevent infection or reduce transmission. We searched PubMed for studies published in English from database inception to June 1, 2021, using the following search terms: (“COVID-19” OR “SARS-CoV-2”) AND “vaccine” AND (“Novavax” OR “subunit protein”). This search showed that, although early-phase clinical trials of subunit protein vaccines were shown to prevent clinically significant infection, rigorous studies evaluating the ability of NVX-CoV2373 (Novavax Gaithersburg, MD, USA) and other subunit SARS-CoV-2 spike protein vaccines to protect against asymptomatic and minimally symptomatic SARS-CoV-2 infection and transmission in humans were scarce. An updated search to include all publications up to July 12, 2024, identified the results of a completed phase 3 trial, reporting a vaccine efficacy of NVX-CoV2373 of 76·3% against asymptomatic infections measured by nucleocapsid seroconversion among adults. Studies of other vaccine candidates on mRNA or viral vectored platforms showed a lower vaccine efficacy against asymptomatic infections than symptomatic SARS-CoV-2 infections. Another study of the mRNA-1273 vaccine in the USA during the alpha (B.1.1.7) variant wave showed a lower and non-significant vaccine efficacy against asymptomatic infection in adolescents, despite showing high vaccine efficacy against infection for adults.

### Added value of this study

This study was designed to assess whether a subunit protein vaccine could prevent any SARS-CoV-2 infection, including asymptomatic and minimally symptomatic infection (ie, not meeting symptom definitions in the primary PREVENT-19 trial), or reduce viral load in an adolescent study population. During a large, randomised, placebo-controlled trial (PREVENT-19), we conducted a remote ancillary study with self-swabbing of the anterior nares during the delta (B.1.617.2) variant wave and showed a high vaccine efficacy against all infections among adolescents, regardless of symptomatology. Two-thirds of detected infections were asymptomatic or minimally symptomatic, with a similar vaccine efficacy to that seen for symptomatic SARS-CoV-2 infections in the PREVENT-19 trial. Vaccinated participants with infection had SARS-CoV-2 RNA concentrations that were not significantly different to unvaccinated participants with infection.

### Implications of all the available evidence

This ancillary study appended to a double-blind, placebo-controlled, phase 3 clinical trial designed to assess efficacy against symptomatic COVID-19 showed that the NVX-CoV2373 vaccine was highly efficacious in preventing all SARS-CoV-2 infection among adolescents. The degree to which a SARS-CoV-2 vaccine can prevent infection (and by extension, onward transmission) can inform both individual vaccination decisions and vaccine policy when considering potential risks of transmission, long COVID-19 syndrome, and benefit–cost analyses for vaccination strategies among children and adolescents.

## References

[1] Olson SM, Newhams MM, Halasa NB, Effectiveness of BNT162b2 vaccine against critical COVID-19 in adolescents. N Engl J Med 2022; 386: 713–23. 35021004 10.1056/NEJMoa2117995 PMC8781318

[2] Feikin DR, Higdon MM, Abu-Raddad LJ, Duration of effectiveness of vaccines against SARS-CoV-2 infection and COVID-19 disease: results of a systematic review and meta-regression. Lancet 2022; 399: 924–44. 35202601 10.1016/S0140-6736(22)00152-0 PMC8863502

[3] Polack FP, Thomas SJ, Kitchin N, Safety and efficacy of the BNT162b2 mRNA COVID-19 vaccine. N Engl J Med 2020; 383: 2603–15. 33301246 10.1056/NEJMoa2034577 PMC7745181

[4] Baden LR, El Sahly HM, Essink B, Efficacy and safety of the mRNA-1273 SARS-CoV-2 vaccine. N Engl J Med 2021; 384: 403–16. 33378609 10.1056/NEJMoa2035389 PMC7787219

[5] Dunkle LM, Kotloff KL, Gay CL, Efficacy and safety of NVX-CoV2373 in adults in the United States and Mexico. N Engl J Med 2022; 386: 531–43. 34910859 10.1056/NEJMoa2116185 PMC8693692

[6] Cohen C, Kleynhans J, von Gottberg A, SARS-CoV-2 incidence, transmission, and reinfection in a rural and an urban setting: results of the PHIRST-C cohort study, South Africa, 2020–21. Lancet Infect Dis 2022; 22: 821–34. 35298900 10.1016/S1473-3099(22)00069-X PMC8920516

[7] Johansson MA, Quandelacy TM, Kada S, SARS-CoV-2 transmission from people without COVID-19 symptoms. JAMA Netw Open 2021; 4: e2035057. 33410879 10.1001/jamanetworkopen.2020.35057 PMC7791354

[8] Thompson MG, Burgess JL, Naleway AL, Prevention and attenuation of COVID-19 with the BNT162b2 and mRNA-1273 vaccines. N Engl J Med 2021; 385: 320–29. 34192428 10.1056/NEJMoa2107058 PMC8262622

[9] Diarra M, Ndiaye R, Barry A, Analysis of contact tracing data showed contribution of asymptomatic and non-severe infections to the maintenance of SARS-CoV-2 transmission in Senegal. Sci Rep 2023; 13: 9121. 37277417 10.1038/s41598-023-35622-6 PMC10240476

[10] Mehta NS, Mytton OT, Mullins EWS, SARS-CoV-2 (COVID-19): what do we know about children? A systematic review. Clin Infect Dis 2020; 71: 2469–79. 32392337 10.1093/cid/ciaa556 PMC7239259

[11] Administration for Strategic Preparedness and Response. Project NextGen: enhancing preparedness for future COVID-19 strains & variants with next generation medical countermeasures. 2023. https://aspr.hhs.gov/NextGen/Pages/Default.aspx (accessed Aug 26, 2023).

[12] Áñez G, Dunkle LM, Gay CL, Safety, immunogenicity, and efficacy of the NVX-CoV2373 COVID-19 vaccine in adolescents: a randomized clinical trial. JAMA Netw Open 2023; 6: e239135. 37099299 10.1001/jamanetworkopen.2023.9135 PMC10536880

[13] Lu X, Wang L, Sakthivel SK, US CDC real-time reverse transcription PCR panel for detection of severe acute respiratory syndrome coronavirus 2. Emerg Infect Dis 2020; 26: 1654–65. 32396505 10.3201/eid2608.201246 PMC7392423

[14] Ssentongo P, Ssentongo AE, Voleti N, SARS-CoV-2 vaccine effectiveness against infection, symptomatic and severe COVID-19: a systematic review and meta-analysis. BMC Infect Dis 2022; 22: 439. 35525973 10.1186/s12879-022-07418-y PMC9077344

[15] Heath PT, Galiza EP, Baxter DN, Safety and efficacy of the NVX-CoV2373 coronavirus disease 2019 vaccine at completion of the placebo-controlled phase of a randomized controlled trial. Clin Infect Dis 2023; 76: 398–407. 36210481 10.1093/cid/ciac803 PMC9619635

[16] Ali K, Berman G, Zhou H, Evaluation of mRNA-1273 SARS-CoV-2 vaccine in adolescents. N Engl J Med 2021; 385: 2241–51. 34379915 10.1056/NEJMoa2109522 PMC8385554

[17] El Sahly HM, Baden LR, Essink B, Efficacy of the mRNA-1273 SARS-CoV-2 vaccine at completion of blinded phase. N Engl J Med 2021; 385: 1774–85. 34551225 10.1056/NEJMoa2113017 PMC8482810

[18] Follmann D, Janes HE, Buhule OD, Antinucleocapsid antibodies after SARS-CoV-2 infection in the blinded phase of the randomized, placebo-controlled mRNA-1273 COVID-19 vaccine efficacy clinical trial. Ann Intern Med 2022; 175: 1258–65. 35785530 10.7326/M22-1300 PMC9258784

[19] Falsey AR, Sobieszczyk ME, Hirsch I, Phase 3 safety and efficacy of AZD1222 (ChAdOx1 nCoV-19) COVID-19 vaccine. N Engl J Med 2021; 385: 2348–60. 34587382 10.1056/NEJMoa2105290 PMC8522798

[20] Sadoff J, Gray G, Vandebosch A, Final analysis of efficacy and safety of single-dose Ad26.COV2.S. N Engl J Med 2022; 386: 847–60. 35139271 10.1056/NEJMoa2117608 PMC8849184

[21] Fowlkes AL, Yoon SK, Lutrick K, Effectiveness of 2-dose BNT162b2 (Pfizer BioNTech) mRNA vaccine in preventing SARS-CoV-2 infection among children aged 5–11 years and adolescents aged 12–15 years—PROTECT cohort, July 2021–February 2022. MMWR Morb Mortal Wkly Rep 2022; 71: 422–28. 35298453 10.15585/mmwr.mm7111e1 PMC8942308

[22] Gram MA, Emborg HD, Schelde AB, Vaccine effectiveness against SARS-CoV-2 infection or COVID-19 hospitalization with the alpha, delta, or omicron SARS-CoV-2 variant: a nationwide Danish cohort study. PLoS Med 2022; 19: e1003992. 36048766 10.1371/journal.pmed.1003992 PMC9436060

[23] Hsu L, Hurraß J, Kossow A, Breakthrough infections with the SARS-CoV-2 delta variant: vaccinations halved transmission risk. Public Health 2022; 204: 40–42. 35152039 10.1016/j.puhe.2022.01.005 PMC8747938

[24] Kim YC, Kim B, Son NH, Vaccine effect on household transmission of omicron and delta SARS-CoV-2 variants. J Korean Med Sci 2023; 38: e9. 36593690 10.3346/jkms.2023.38.e9 PMC9807772

[25] Singanayagam A, Hakki S, Dunning J, Community transmission and viral load kinetics of the SARS-CoV-2 delta (B.1.617.2) variant in vaccinated and unvaccinated individuals in the UK: a prospective, longitudinal, cohort study. Lancet Infect Dis 2022; 22: 183–95. 34756186 10.1016/S1473-3099(21)00648-4 PMC8554486

[26] Ke R, Martinez PP, Smith RL, Daily longitudinal sampling of SARS-CoV-2 infection reveals substantial heterogeneity in infectiousness. Nat Microbiol 2022; 7: 640–52. 35484231 10.1038/s41564-022-01105-z PMC9084242

[27] Kissler SM, Fauver JR, Mack C, Viral dynamics of SARS-CoV-2 variants in vaccinated and unvaccinated persons. N Engl J Med 2021; 385: 2489–91. 34941024 10.1056/NEJMc2102507 PMC8693673

[28] Riemersma KK, Haddock LA 3rd, Wilson NA, Shedding of infectious SARS-CoV-2 despite vaccination. PLoS Pathog 2022; 18: e1010876. 36178969 10.1371/journal.ppat.1010876 PMC9555632

[29] Bruce EA, Mills MG, Sampoleo R, Predicting infectivity: comparing four PCR-based assays to detect culturable SARS-CoV-2 in clinical samples. EMBO Mol Med 2022; 14: e15290. 34862752 10.15252/emmm.202115290 PMC8819313

[30] McLean HQ, Grijalva CG, Hanson KE, Household transmission and clinical features of SARS-CoV-2 infections. Pediatrics 2022; 149: e2021054178. 35194642 10.1542/peds.2021-054178 PMC9097956

[31] Chu VT, Yousaf AR, Chang K, Household transmission of SARS-CoV-2 from children and adolescents. N Engl J Med 2021; 385: 954–56. 34289272 10.1056/NEJMc2031915 PMC8314736

[32] Baker JM, Shah MM, O’Hegarty M, Primary and secondary attack rates by vaccination status after a SARS-CoV-2 B.1.617.2 (delta) variant outbreak at a youth summer camp—Texas, June 2021. J Pediatric Infect Dis Soc 2022; 11: 550–56. 36043454 10.1093/jpids/piac086 PMC9452135

[33] Heinsohn T, Lange B, Vanella P, Infection and transmission risks of COVID-19 in schools and their contribution to population infections in Germany: a retrospective observational study using nationwide and regional health and education agency notification data. PLoS Med 2022; 19: e1003913. 36538517 10.1371/journal.pmed.1003913 PMC9767368

[34] Glikman D, Stein M, Shinwell ES. Vaccinating children and adolescents against severe acute respiratory syndrome coronavirus 2 (SARS-CoV-2), updated data from Israel. Acta Paediatr 2022; 111: 189–90. 34657316 10.1111/apa.16157 PMC8653052

[35] Funk A, Florin TA, Kuppermann N, Household transmission dynamics of asymptomatic SARS-CoV-2-infected children: a multinational, controlled case-ascertained prospective study. Clin Infect Dis 2024; 78: 1522–30. 38530249 10.1093/cid/ciae069 PMC11175701

[36] Centers for Disease Control and Prevention. Child vaccination coverage and parental intent for vaccination. https://www.cdc.gov/covidvaxview/weekly-dashboard/child-coverage-vaccination.html (accessed June 27, 2024).

