# Supplementary material for: Vaccine efficacy of NVX-CoV2373 against SARS-CoV-2 infection in adolescents in the USA: an ancillary study to a phase 3, observer-blinded, randomised, placebo-controlled trial
Source: Lancet Microbe. Author manuscript; Available in PMC 2025 Aug 27. (PMC12383606; doi:10.1016/j.lanmic.2024.100984)
Supplement: MMC1 [file NIHMS2051983-supplement-MMC1.pdf]

THE LANCET
Microbe

Supplementary appendix

This appendix formed part of the original submission and has been peer reviewed.
We post it as supplied by the authors.

Supplement to: Deming ME, Brown ER, McArthur MA, et al. Vaccine efficacy of NVX-CoV2373 against SARS-CoV-2 infection in adolescents in the USA: an ancillary study to a phase 3, observer-blinded, randomised, placebo-controlled trial. *Lancet Microbe* 2025. https://doi.org/10.1016/j.lanmic.2024.100984

NVX-CoV2373 efficacy in preventing SARS-CoV-2 infection in adolescents: an ancillary study to a randomized, placebo-controlled, observer-blinded trial

Supplemental materials

Table of Contents

Supplemental Methods: 4

Remote Procedures 4

SARS-CoV-2 testing 5

Adjudication of unscanned swabs 6

Supplemental Tables: 7

Table S1: Characteristics of SNIFF participants in the Primary Analysis Set (PAS, enrolled within 4 weeks of initial vaccination series) and the Post-Crossover Analysis Set (PCAS, enrolled within 4 weeks of the second dose of crossover vaccination) wi... 7

Table S2: Details of incidence rate calculations 9

Table S3: Modified Intention To Treat population compliance to swabbing schedule 10

Supplemental Figures: 11

Figure S1: CDC reported SARS-CoV-2 incidence in adolescents duringSNIFF accrual 11

Figure S2: Geographic distribution of SNIFF sites. 12

Supplemental References 13

SNIFF Study Team Author List 14

SNIFF Study Team 16

Supplement Protocols and Statistical Analysis Plan 27

Protocol_v1.0 (2021-04-02) SNIFF_21-0011 27

Statement of Assurance 29

Statement of Compliance 30

Signature Page 31

Table of Contents 32

List of Figures 35

List of Abbreviations 36

Protocol Summary 39

1 Key Roles 41

2 Background and Scientific Rationale 42

3 Study Design, Objectives and EnDpoints or Outcome Measures 45

4 Study Intervention/Investigational Product 47

5 Selection of Subjects and Study Enrollment and Withdrawal 48

6 Study Procedures 50

7 Description of Clinical and Laboratory Evaluations 53

8 Assessment of Safety 54

9 Human Subjects Protection 55

10 Statistical Considerations 61

11 Source Documents and Access to Source Data/Documents 65
12 Quality Control and Quality Assurance 66
13 Data Handling and Record Keeping 67
14 Clinical Monitoring 69
15 Publication Policy 70
16 AppendiX 71
Protocol_v4.0 (2021-08-18) SNIFF_21-0011 73
Summary of protocol changes v1.0 to v4.0_SNIFF_21-0011 125
SNIFF SAP v1.0 128
SNIFF SAP v2.0 146
Summary of SAP changes v1.0 to v2.0 166
Informed consents and assents 168
Informed Consent_Assent 168
Informed Consent_Parental 170
Informed Consent_Parental_v2 178
Informed consent summary of changes 190
Study Materials 168
Swab self collection instructions 191
Study flier design 191
Study extension fliers (english and spanish) 194
Study completion fliers (english and spanish) 196

Supplemental Methods:

Remote Procedures

Since SARS-CoV-2 infection among adolescents was expected to be a relatively rare event requiring a large sample size, the ancillary study was coupled with the ongoing PREVENT-19 expansion to access 2,232 adolescent participants in the U.S. and benefit from a randomized, double-blinded, placebo-controlled trial design.[1] The methods were guided by the imperative to clearly distinguish the nasal swabs collected for the ancillary study from the Covid-19 case-defining swabs collected for the pivotal expansion study. After a single in-person visit, all study procedures were remote. The in-person visit was used to obtain consent/assent, demonstrate swab collection, register the participant to electronically generate a study identification number for bridging to the parent study, and to activate a customized multifactor authentication Maryland Genomics Sample Scanner smart phone application (MG Scanner™ app v1.2). Written instructions and swabbing supplies for home use were uniformly colored coded and branded with the SNIFF logo (Supplement p.191). A SNIFF study helpline was staffed on weekdays to resolve issues regarding specimen collection, deviations, or delinquency.

The MG Scanner™ app emailed automated reminders to participants when a swab was due or had been missed and provided links to instructions for all study procedures. Participants were instructed to collect nasal swabs at home twice weekly on Mondays and Thursdays throughout the study period. They swabbed both nares at a depth of approximately 1 inch using a single swab and placed the swab in a tube containing buffer media. They scanned the two machine readable codes (barcode and QR code) on each tube using the MG Scanner™ app to link in duplicate their identity and record the date and time of collection. Samples were maintained at room temperature in biohazard bags and shipped weekly in pre-addressed, prepaid United States

Postal Service polyolefin bubble mailers to the Institute of Genome Sciences (IGS), University of Maryland School of Medicine.

SARS-CoV-2 testing

Stability validation testing demonstrated that SARS-CoV-2 RNA remained stable through 10 days in the Qiagen C2.1 buffer (Qiagen – Hilden, Germany) at room temperature. Nasal swabs [COPAN diagnostics 520CS01.BX (Murrieta, California, U.S.) or Puritan Hydraflok 25-3606-H (Guilford, Maine, U.S.)] were tested for SARS-CoV-2 by RT-PCR using a modified version of the CDC 2019 nCoV Real-Time RT-PCR emergency use authorization test assay. 200 µl of nasal swab collection buffer was purified for RNA using modified Qiagen DSP chemistry (Qiagen – Hilden, Germany) on an automated liquid handling platform. RT-PCR was conducted using 1.5 µl RNA template in a 10 µl reaction volume, using three single-plexed reactions for SARS-CoV-2 genes N1 and N3, and human RNaseP. Samples with cycle threshold values (C_T) <40 were scored as detected interpretations for any target. Samples with both N1 and N3 gene detection were determined to be positive for the presence of SARS-CoV-2. Any sample with C_T >=36 was repeated in duplicate and scored based on majority rule of triplicate interpretations. Samples with no detectable RNaseP were considered invalid as having been insufficiently collected by the participant. Samples with N1 C_T <=32 were prepared for whole genome sequencing. Samples with C_T values > 32 were evaluated according to their likelihood to generate a quality genome sequence to maximize the number of viral genomes from unique participants. Selected samples were amplified using ARTIC and VarSkip multiplex amplicon primer sets, followed by NEBNext library preparation (New England Biolabs, Ipswich, MA, USA), library quality assessment, pooling, and multiplexed Illumina NovaSeq 6000 (San Diego, California, USA) paired-end sequencing. Raw SARS-CoV-2 genome sequence data were trimmed for adaptors,

low quality, and amplicon primer sites using a combination of trimmomatic v0.33 and fqtrim v0.9.7 (https://ccb.jhu.edu/software/fqtrim/). Reference-based assemblies were generated using minimap2 for read alignment against the Wuhan reference genome (NC_045512.2) and GATK for assembly refinement and consensus calling. Nextclade[2] and Pangolin[3] were used to generate variant calls and clade and lineage assignments. Viral load was determined by digital PCR using N3 target. Genomic data were analyzed to produce a pangolin lineage assignment (e.g. B.1.1.7) and Greek alpha label (e.g. Alpha) as applicable for variants of interest/concern.

Adjudication of unscanned swabs

Of the 38931 samples received and resulted by IGS by 30-Jan-2022, 2303 (5.9%) had not been scanned by the participant prior to shipping (unscanned swabs). Of these, 62 unscanned swabs from 11 participants were positive for SARS-CoV-2 by PCR, and therefore might contribute to the primary endpoint. A four-person blinded adjudication committee reviewed the timing of the receipt of both scanned and unscanned swabs from the 11 participants with the goal of assigning a date to the first swab with detected SARS-CoV-2. In brief, 4 swabs were excluded because they were received in January 2022, the subjects having provided swabs through the end of study that were negative. An additional 4 positive swabs did not change the first detected date, having been received after a prior scanned swab with detected SARS-CoV-2. Two swabs were assigned with good confidence to a swabbing window based on regularly received negative swabs scanned by the participant. One swab was assigned to a midpoint between the time received by the lab and prior received scanned swab. All decisions were made with all four committee members in agreement.

Supplemental Tables:

Table S1: Characteristics of SNIFF participants in the Primary Analysis Set (PAS, enrolled within 4 weeks of initial vaccination series) and the Post-Crossover Analysis Set (PCAS, enrolled within 4 weeks of the second dose of crossover vaccination) within the modified intention to treat population, [01-June-2021 through 17-Dec-2021]

	Primary analysis set (PAS)			Post-crossover analysis set (PCAS)		
	NVX-CoV2373	Placebo	All Participants	Early NVX-CoV2373	Delayed vaccine	All Participants
**Participants Enrolled**	471	220	691	609	277	886
**Participant Age (years)**						
Mean (SD)	14.0 (1.4)	14.0 (1.4)	14.0 (1.4)	14.0 (1.4)	14.0 (1.4)	14.0 (1.4)
Median	14.0	14.0	14.0	14.0	14.0	14.0
25th, 75th %tile	13, 15	13, 15	13, 15	13, 15	13, 15	13, 15
Min, Max	12, 17	12, 17	12, 17	12, 17	12, 17	12, 17
**Participant Age Group**						
12 - <15	295 (63%)	144 (65%)	439 (64%)	388 (64%)	175 (63%)	563 (64%)
15 - <18	176 (37%)	76 (35%)	252 (36%)	221 (36%)	102 (37%)	323 (36%)
**Sex at birth**						
Male	240 (51%)	111 (50%)	351 (51%)	319 (52%)	144 (52%)	463 (52%)
Female	231 (49%)	109 (50%)	340 (49%)	290 (48%)	133 (48%)	423 (48%)
**Latino or Hispanic ethnicity**						
Yes	77 (16%)	36 (16%)	113 (16%)	109 (18%)	52 (19%)	161 (18%)
No	393 (83%)	184 (84%)	577 (84%)	498 (82%)	225 (81%)	723 (82%)
Not Reported	0 (0%)	0 (0%)	0 (0%)	0 (0%)	0 (0%)	0 (0%)
Unknown	1 (<1%)	0 (0%)	1 (<1%)	2 (<1%)	0 (0%)	2 (<1%)
**Race**						
White	362 (77%)	161 (73%)	523 (76%)	466 (77%)	201 (73%)	667 (75%)
Black or African American	56 (12%)	32 (15%)	88 (13%)	71 (12%)	33 (12%)	104 (12%)

American Indian or Alaska Native	2 (<1%)	0 (0%)	2 (<1%)	8 (1%)	5 (2%)	13 (1%)
Native Hawaiian or Other Pacific Islander	1 (<1%)	1 (<1%)	2 (<1%)	1 (<1%)	1 (<1%)	2 (<1%)
Asian	15 (3%)	9 (4%)	24 (3%)	22 (4%)	15 (5%)	37 (4%)
Mixed Origin	33 (7%)	16 (7%)	49 (7%)	38 (6%)	20 (7%)	58 (7%)
Not Reported	2 (<1%)	1 (<1%)	3 (<1%)	3 (<1%)	2 (1%)	5 (1%)
**Participant Height (cm)**						
Mean (SD)	164.8 (10.7)	163.7 (9.7)	164.5 (10.4)	164.9 (10.4)	163.7 (10.5)	164.5 (10.4)
Median	165.0	162.6	164.0	165.0	163.3	164.0
25th, 75th %tile	158, 171	158, 170	158, 171	159, 171	157, 171	158, 171
Min, Max	99, 196	126, 193	99, 196	99, 196	124, 193	99, 196
**Participant Weight (kg)**						
Mean (SD)	66.1 (20.5)	62.4 (18.7)	64.9 (20.0)	66.4 (20.7)	62.5 (17.9)	65.2 (19.9)
Median	62.0	57.6	61.2	62.6	59.0	61.2
25th, 75th %tile	52, 77	51, 70	52, 75	52, 78	51, 70	51, 76
Min, Max	32, 154	30, 174	30, 174	32, 152	26, 121	26, 152
**Participant BMI (kg/cm^{2})**						
Mean (SD)	24.2 (6.7)	23.3 (6.6)	23.9 (6.7)	24.3 (6.6)	23.2 (6.0)	23.9 (6.4)
Median	22.6	21.5	22.3	22.8	21.8	22.4
25th, 75th %tile	19, 28	19, 26	19, 27	19, 28	19, 26	19, 27
Min, Max	14, 53	10, 64	10, 64	14, 53	10, 41	10, 53
**Student Attending School in Person**						
Yes	308 (65%)	142 (65%)	450 (65%)	403 (66%)	186 (67%)	589 (66%)
No	163 (35%)	78 (35%)	241 (35%)	206 (34%)	91 (33%)	297 (34%)

Table S2: Details of incidence rate calculations

Analysis set, Censoring	Arm	Number of participants	Number of incident endpoints	Person-years	Incidence rate per 100 person-years (95% CI)	VE against infection % (95% CI)	p-value	Meet PREVENT-19 endpoints (N=)
**Primary Analysis Set** (Censor at Dose #2 of crossover)	Vaccine	466	13	87.2	14.9 (7.9, 25.5)	73.5 (47.1, 86.7)	0.0002	4
	Placebo	216	21	38.7	54.2 (33.6, 82.9)			7
**Primary Analysis Set Censored at Dose #1 of crossover** (Sensitivity analysis)	Vaccine	464	5	62.6	8.0 (2.6, 18.6)	77.8 (35.0, 92.4)	0.0060	1
	Placebo	216	10	28.4	35.3 (16.9, 64.8)			4
**modified Intention to Treat** (Censor at Dose #2 of crossover)	Vaccine	628	16	110.3	14.5 (8.3, 23.5)	74.8 (53.5, 86.4)	<0.0001	4
	Placebo	302	28	50.1	55.8 (37.1, 80.7)			9
**modified Intention to Treat, Censored at Dose #1 of crossover** (Sensitivity analysis)	Vaccine	620	5	76.3	6.6 (2.1, 15.3)	82.6 (51.1, 93.8)	0.0009	1
	Placebo	301	13	35.3	36.8 (19.6, 62.9)			5

Table S3: Modified Intention To Treat population compliance to swabbing schedule

	Immediate NVX-CoV2373	Placebo / Delayed NVX-CoV2373	All participants
Participants enrolled	646	311	957
Number of expected swab collection windows[1]	30734	14518	45252
Number of received swab collection windows (%)[2]	19417 (63.2%)	9072 (62.5%)	28489 (63.0%)
Participant-level percentage compliance to swab collection windows[2]			
N[3]	639	305	944
Mean % (SD)	63.5 (27.3)	63.2 (28.0)	63.4 (27.5)
Median %	70.9	71.7	71.2
25th, 75th percentile	41, 87	40, 87	41, 87
Min, Max %	4, 100	2, 100	2, 100

[1] Expected number of collection windows between enrollment and study discontinuation

[2] Unscanned swabs and swabs collected after study discontinuation are not included. Multiple swabs scanned in the same collection window are only counted once.

[3] Number of participants submitting at least one swab within an expected swab collection window

Supplemental Figures:

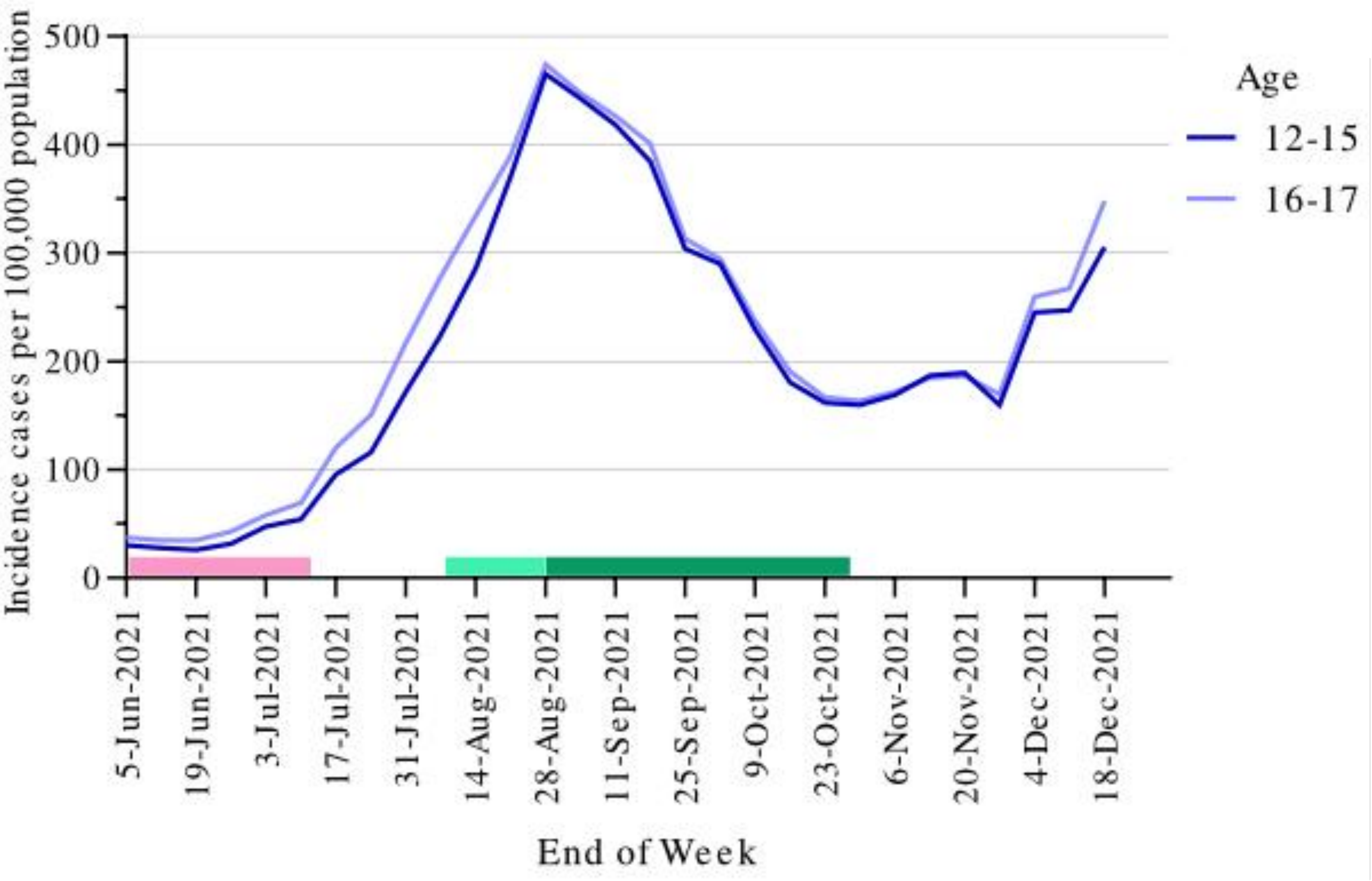

Figure S1: CDC reported SARS-CoV-2 incidence in adolescents during SNIFF accrual. Incident cases of SARS-CoV-2 among adolescents (CDC age groups 12-15 and 16-17 years) during the SNIFF enrollment period. Red bar = PREVENT-19 first NVX-CoV2373 vaccine series; Teal bars = crossover NVX-CoV2373 vaccinations. (source: https://covid.cdc.gov/covid-data-tracker/#demographicsovertime, accessed 2022-12-06).

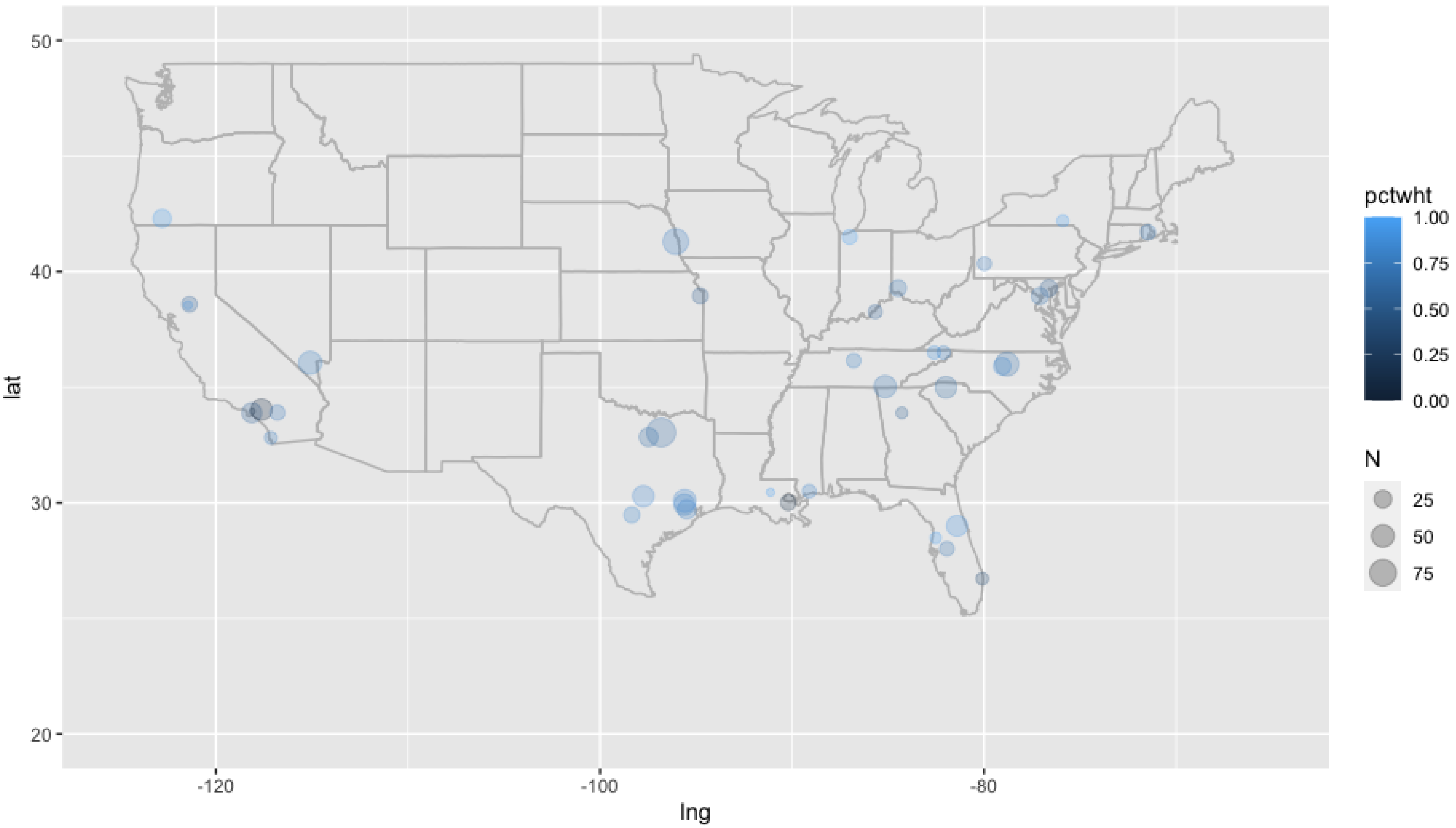

Figure S2: Geographic distribution of SNIFF sites.

47 U.S. sites participated in SNIFF enrolling 1196 participants. Dots indicate site location with size proportional to number of participants enrolled, ranging from 2 to 92 participants.

Supplemental References

1. Anez G, Dunkle LM, Gay CL, et al. Safety, Immunogenicity, and Efficacy of the NVX-CoV2373 COVID-19 Vaccine in Adolescents: A Randomized Clinical Trial. JAMA Netw Open 2023;6(4):e239135. DOI: 10.1001/jamanetworkopen.2023.9135.
2. Aksamentov I, Roemer C, Hodcroft E, Neher R. Nextclade: clade assignment, mutation calling and quality control for viral genomes. Journal of Open Source Software 2021;6(67):3773. DOI: 10.21105/joss.03773.
3. O'Toole A, Scher E, Underwood A, et al. Assignment of epidemiological lineages in an emerging pandemic using the pangolin tool. Virus Evol 2021;7(2):veab064. DOI: 10.1093/ve/veab064.

SNIFF Study Team Author List

*First Name and Middle Initial(s)	*Last Name	Academic Degrees	Institution	Location (city, state/province, country)
Meagan E.	Deming	MD, PhD	University of Maryland School of Medicine	Baltimore, MD
Elizabeth R.	Brown	ScD	Fred Hutchinson Cancer Research Center	Seattle, WA
Monica A.	McArthur	MD, PhD	University of Maryland School of Medicine	Baltimore, MD
Stephanie J	Schrag	Dphil	Centers for Disease Control and Prevention	Atlanta, GA
Melissa	Arvay	PhD, MPH	Centers for Disease Control and Prevention	Atlanta, GA
Mike	Humphrys	MS	Institute for Genome Sciences	Baltimore, MD
Jacques	Ravel	PhD	Institute for Genome Sciences	Baltimore, MD
Jeffrey	Adelglass	MD	Research Your Health	Plano, TX
Brandon	Essink	MD	Meridian Clinical Research	Omaha, NE
David	Musante	MD	M3 Emerging Medical Research, LLC	Durham, NC
Rebecca	Maguire		University of Maryland School of Medicine	Baltimore, MD
Richard	Gorman	MD	Biomedical Advanced Research and Development Authority	Washington, DC
Elizabeth	Formentini	MSN, MBA	Biomedical Advanced Research and Development Authority	Washington, DC
Robin	Mason	MS, MBA	Biomedical Advanced Research and Development Authority	Washington, DC
Merlin L.	Robb	MD	Henry M. Jackson Foundation for the Advancement of Military Medicine	Bethesda, MD
Kathleen M.	Neuzil	MD, MPH	University of Maryland School of Medicine	Baltimore, MD
Rekha R	Rapaka	MD, PhD	University of Maryland School of Medicine	Baltimore, MD
Peter	Wolff	MHA	National Institutes of Health	Washington, DC
Karen L	Kotloff	MD	University of Maryland School of Medicine	Baltimore, MD
Ronald	Ackerman	MD	Comprehensive Clinical Research	West Palm Beach, FL
Elizabeth	Barranco-Santana	MD	Ponce School of Medicine/NIAID (UM1AI148685)	Ponce, Puerto Rico
Laurence	Chu	MD	Benchmark Research	Austin, TX
Stuart H.	Cohen	MD	University of California, Davis Medical Center	Sacramento, CA
Wendy	Daly	MD	Brownsboro Park Pediatrics	Louisville, Kentucky
Kenneth	Etokhana	MD	Tekton Research	San Antonio, TX
Cynthia	Gay	MD	University of North Carolina School Of Medicine	Chapel Hill, NC
Greg	Hachigian	MD	Benchmark Research	Sacramento, CA

Robert	Jeanfreau	MD	Med Pharmics, LLC	Metairie, Louisiana
Vicki E.	Miller	MD, MPH	DM Clinical Research	Tomball, TX
William	Seger	MD	Benchmark Research	Fort Worth, TX
Joanna	Sexter	MD	Meridian Clinical Research	Washington, DC
Marian E.	Shaw	MD	Velocity Clinical Research	Meridian, ID
Kenneth	Steil	MD	Cognitive Clinical Trials	Phoenix, AZ
Christine B.	Turley	MD	Atrium Health	Matthews, NC
Michael	Waters	MD	Velocity Clinical Research	Chula Vista, CA

SNIFF Study Team

Site	*First Name and Middle Initial(s)	*Last Name	*Suffix (eg, Jr, III)	Academic Degrees	Institution	Location (city, state/province, country)	Role or Contribution, eg, chair, principal investigator
US003	Carlos	Fierro		MD	Johnson County Clin-Trials	Lenexa, KS	Principal investigator
US003	Natalya	Amrine			Johnson County Clin-Trials	Lenexa, KS	Study Coordinator
US012	Marian E.	Shaw		MD	Velocity Clinical Research	Meridian, ID	Principal investigator
US012	Laura	Crenhaw			Velocity Clinical Research	Meridian, ID	Study Coordinator
US012	Audra	Weslowski			Velocity Clinical Research	Meridian, ID	Study Coordinator
US012	Nichole	Wichman			Velocity Clinical Research	Meridian, ID	Study Coordinator
US012	Antonio	Navarrete			Velocity Clinical Research	Meridian, ID	Study Coordinator
US018	Brandon	Essink		MD	Meridian Clinical Research	Omaha, NE	Principal investigator
US018	Hannah	Harrington		MPH	Meridian Clinical Research	Omaha, NE	Study coordinator
US029	Stephan	Sharp		MD	Clinical Research Associates	Nashville, TN	Principal investigator
US029	Stacy	Cox		APN, RN	Clinical Research Associates	Nashville, TN	Study Coordinator
US029	Karen	Mullen			Clinical Research Associates	Nashville, TN	Study team
US039	Robert	Jeanfreau		MD	Med Pharmics, LLC	Metairie, Louisiana	Principal investigator
US039	Susan	Tortorich			Med Pharmics, LLC	Metairie, Louisiana	Study team
US039	Katelyn	Jackson			Med Pharmics, LLC	Metairie, Louisiana	Study team
US047	Laurence	Chu		MD	Benchmark Research	Austin, TX	Principal investigator
US047	Jennifer	Montes			Benchmark Research	Austin, TX	Study team
US060	William	Seger		MD	Benchmark Research	Fort Worth, TX	Principal investigator
US060	Deborah	Devlin		LVN, CCRC	Benchmark Research	Fort Worth, TX	Study coordinator
US060	John	Villegas			Benchmark Research	Fort Worth, TX	Study coordinator
US060	Virginia	Loudermilk			Benchmark Research	Fort Worth, TX	Study coordinator
US060	Crystal	Starr			Benchmark Research	Fort Worth, TX	Study team
US060	Alma	Guel			Benchmark Research	Fort Worth, TX	Study team
US060	Beverly	Ewing			Benchmark Research	Fort Worth, TX	Study team
US060	Tisha	Davis			Benchmark Research	Fort Worth, TX	Study team
US060	Tasha	Todd			Benchmark Research	Fort Worth, TX	Study team
US060	Jailyn	Reyes			Benchmark Research	Fort Worth, TX	Study team
US060	John Daniel	Villegas Jr			Benchmark Research	Fort Worth, TX	Study team
US060	Logan	Kimball			Benchmark Research	Fort Worth, TX	Study team

US060	Moahmmed	Antwi		PA-C	Benchmark Research	Fort Worth, TX	Study team
US060	Ruth	Reyes			Benchmark Research	Fort Worth, TX	Study team
US060	Maria	Seger			Benchmark Research	Fort Worth, TX	Study team
US073	Vicki E.	Miller		MD	DM Clinical Research	Tomball, TX	Principal investigator
US073	Pauline	Ngban			DM Clinical Research	Tomball, TX	Study coordinator
US073	Zainab	Rizvi			DM Clinical Research	Tomball, TX	Study coordinator
US074	Bernard	Grunstra		MD	Internal Medicine and Pediatric Associates of Bristol, PC	Bristol, TN	Principal investigator
US074	Amy	Dye			Internal Medicine and Pediatric Associates of Bristol, PC	Bristol, TN	Study coordinator
US076	Larkin Tyler	Wadsworth	III	MD	Sundance Clinical Research	St. Louis, MO	Principal investigator
US076	Anya	Penly			Sundance Clinical Research	St. Louis, MO	Study Coordinator
US076	Sophia	Bolakas			Sundance Clinical Research	St. Louis, MO	Study Coordinator
US076	Christy	Shultz			Sundance Clinical Research	St. Louis, MO	Study Coordinator
US076	Andrea	Deffenbaugh			Sundance Clinical Research	St. Louis, MO	Study Coordinator
US076	Angela	Kean			Sundance Clinical Research	St. Louis, MO	Study Coordinator
US098	Paul G.	Matherne		MD	MedPharmics	Gulfport, MS	Principal investigator
US098	Lauren	Newhouse			MedPharmics	Gulfport, MS	Study coordinator
US098	Lakeyla	Bates			MedPharmics	Gulfport, MS	Study coordinator
US142	Michael	Waters		MD	Velocity Clinical Research	Chula Vista, CA	Principal investigator
US142	Dalia	Tovar		MA	Velocity Clinical Research	Chula Vista, CA	Study Coordinator
US143	Jeffrey	Adelglass		MD, FACS	Research Your Health	Plano, TX	Principal investigator
US143	Kristine Jane	Kucera		MPAS, DHS	Research Your Health	Plano, TX	Sub-Investigator
US143	Waseem	Chughtai		B.S., M.B.B.S, CCRC	Research Your Health	Plano, TX	clinical research coordinator
US143	Anuja	Sathe		MSc	Research Your Health	Plano, TX	clinical research coordinator
US143	Chiedza	Mutindori		BSc	Research Your Health	Plano, TX	clinical research coordinator
US143	Daylia	Hollins		C.N.A.	Research Your Health	Plano, TX	Coordinator Assistant
US143	Sabrina	Flowers		BSc	Research Your Health	Plano, TX	Data Coordinator
US145	Gregg	Lucksinger		MD	Velocity Clinical Research	Medford , Oregon	Principal investigator
US145	Audrey	Kuehl			Velocity Clinical Research	Medford , Oregon	Study team
US149	Bruce	Gebhardt		MD	Sterling Research Group	Cincinnati, OH	Principal investigator
US149	Heather	Siliven			Sterling Research Group	Cincinnati, OH	Site Manager

US149	Cindy	Wright		Sterling Research Group	Cincinnati, OH	Study coordinator
US152	Bruce	Rankin	DO	University Clinical Research-Deland, LLC dba Accel Research Sites	Deland, FL	Principal investigator
US152	Stacie	Evans		University Clinical Research-Deland, LLC dba Accel Research Sites	Deland, FL	Study Coordinator
US157	Greg	Hachigian	MD	Benchmark Research	Sacramento, CA	Principal investigator
US157	Yaman	Daramarathne		Benchmark Research	Sacramento, CA	Study team
US157	Lisa	Anderson		Benchmark Research	Sacramento, CA	Study team
US157	Sara	Malakzay		Benchmark Research	Sacramento, CA	Study team
US157	Lisa	Stenhouse		Benchmark Research	Sacramento, CA	Study team
US159	James	Andersen	MD	Accel Research Sites	Lakeland, FL	Principal investigator
US159	Amy	Medina	ADN, BS	Accel Research Sites	Lakeland, FL	Study team
US159	Colleen	Figueroa		Accel Research Sites	Lakeland, FL	Study team
US159	Diana	Holmes		Accel Research Sites	Lakeland, FL	Study team
US159	Courtney	Smith		Accel Research Sites	Lakeland, FL	Study team
US162	David	Fried	MD	Velocity Clinical Research	Warwick, RI	Principal investigator
US162	Minhah	Ghouri		Velocity Clinical Research	Warwick, RI	Study Coordinator
US162	Monica	Freeman		Velocity Clinical Research	Warwick, RI	Study Coordinator
US163	Veronica	Fragoso	MD	Texas Center for Drug Development	Houston, TX	Principal investigator
US163	Maria Gabriela	Becerra Teran	MD	Texas Center for Drug Development	Houston, TX	Study Coordinator
US163	Cecilia L.	McKeown-Biagas	M.D.	Texas Center for Drug Development	Houston, TX	Study team
US163	Tracy Jean	Kowalski	RN, FNP-C	Texas Center for Drug Development	Houston, TX	Study team
US163	Vicki E.	Miller	MD, MPH	Texas Center for Drug Development	Houston, TX	Study team
US163	Chen-Ho	Yang	PA	Texas Center for Drug Development	Houston, TX	Study team
US163	Shakira Laine	Barr	RN, FNP-C	Texas Center for Drug Development	Houston, TX	Study team
US163	Toni Irene	White	M.D.	Texas Center for Drug Development	Houston, TX	Study team
US163	Bonnie Elizabeth	Colville	D.O.	Texas Center for Drug Development	Houston, TX	Study team

US163	Danitra Brenique	Glasper	MSN, APRN, FNP-C	Texas Center for Drug Development	Houston, TX	Study team
US163	Chance	Caddell		Texas Center for Drug Development	Houston, TX	Study team
US163	Diana	Chehab		Texas Center for Drug Development	Houston, TX	Study team
US163	Joanna	Quezon		Texas Center for Drug Development	Houston, TX	Study team
US163	Maryam	Rabbani		Texas Center for Drug Development	Houston, TX	Study team
US163	Akram	Assaf		Texas Center for Drug Development	Houston, TX	Study team
US163	Ayla	Perez		Texas Center for Drug Development	Houston, TX	Study team
US163	Berenice	Ferrero		Texas Center for Drug Development	Houston, TX	Study team
US163	Biman	Goswani		Texas Center for Drug Development	Houston, TX	Study team
US163	Dean	Jang		Texas Center for Drug Development	Houston, TX	Study team
US163	Dustin	McFadden		Texas Center for Drug Development	Houston, TX	Study team
US163	Elton	Oliveira		Texas Center for Drug Development	Houston, TX	Study team
US163	Enya	Rentas-Sherman		Texas Center for Drug Development	Houston, TX	Study team
US163	Julian	Edmonson		Texas Center for Drug Development	Houston, TX	Study team
US163	Laura N. Plaza	Grisanty		Texas Center for Drug Development	Houston, TX	Study team
US163	Mary	Rogers		Texas Center for Drug Development	Houston, TX	Study team
US163	Nazanin	Zarinkamar		Texas Center for Drug Development	Houston, TX	Study team
US163	Olga	Konshina		Texas Center for Drug Development	Houston, TX	Study team
US163	Raquel	Araujo-Gutierrez		Texas Center for Drug Development	Houston, TX	Study team
US163	Sadaf	Batla		Texas Center for Drug Development	Houston, TX	Study team
US163	Sauleha	Husain		Texas Center for Drug Development	Houston, TX	Study team

US163	Scott	Ward	Texas Center for Drug Development	Houston, TX	Study team
US163	Simeen	Khan	Texas Center for Drug Development	Houston, TX	Study team
US163	Teodoro	Seminario	Texas Center for Drug Development	Houston, TX	Study team
US163	Elisa	Moralez	Texas Center for Drug Development	Houston, TX	Study team
US163	Frances	Saubon	Texas Center for Drug Development	Houston, TX	Study team
US163	Jenny	Torres	Texas Center for Drug Development	Houston, TX	Study team
US163	William	Fernandez	Texas Center for Drug Development	Houston, TX	Study team
US163	Joel	Cano	Texas Center for Drug Development	Houston, TX	Study team
US163	Kendra	Rogers	Texas Center for Drug Development	Houston, TX	Study team
US163	Quiana	Wilson	Texas Center for Drug Development	Houston, TX	Study team
US163	Kara	Sikes	Texas Center for Drug Development	Houston, TX	Study team
US163	Felicia	Ardoin	Texas Center for Drug Development	Houston, TX	Study team
US163	Karina	Sainz	Texas Center for Drug Development	Houston, TX	Study team
US163	Abdeali	Dalal	Texas Center for Drug Development	Houston, TX	Study team
US163	Ana	Rueda	Texas Center for Drug Development	Houston, TX	Study team
US163	Crystal	Reese	Texas Center for Drug Development	Houston, TX	Study team
US163	Bernardo Martinez	Leal	Texas Center for Drug Development	Houston, TX	Study team
US163	Leena	Mir	Texas Center for Drug Development	Houston, TX	Study team
US163	Lucia	Almaguer	Texas Center for Drug Development	Houston, TX	Study team
US163	Shamarrian	Hampton	Texas Center for Drug Development	Houston, TX	Study team
US163	Deep	Patel	Texas Center for Drug Development	Houston, TX	Study team

US163	Faryal	Mahmood		Texas Center for Drug Development	Houston, TX	Study team
US163	Hansol	Jang		Texas Center for Drug Development	Houston, TX	Study team
US163	Norma	Gonzalez		Texas Center for Drug Development	Houston, TX	Study team
US163	Stacey	Montero		Texas Center for Drug Development	Houston, TX	Study team
US163	Stacy	Villareal		Texas Center for Drug Development	Houston, TX	Study team
US163	Mohammad	Rizvi		Texas Center for Drug Development	Houston, TX	Study team
US163	Luis	Munoz		Texas Center for Drug Development	Houston, TX	Study team
US163	Cara	Woodham		Texas Center for Drug Development	Houston, TX	Study team
US163	Sarah Lucky	Dania		Texas Center for Drug Development	Houston, TX	Study team
US163	Ijeoma	Okoko		Texas Center for Drug Development	Houston, TX	Study team
US163	Andrea	Juanillo		Texas Center for Drug Development	Houston, TX	Study team
US163	Sylvia Gonzalez	Cooper		Texas Center for Drug Development	Houston, TX	Study team
US163	Sara	Solorzano		Texas Center for Drug Development	Houston, TX	Study team
US163	Mirella	Melendez		Texas Center for Drug Development	Houston, TX	Study team
US164	Robert J.	Buynak	MD	Velocity Clinical Research	Valparaiso, IN	Principal investigator
US164	Rachel	McNeal		Velocity Clinical Research	Valparaiso, IN	Study team
US164	Mark	Yarosz		Velocity Clinical Research	Valparaiso, IN	Study team
US168	Mark	McKenzie	MD	WR Clinsearch, LLC	Chattanooga, TN	Principal investigator
US168	Christy	Schmeck		WR Clinsearch, LLC	Chattanooga, TN	Study coordinator
US171	Ronald	Ackerman	MD	Comprehensive Clinical Research	West Palm Beach, FL	Principal investigator
US171	Jamie	Ackerman	BA	Comprehensive Clinical Research	West Palm Beach, FL	Study team
US171	Florida	Aristy	APRN	Comprehensive Clinical Research	West Palm Beach, FL	Study team
US171	Tomeko	Heard		Comprehensive Clinical Research	West Palm Beach, FL	Study team

US171	Diana	Mann		Comprehensive Clinical Research	West Palm Beach, FL	Study team
US180	Donald M.	Brandon	MD	California Research Foundation	San Diego, CA	Principal investigator
US180	Charlene	Cruz		California Research Foundation	San Diego, CA	Study team
US180	Mairead	Hawkins		California Research Foundation	San Diego, CA	Study team
US180	Lorraine	Boggs		California Research Foundation	San Diego, CA	Study team
US184	Judith	Kirstein	MD	Velocity Clinical Research	Banning, CA	Principal investigator
US184	Nolan	Mackey		Velocity Clinical Research	Banning, CA	Study coordinator
US184	Julie	Kasarjian	MD	Velocity Clinical Research	Banning, CA	Study team
US184	Krista	Foster		Velocity Clinical Research	Banning, CA	Study team
US184	Nicole	Abels		Velocity Clinical Research	Banning, CA	Study team
US184	Brandy	Lopez		Velocity Clinical Research	Banning, CA	Study team
US184	Crystle	Rajania	Dr	Velocity Clinical Research	Banning, CA	Study team
US185	David	Musante	MD	M3 Emerging Medical Research, LLC	Durham, NC	Principal investigator
US185	Shandelle	Parker		M3 Emerging Medical Research, LLC	Durham, NC	Study coordinator
US185	William P.	Silver	MD	M3 Emerging Medical Research, LLC	Durham, NC	Study team
US185	Linda R.	Belhorn	MD	M3 Emerging Medical Research, LLC	Durham, NC	Study team
US185	Nicholas A.	Viens	MD	M3 Emerging Medical Research, LLC	Durham, NC	Study team
US185	David	Dellaero	MD	M3 Emerging Medical Research, LLC	Durham, NC	Study team
US185	Elizabeth	Wilkers	MD	M3 Emerging Medical Research, LLC	Durham, NC	Study team
US191	Meagan E	Deming	MD, PhD	University of Maryland School of Medicine	Baltimore, MD	Principal investigator
US191	Monica	McArthur	MD, PhD	University of Maryland School of Medicine	Baltimore, MD	Principal investigator
US191	Shirley	George	MS	University of Maryland School of Medicine	Baltimore, MD	Study team
US191	Karen L	Kotloff	MD	University of Maryland School of Medicine	Baltimore, MD	Study team
US191	Rekha R	Rapaka	MD, PhD	University of Maryland School of Medicine	Baltimore, MD	Study team
US191	Rebecca	Maguire		University of Maryland School of Medicine	Baltimore, MD	Study team

US195	Cynthia	Gay	MD	University of North Carolina School Of Medicine	Chapel Hill, NC	Principal investigator
US195	Erin	Hoffman		University of North Carolina School Of Medicine	Chapel Hill, NC	Study Coordinator
US197	Kenneth	Etokhana	MD	Tekton Research	San Antonio, TX	Principal investigator
US197	Krunal	Khamkar		Tekton Research	San Antonio, TX	Study Coordinator
US197	Miranda	Ruiz		Tekton Research	San Antonio, TX	Study Coordinator
US197	Breana	Riley		Tekton Research	San Antonio, TX	Study Coordinator
US197	Hannah	Alsobrooks		Tekton Research	San Antonio, TX	Study Coordinator
US197	Xavier	Fajardo		Tekton Research	San Antonio, TX	Study Coordinator
US215	Elizabeth	Barranco-Santana	MD	Ponce School of Medicine/NIAID (UM1AI148685)	Ponce, Puerto Rico	Principal investigator
US215	Irmaris	Arroyo		Ponce School of Medicine/NIAID (UM1AI148685)	Ponce, Puerto Rico	Study team
US215	Brenda	Garcia		Ponce School of Medicine/NIAID (UM1AI148685)	Ponce, Puerto Rico	Study team
US215	Michele	Irizarry		Ponce School of Medicine/NIAID (UM1AI148685)	Ponce, Puerto Rico	Study team
US215	Alice	Rodriguez		Ponce School of Medicine/NIAID (UM1AI148685)	Ponce, Puerto Rico	Study team
US215	Velma	Franceschinni		Ponce School of Medicine/NIAID (UM1AI148685)	Ponce, Puerto Rico	Study team
US215	Jackeline	Torres		Ponce School of Medicine/NIAID (UM1AI148685)	Ponce, Puerto Rico	Study team
US215	Lydiet	Dedos		Ponce School of Medicine/NIAID (UM1AI148685)	Ponce, Puerto Rico	Study team
US215	Nancy	Jimenez		Ponce School of Medicine/NIAID (UM1AI148685)	Ponce, Puerto Rico	Study team
US227	Christine B.	Turley	MD	Atrium Health	Matthews, NC	Principal investigator
US227	Jona	Bauer		Atrium Health/Wake Forest School of Medicine	Charlotte, NC	Study Coordinator
US227	Andrew	McWilliams	MD	Atrium Health/Wake Forest School of Medicine	Charlotte, NC	Study team
US227	Lewis	McCurdy	MD	Atrium Health/Wake Forest School of Medicine	Charlotte, NC	study team
US227	Tiffany	Esinhart	PA-C	Atrium Health/Wake Forest School of Medicine	Charlotte, NC	Study team
US227	Natasha	Montoya	APRN	Atrium Health/Wake Forest School of Medicine	Charlotte, NC	Study team
US227	Shamika	Huskey	FNP	Atrium Health/Wake Forest School of Medicine	Charlotte, NC	Study team

US227	Zeynep	Alimchandani		Atrium Health/Wake Forest School of Medicine	Charlotte, NC	Study Team
US227	Veena	Sampathkumar		Atrium Health/Wake Forest School of Medicine	Charlotte, NC	Study team
US227	Cedrick	Griner		Atrium Health/Wake Forest School of Medicine	Charlotte, NC	Study Team
US227	Kathleen	Barber	PA-C	Atrium Health/Wake Forest School of Medicine	Charlotte, NC	Study team
US227	Tonisha	Brown		Atrium Health/Wake Forest School of Medicine	Charlotte, NC	Study team
US227	Robin	Muller		Atrium Health/Wake Forest School of Medicine	Charlotte, NC	Study team
US227	Amina	Ahmed	MD	Atrium Health/Wake Forest School of Medicine	Charlotte, NC	Study team
US227	Saifelnasr	Mohamed		Atrium Health/Wake Forest School of Medicine	Charlotte, NC	Study team
US227	Keerti	Dantuluri	MD, MPH	Atrium Health/Wake Forest School of Medicine	Charlotte, NC	Study team
US227	Brian	Lurie	MD, MPH	Atrium Health/Wake Forest School of Medicine	Charlotte, NC	Study team
US227	Leena	Paul	FNP	Atrium Health/Wake Forest School of Medicine	Charlotte, NC	Study team
US230	Stuart H.	Cohen	MD	University of California, Davis Medical Center	Sacramento, CA	Principal investigator
US230	Sonja	Neumeister	MPH	University of California, Davis Medical Center	Sacramento, CA	Study team
US251	Khozema	Palanpurwala	MD	DM Clinical Research - Pediatric Healthcare of NW Houston, P.A.	Houston, Texas	Principal investigator
US251	Vartika	Jain		DM Clinical Research - Pediatric Healthcare of NW Houston, P.A.	Houston, Texas	Project Manager
US251	Imran	Khan		DM Clinical Research - Pediatric Healthcare of NW Houston, P.A.	Houston, Texas	Site Manager
US251	Zehra	Palanpurwala		DM Clinical Research - Pediatric Healthcare of NW Houston, P.A.	Houston, Texas	Study Manager
US255	Wendy	Daly	MD	Brownsboro Park Pediatrics	Louisville, Kentucky	Principal investigator
US255	Kimberly	Downs	MD	Brownsboro Park Pediatrics	Louisville, Kentucky	Study coordinator
US256	Teresita	Salazar	MD	Coast Clinical Research, LLC	Bellflower, CA	Principal investigator
US256	Dianna	Trespuer		Coast Clinical Research, LLC	Bellflower, CA	Study Coordinator
US256	Filipinas	Vitug		Coast Clinical Research, LLC	Bellflower, CA	Study Coordinator
US256	Semine	David		Coast Clinical Research, LLC	Bellflower, CA	Study team

US257	Michael	Levin	MD	Clinical Research Center of Nevada	Henderson, NV	Principal investigator
US257	Meghan	Caldron		Clinical Research Center of Nevada	Henderson, NV	Study coordinator
US257	Melissa	Brenner		Clinical Research Center of Nevada	Henderson, NV	Study coordinator
US257	Brennan	Opanasenko		Clinical Research Center of Nevada	Henderson, NV	Study coordinator
US258	Bryce A.	Palchick	MD	Preferred Primary Care Physicians	Pittsburgh, PA	Principal investigator
US258	Nathan L.	Bennett	MD	Preferred Primary Care Physicians	Pittsburgh, PA	Study team
US258	Shari E.	Rozen	MD	Preferred Primary Care Physicians	Pittsburgh, PA	Study team
US258	Michael E.	Gates	MD	Preferred Primary Care Physicians	Pittsburgh, PA	Study team
US258	Kyla	Shultz	RN	Preferred Primary Care Physicians	Pittsburgh, PA	Study team
US258	Sarah M.	Dobrosky		Preferred Primary Care Physicians	Pittsburgh, PA	Study team
US258	Jill	Waldo		Preferred Primary Care Physicians	Pittsburgh, PA	Study team
US258	Laura	Pellegrini		Preferred Primary Care Physicians	Pittsburgh, PA	Study team
US259	Joseph	Ley	MD	Holston Medical Group	Kingsport, TN	Principal investigator
US259	Ashley	Helton		Holston Medical Group	Kingsport, TN	Study coordinator
US261	Imad	Jandali	MD	Asclepes Research Centers	Spring Hill, FL	Principal investigator
US261	Katie	Leonard		Asclepes Research Centers	Spring Hill, FL	Study coordinator
US261	David	Daniels		Asclepes Research Centers	Spring Hill, FL	Study coordinator
US261	Maryam	Belavilas	MD	Asclepes Research Centers	Spring Hill, FL	Study team
US262	Rosario	Retino	MD	Orange County Research Institute	Ontario, CA	Principal investigator
US262	Alex	Coronel		Orange County Research Institute	Ontario, CA	Study Coordinator
US263	Adebayo	Akinsola	MD	Tekton Research	Chamblee, GA	Principal investigator
US263	Olamide	Lawson		Tekton Research	Chamblee, GA	Study coordinator
US263	Toluwa	Ojo		Tekton Research	Chamblee, GA	Study coordinator
US267	Charles	Fogarty	MD	Spartanburg Medical Research	Spartanburg, SC	Principal investigator
US267	Angie	Williams		Spartanburg Medical Research	Spartanburg, SC	Study Coordinator
US267	Jami	Jones		Spartanburg Medical Research	Spartanburg, SC	Study Coordinator

US267	Charlie	Fogarty		Spartanburg Medical Research	Spartanburg, SC	Study Coordinator
US267	Nicole	Crockford	MD	Spartanburg Medical Research	Spartanburg, SC	Study team
US267	Kristen	Jones		Spartanburg Medical Research	Spartanburg, SC	Study team
US269	Frank	Eder	MD	Meridian Clinical Research	Binghamton, NY	Principal investigator
US269	Kelli	Quick		Meridian Clinical Research	Binghamton, NY	Study coordinator
US269	Abigail	Wine		Meridian Clinical Research	Binghamton, NY	Study coordinator
US269	Nicole	Croft		Meridian Clinical Research	Binghamton, NY	Study coordinator
US271	Marilou	Cruz	MD	Premier Health Research Center, LLC	Downey, California	Principal investigator
US271	Amiel	Guevarra		Premier Health Research Center, LLC	Downey, California	Study coordinator
US274	Joanna	Sexter	MD	Meridian Clinical Research	Washington, DC	Principal investigator
US274	Madison	Pfarr		Meridian Clinical Research	Washington, DC	Study team
US275	Kenneth	Steil	DO	Cognitive Clinical Trials	Phoenix, AZ	Principal investigator
US283	Brannon C	Perilloux	MD	Meridian Clinical Research	Baton Rouge, LA	Principal investigator
US283	Loney	Girod		Meridian Clinical Research	Baton Rouge, LA	Study coordinator
US283	Samantha	McMillon		Meridian Clinical Research	Baton Rouge, LA	Study coordinator
n/a	Jorge	Mejia-Galvis	MD	National Institutes of Health	Washington, DC	Medical monitor
n/a	Yuqing	Jiao		Fred Hutchinson Cancer Research Center	Seattle, WA	Statistician

Original Protocol Version 1.0

TITLE

A nasal swab study to assess the efficacy of vaccination in the prevention of SARS-CoV-2 infection among individuals enrolled in a Phase 3 efficacy trial of a SARS-CoV-2 recombinant spike protein (rS) vaccine with Matrix-M1™ (M1) adjuvant

DMID Protocol Number: 21-0011

DMID Funding Mechanism: 2UM1AI148689-02

IND Sponsor: NIAID

Lead Principal Investigator: Monica McArthur, MD, PhD

DMID Clinical Project Manager: Peter A. Wolff, MHA

Draft or Version Number: Draft v 1.0

Day Month Year
02 APRIL 2021

STATEMENT OF ASSURANCE

Each Institution will hold a current Federal Wide Assurance (FWA) issued by the Office of Human Research Protections (OHRP) for federally-funded human subjects research. Each FWA will designate at least one Institutional Review Board (IRB)/Independent Ethics Committee (IEC) registered with OHRP, for which the research will be reviewed and approved by the IRB/IEC and will be subject to continuing review [45 CFR 46.103(b)]. The IRB/IEC designated under an FWA may include an institution's IRB/IEC, an independent IRB/IEC, or an IRB/IEC of another institution after establishing a written agreement with that other institution.

STATEMENT OF COMPLIANCE

The study trial will be carried out in accordance with Good Clinical Practice (GCP) and as required by the following:

- United States Code of Federal Regulations (CFR) 45 CFR Part 46: Protection of Human Subjects
- Food and Drug Administration (FDA) Regulations, as applicable: 21 CFR Part 50 (Protection of Human Subjects), 21 CFR Part 54 (Financial Disclosure by Clinical Investigators), 21 CFR Part 56 (Institutional Review Boards), 21 CFR Part 11, and 21 CFR Part 312 (Investigational New Drug Application), 21 CFR 812 (Investigational Device Exemptions)
- International Conference on Harmonisation: Good Clinical Practice (ICH E6); 62 Federal Register 25691 (1997); and future revisions
- Belmont Report: Ethical Principles and Guidelines for the Protection of Human Subjects of Research, Report of the National Commission for the Protection of Human Subjects of Biomedical and Behavioral Research
- National Institutes of Health (NIH) Office of Extramural Research, Research Involving Human Subjects, as applicable
- National Institute of Allergy and Infectious Diseases (NIAID) Clinical Terms of Award, as applicable
- Applicable Federal, State, and Local Regulations and Guidance

SIGNATURE PAGE

The signature below provides the necessary assurance that this trial will be conducted according to all stipulations of the protocol, including all statements regarding confidentiality, and according to local legal and regulatory requirements and applicable US federal regulations and ICH E6 Good Clinical Practice (GCP) guidelines.

I agree to conduct the study in compliance with GCP and applicable regulatory requirements.

I agree to conduct the study in accordance with the current protocol and will not make changes to the protocol without obtaining the sponsor's approval and IRB/IEC approval, except when necessary to protect the safety, rights, or welfare of subjects.

Site Investigator Signature:*

Signed: ______________________________________ Date: __________________

Name

Title

TABLE OF CONTENTS

STATEMENT OF ASSURANCE 2
STATEMENT OF COMPLIANCE 3
SIGNATURE PAGE 4
TABLE OF CONTENTS 5
LIST OF FIGURES 8
LIST OF ABBREVIATIONS 9
PROTOCOL SUMMARY 12
1 KEY ROLES 14
2 BACKGROUND AND SCIENTIFIC RATIONALE 15
2.1 Background 15
2.2 Scientific Rationale 16
2.2.1 Purpose of Study 16
2.2.2 Study Population 16
2.3 Potential Risks and Benefits 16
2.3.1 Potential Risks 16
2.3.2 Potential Benefits 17
3 STUDY DESIGN, OBJECTIVES AND ENDPOINTS OR OUTCOME MEASURES 18
3.1 Study Design Description 18
3.2 Study Objectives 18
3.2.1 Primary 18
3.2.2 Secondary 18
3.2.3 Exploratory 19
3.3 Study Endpoints or Outcome Measures 19
3.3.1 Primary 19
3.3.2 Secondary 19
3.3.3 Exploratory 19

4 STUDY INTERVENTION/INVESTIGATIONAL PRODUCT 20
5 SELECTION OF SUBJECTS AND STUDY ENROLLMENT AND WITHDRAWAL....21
5.1.1 Subject Inclusion Criteria..21
5.1.2 Subject Exclusion Criteria 21
5.2 Withdrawal from the Study, Discontinuation of Study Product, or Study Termination 21
5.2.1 Withdrawal from the Study or Discontinuation of the Study Product...........21
5.2.2 Subject Replacement...22
5.2.3 Study Termination 22
6 STUDY PROCEDURES 23
6.1 Screening 23
6.2 Enrollment 23
6.3 Twice Weekly Swab Self-Collection at Home 24
6.4 MG Scanner Application 24
6.5 Final Study Visit 24
6.6 Protocol Deviations 25
7 DESCRIPTION OF CLINICAL AND LABORATORY EVALUATIONS 26
7.1 Laboratory Evaluations..26
7.1.1 Research Assays...26
8 ASSESSMENT OF SAFETY...27
8.1 Assessing and Recording Safety Parameters...27
8.1.1 Adverse Events (AEs) Reporting...27
8.2 Safety Oversight (ISM, SMC, DSMB, as applicable).................................27
9 HUMAN SUBJECTS PROTECTION 28
9.1 Institutional Review Board/Independent Ethics Committee 28
9.2 Informed Consent Process 29
9.2.1 Requirements for Permission by Parents/Guardians and Assent by Children (in case of a minor) 31

9.2.2 Other Informed Consent Procedures 31
9.3 Consent for Future Use of Stored Specimens and Data 32
9.4 Subject Confidentiality 32
9.5 Certificate of Confidentiality 32
9.6 Costs, Subject Compensation, and Research Related Injuries 33
10 STATISTICAL CONSIDERATIONS 34
10.1 Sample Size Considerations 34
10.2 Populations for Analysis 36
10.3 Analytic considerations 36
11 SOURCE DOCUMENTS AND ACCESS TO SOURCE DATA/DOCUMENTS 38
12 QUALITY CONTROL AND QUALITY ASSURANCE 39
13 DATA HANDLING AND RECORD KEEPING 40
13.1 Data Management Responsibilities 40
13.2 Data Coordinating Center/Biostatistician Responsibilities 40
13.3 Data Capture Methods 40
13.4 Types of Data 40
13.5 Study Records Retention 41
14 CLINICAL MONITORING 42
15 PUBLICATION POLICY 43
16 APPENDIX 44
Appendix A. Schedule of Events 44

LIST OF FIGURES

Figure 1 Power to detect vaccine efficacy against infection with 3000 participants, assuming follow-up for 6, 12 or 20 weeks and incidence rate equal to 2.9 per 100,000 person-weeks (left panel) and 5.7 per 100,000 person-weeks (right panel). 36

LIST OF ABBREVIATIONS

AE	Adverse Event/Adverse Experience
A/VMS	Asymptomatic/Very Mild Symptomatic Infection
BLA	Biologics License Applications
CFR	Code of Federal Regulations
CI	Confidence Interval
CIOMS	Council for International Organizations of Medical Sciences
CMS	Clinical Material Services
CONSORT	Consolidated Standards of Reporting Trials
COVID-19	Coronavirus Disease 2019
CoVPN	COVID-19 Prevention Network
CRF	Case Report Form
CRO	Contract Research Organization
CSR	Clinical Study Report
CVD	Center for Vaccine Development and Global Health
DCC	Data Coordinating Center
DHHS	Department of Health and Human Services
DMID	Division of Microbiology and Infectious Diseases, NIAID, NIH, DHHS
DSMB	Data and Safety Monitoring Board
eCRF	Electronic Case Report Form
FDA	Food and Drug Administration
FDAAA	Food and Drug Administration Amendments Act

FWA	Federal Wide Assurance
GCP	Good Clinical Practice
HIPAA	Health Insurance Portability and Accountability Act
IB	Investigator's Brochure
ICF	Informed Consent Form
ICH	International Conference on Harmonisation
ICMJE	International Committee of Medical Journal Editors
IDE	Investigational Device Exemption
IEC	Independent or Institutional Ethics Committee
IGS	Institute for Genome Sciences
IND	Investigational New Drug Application
IRB	Institutional Review Board
ISM	Independent Safety Monitor
JAMA	Journal of the American Medical Association
M1	Matrix-M1™ adjuvant
MedDRA®	Medical Dictionary for Regulatory Activities
MOP	Manual of Procedures
N	Number (typically refers to subjects)
NDA	New Drug Application
NEJM	New England Journal of Medicine
NIAID	National Institute of Allergy and Infectious Diseases, NIH, DHHS
NIH	National Institutes of Health
OHRP	Office for Human Research Protections

OHSR	Office for Human Subjects Research
PHI	Protected Health Information
PI	Principal Investigator
PREVENT-19	Pre-fusion Protein Subunit Vaccine Efficacy Novavax Trial COVID-19
QA	Quality Assurance
QC	Quality Control
rS	Recombinant spike protein of SARS-CoV-2
rS/M1	SARS-CoV-2 recombinant spike (rS) protein nanoparticle vaccine with M1 adjuvant
SAE	Serious Adverse Event/Serious Adverse Experience
SARS-CoV-2	Severe Acute Respiratory Syndrome Coronavirus 2
SMC	Safety Monitoring Committee
SOP	Standard Operating Procedure
UMSOM	University of Maryland School of Medicine
US	United States
VE	Vaccine Efficacy
WHO	World Health Organization

PROTOCOL SUMMARY

**Title:**	A nasal swab study to assess the efficacy of vaccination in the prevention of SARS-CoV-2 infection among individuals enrolled in a Phase 3 efficacy trial of a SARS-CoV-2 recombinant spike protein (rS) vaccine with Matrix-M1™ (M1) adjuvant
**Design of the Study:**	In this ancillary swab study, up to 3,000 pediatric participants 12-17 years of age, and up to 3,000 pediatric participants 6-11 years of age in the Phase 3 SARS-CoV-2 vaccine trial (PREVENT-19) sponsored by Novavax will be sampled twice weekly for approximately 6-20 weeks with nasal swabs for detection of SARS-CoV-2 infection by RT-PCR and incident infections tallied.
**Study Phase:**	Phase 3
**Study Population:**	Subset of participants in Protocol 2019nCoV-301, entitled "A Phase 3, Randomized, Observer-Blinded, Placebo-Controlled Study to Evaluate the Efficacy, Safety, and Immunogenicity of a SARS-CoV-2 Recombinant Spike Protein Nanoparticle Vaccine (SARS-CoV-2 rS) with Matrix-M1™ Adjuvant in Adult Participants ≥ 18 Years with a Pediatric Expansion to adolescents (12 to 17 years) and school-age children (6 to 11 years)" (PREVENT-19)
**Number of Sites:**	Up to 75 sites located in the US
**Description of Study Product or Intervention:**	Study participant will be randomized to receive either SARS-CoV-2 rS/M1 vaccine or placebo (2:1 allocation) on days 0 and 21 as part of the parent PREVENT-19 trial. In the current protocol, no interventional product will be administered.

**Study Objectives:**	Primary:<br>• To estimate the efficacy of SARS-CoV-2 rS/M1 vaccine against infection<br>Secondary:<br>• To estimate the efficacy of SARS-CoV-2 rS/M1 vaccine against asymptomatic/very mildly symptomatic infection<br>Exploratory:<br>• To determine the sequence of breakthrough SARS-CoV-2 infections<br>• To estimate the VE on duration of infection<br>• To estimate the VE against SARS-CoV-2 viral load as a proxy of transmission<br>• To estimate the efficacy of SARS-CoV-2 rS/M1 vaccine against a composite outcome of asymptomatic/ very mildly symptomatic infection plus pre-symptomatic infection<br>• To explore whether observed VE against COVID-19 illness reflects a transition from symptomatic into asymptomatic infections versus an absolute reduction in all SARS-CoV-2 infections
**Duration of Individual Subject Participation:**	Approximately 2-6 months for each participant
**Estimated Time to Last Subject/Last Study Day:**	Approximately 9 months from study activation to last subject/last study day

1 KEY ROLES

**Lead Principal Investigator:**	Monica McArthur, MD PhD, Assistant Professor of Pediatrics<br>Center for Vaccine Development and Global Health<br>University of Maryland School of Medicine
**DMID Clinical Project Manager:**	Peter A. Wolff, MHA, Clinical Project Manager<br>DMID, NIAID, NIH, DHHS
**Statistical and Data Coordinating Center:**	Fred Hutchinson Cancer Research Center, Statistical Center for HIV/AIDS Research and Prevention (SCHARP)

2 BACKGROUND AND SCIENTIFIC RATIONALE

2.1 Background

The primary goal of current COVID-19 vaccine trials is to measure vaccine efficacy (VE) against clinically significant infection. The aim is to save lives, reduce morbidity and sequelae, and alleviate the workload and financial burdens to the health care system. A hallmark of SARS-CoV-2 is its ability to efficiently replicate in the human host without or before causing symptoms. During these subclinical infections, individuals shed virus in the upper respiratory tract and may transmit SARS-CoV-2. Most trials do not directly assess vaccine efficacy against acquisition of virus overall as there is no routine sampling of participants (who do not meet endpoint definitions for illness) to detect SARS-CoV-2. Asymptomatic seroconversion to viral antigens not contained in the vaccine is often used as a surrogate, but the precision of these estimates is subject to variability in the sensitivity and specificity of the serologic assays and the durability of antibody following subclinical infection and may not predict viral shedding. Yet the vaccine effect on infection is important to understand, both for its potential impact on transmission and to better characterize the vaccine effect on individuals.

On a population level, vaccine efficacy against all infection, including asymptomatic or very mildly symptomatic infection, is an important endpoint. The impact of a pandemic virus's ability to spread sub-clinically permeates the educational systems, essential infrastructure and operations, and the social, psychological, financial, and political fabric of our society. For policy decision-makers, knowledge of vaccine efficacy against asymptomatic or very mildly symptomatic infection is critical for vaccine allocation and distribution, prioritization of groups to receive vaccine, re-opening and community mitigation strategies, and determining the need for continued individual infection control measures such as social distancing and face coverings. To address these knowledge gaps, we will conduct an ancillary swab study to estimate vaccine efficacy against acquisition of SARS CoV-2 infection, regardless of symptomatology.

This ancillary swab study will leverage the ongoing placebo-controlled Phase 3 SARS-CoV-2 vaccine trial (PREVENT-19 NCT04611802) pediatric expansion study sponsored by Novavax (Gaithersburg, MD) that includes 3,000 adolescents (12-17 years of age) and 3,000 school age children (6-11 years of age). The primary aim of the pediatric expansion study of PREVENT-19 is to assess the safety and effectiveness of a SARS-CoV-2 recombinant spike (rS) protein

nanoparticle vaccine administered with the Matrix-M1™ adjuvant (herein designated rS/M1 vaccine). Participants in the pediatric expansion are randomized to receive either SARS-CoV-2 rS/M1 vaccine or placebo (2:1 allocation) on days 0 and 21.

2.2 Scientific Rationale

2.2.1 Purpose of Study

The primary question that will be addressed in this ancillary study is whether SARS CoV-2 vaccines can prevent overall acquisition of infection and, therefore, have the ability to interrupt transmission. However, it is likely that some vaccinated individuals will continue to shed virus asymptomatically. Therefore, vaccine efficacy against the magnitude and duration of shedding will be included as exploratory aims, as these parameters can be considered to be proxies of reduced transmission potential, recognizing that definitive results require direct measurement of the frequency of infection in contacts of vaccinated compared to unvaccinated individuals.

2.2.2 Study Population

The pediatric expansion to the Phase 3 PREVENT-19 trial sponsored by Novavax is estimated to start enrollment in April-May 2021. The expansion will enroll ~3,000 adolescents (12-17 years of age) and ~3,000 school-aged children (6-11 years of age). All children in each age cohort who are participating in the Phase 3 pediatric expansion trial are eligible for enrollment in the ancillary swab study. Approximately 75 sites are expected to participate in the pediatric expansion.

2.3 Potential Risks and Benefits

2.3.1 Potential Risks

Risks associated with this ancillary study are primarily associated with potential loss of confidentiality. The study team will make every effort to protect each participant's privacy and confidentiality. Participants will be assigned a unique study number (Subject ID) for all data and sample collection. Paper data collection forms and specimen labels will be identified only with Subject ID. Study files will be stored in locked file cabinets and electronic data collection and storage tools will be password protected. Access to participant data will be limited to authorized study personnel.

This study presents no greater than minimal risk to the participants. Risks associated with mid-turbinate nasal swab collection may include sneezing, eye watering, coughing, or possible epistaxis.

Notably, the ancillary swab study will be performed at the convenience of the Phase 3 study, where the goals of the Phase 3 study will take priority over this ancillary study (i.e., consideration for the Phase 3 study objectives and endpoints will supersede those of the ancillary study). The ancillary study is designed to generate additional valuable data on efficacy of SARS-CoV-2 rS/M1 vaccine against infection, including asymptomatic/very mildly symptomatic infection.

2.3.2 Potential Benefits

There are no direct benefits to participants participating in this ancillary study; however, the results of this ancillary study may provide valuable information that can help others in the future.

3 STUDY DESIGN, OBJECTIVES AND ENDPOINTS OR OUTCOME MEASURES

3.1 Study Design Description

In this ancillary swab study, up to 3,000 participants in each age cohort who are participating in the PREVENT-19 Pediatric Expansion study and who provide informed consent/assent for the ancillary swab study will be sampled twice weekly starting around the day 21 visit, or thereafter, of the initial injection period until the blinded cross-over period (~20 weeks) with nasal swabs for detection of SARS-CoV-2 infection by RT-PCR and incident infections tallied. Data sources from the Phase 3 trial (e.g., study product assignment, daily symptomatology) will be used in the ancillary study to estimate the vaccine efficacy against SARS-CoV-2 infection, including asymptomatic infection or mildly symptomatic infection not meeting the COVID-19 primary endpoint definition for the Phase 3 study. Longer infections with higher viral loads, as a proxy for transmission potential, will be captured in this design.

Participants in the PREVENT-19 Phase 3 trial (pediatric expansion study) are randomized to receive either SARS-CoV-2 rS/M1 vaccine or placebo (2:1 allocation) on days 0 and 21. If satisfactory vaccine efficacy and safety are demonstrated for SARS-CoV-2 rS/M1 in an analysis of the primary endpoint, participants in the adult main study will be administered two injections of the alternate study vaccine or placebo 21 days apart ("blinded cross-over period"). Novavax plans to apply to FDA for Emergency Use Authorization (EUA) at this time. The pediatric expansion study will have a "blinded-crossover" after ~6 months of follow-up after the completion of the initial set of vaccinations. Pediatric participants will be scheduled for administration of 2 injections of the alternate study material 21 days apart.

3.2 Study Objectives

3.2.1 Primary

- To estimate the efficacy of SARS-CoV-2 rS/M1 vaccine against infection

3.2.2 Secondary

- To estimate the efficacy of SARS-CoV-2 rS/M1 vaccine against asymptomatic/ very mildly symptomatic infection

3.2.3 Exploratory

- To determine the sequence of breakthrough SARS-CoV-2 infections
- To estimate the VE on duration of infection
- To estimate the VE against SARS-CoV-2 viral load as a proxy of transmission
- To estimate the efficacy of SARS-CoV-2 rS/M1 vaccine against a composite outcome of asymptomatic/ very mildly symptomatic infection plus pre-symptomatic infection
- To explore whether observed VE against COVID-19 illness reflects a transition from symptomatic into asymptomatic infections versus an absolute reduction in all SARS-CoV-2 infections

3.3 Study Endpoints or Outcome Measures

3.3.1 Primary

- Reverse transcriptase polymerase chain reaction (RT-PCR)-confirmed SARS-CoV-2 infection detected in nasal swabs collected twice weekly from the day 21 visit or thereafter, until ancillary study end in the vaccine group and the placebo group.

3.3.2 Secondary

- RT-PCR-confirmed SARS-CoV-2 infection detected in nasal swabs collected twice weekly from the day 21 visit or thereafter, until ancillary study end, without COVID-19 illness as defined in the primary Phase 3 study endpoint

3.3.3 Exploratory

- Viral genome sequence of PCR-positive samples
- Duration of shedding as indicated by RT-PCR in vaccinated vs placebo recipients
- Reduction in viral load (and perhaps subgenomic RNA), as a proxy for transmission, in PCR-positive samples from vaccinated vs placebo recipients
- Proportion of days that are symptom-free on days that positive swabs are collected
- Ratio of asymptomatic to symptomatic infections in vaccinated vs placebo recipients. Data on symptomatology will be extracted from the Phase 3 trial database, which will be separate but linkable to the ancillary study database.

4 STUDY INTERVENTION/INVESTIGATIONAL PRODUCT

Study participant will be randomized to receive either SARS-CoV-2 rS/M1 vaccine or placebo (2:1 allocation) on days 0 and 21 as part of the parent PREVENT-19 trial. In the current protocol, no interventional product will be administered. Each participant's blinded allocation will be accessed through the PREVENT-19 trial database during analysis.

5 SELECTION OF SUBJECTS AND STUDY ENROLLMENT AND WITHDRAWAL

Subject Inclusion and Exclusion Criteria must be confirmed by a study clinician licensed to make medical diagnoses.

No exemptions will be granted on Subject Inclusion/Exclusion Criteria.

Eligibility Criteria

5.1.1 Subject Inclusion Criteria

Each participant must meet all of the following criteria to be enrolled in this study:

1. Participant in the parent study
2. Less than 18 years and ≥ 6 years of age at time of enrollment
3. Ability and willingness to give informed consent/assent prior to study enrollment and comply with the study procedures
4. Received two doses of study product
5. Providing surveillance data on COVID-19 symptoms as described in the parent protocol
6. Access to a cell phone with ability to download and use the MG ScannerTM Application
7. Active email address

5.1.2 Subject Exclusion Criteria

Participants meeting the following criterion will be excluded from the study:

1. Any condition that would, in the opinion of the investigator, place the participant at an unacceptable risk of injury or render him/her unable to comply with the study requirements.

5.2 Withdrawal from the Study, Discontinuation of Study Product, or Study Termination

5.2.1 Withdrawal from the Study or Discontinuation of the Study Product

Subjects may voluntarily withdraw their consent for study participation at any time without penalty or loss of benefits to which they are otherwise entitled.

An investigator may also withdraw a subject from participating in the study for any reason. If a subject withdraws or is withdrawn prior to completion of the study, the reason for this decision must be recorded in the case report forms (CRFs).

The reasons, might include, but are not limited to the following:

- Subject no longer meets eligibility criteria
- Subject becomes noncompliant
- Medical disease or condition, or new clinical finding(s) for which continued participation, in the opinion of the investigator might compromise the safety of the subject, interfere with the subject's successful completion of this study, or interfere with the evaluation of responses
- Subject lost to follow-up
- Subject unblinded in parent study

The investigator will inform the participant that already collected data will be retained and analyzed even if the participant withdraws from this study.

5.2.2 Subject Replacement

Subjects who withdraw, or are withdrawn from this study, or are lost to follow-up after signing the informed consent form (ICF) will not be replaced.

5.2.3 Study Termination

If the study is prematurely terminated by the sponsor, any regulatory authority, or the investigator for any reason, the investigator will promptly inform the study subjects and assure appropriate therapy or follow-up for the subjects, as necessary. The investigator will provide a detailed written explanation of the termination to the IRB/IEC.

6 STUDY PROCEDURES

6.1 Screening

Participants in the Phase 3 trial will have the ancillary study explained to them by study personnel during their day 21 visit or at another study encounter. If contact at the day 21 visit is not possible, a later appointment (either in-person or by tele-health) may be arranged for enrollment into the ancillary swab study. Those with expressed interest will provide informed, consent/assent prior to any study procedures. The consent form will include permission for investigators to review the participant's research records and to link with the parent study database at the appropriate time to obtain related information on randomization assignment, COVID-19 symptomatology, and RT-PCR results from study-specific swabs or other relevant events.

Informed consent/assent is required prior to enrollment and prior to any study-related procedures. Parental or guardian permission will be obtained for all children. Assent will be obtained from children according to the IRB requirements at each site. To ensure that the participant's rights are protected and that all study participants receive, read, have explanation of and sign an informed consent/assent prior to enrolling in this study, research team members will follow the informed consent process outlined in site-specific SOPs and as approved by the single IRB, as applicable.

Following the attainment of informed consent/assent, eligibility criteria will be reviewed. The screening visit will occur concurrently with enrollment in most cases. A window of 7 days between screening and enrollment is allowed.

6.2 Enrollment

After obtaining informed consent/assent as above, study staff will collect a limited array of demographic and clinical information from the participant. An application will be downloaded onto the participant's or participant's parent's smart phone that will permit the participant to scan the bar code on nasal swab vials at home prior to sending them to the ancillary swab study central laboratory at the University of Maryland School of Medicine (UMSOM)/Institute for Genome Sciences (IGS) for SARS CoV-2 RT-PCR testing and storage. The application will log the date/time of each sample collection during the home swabbing period. No Protected Health Information (PHI) will be transferred through the application. Study personnel will instruct the

participant on the methods for self-collection and shipment of nasal swab specimens. Participants will receive a packet of swab kits for their home use.

6.3 Twice Weekly Swab Self-Collection at Home

Beginning the week after day 21, or soon thereafter, (week 1 in Appendix A), the participant will self-collect a mid-turbinate nasal swab for SARS CoV-2 RT-PCR testing twice weekly. If the child is not able to self-collect independently, collection may be performed by the parent or by the child under parental supervision. For example, each participant will collect a swab on the Monday (+1) and Thursday (+1) of each week. The duration of nasal swabbing for each participant cannot be determined with precision at this time; it might range from approximately 6-20 weeks depending on when the participant is enrolled and when the cross-over study begins. Home nasal swabs will be retrieved by a courier and/or mail delivery service and delivered to the UMSOM/IGS laboratory.

6.4 MG Scanner Application

The MG Scanner application is an internally developed mobile application for iOS and Android devices. The application is designed to facilitate the registration of participants for COVID-19 testing through indirect interaction with the Zoran system. MG scanner also provides functionality for the scanning and registration of collected samples for testing.

In this system the user registers his/her device through the application via an emailed invitation. This registration binds the device ID to the participant’s medical record number (MRN). The MRN is unique to each individual and an individual only has one MRN in the Zoran system. This MRN is separate from the participant’s hospital/clinic MRN or the parent study ID. The user can then submit a sample for testing through a sample scanning component of the app. The app will scan two globally unique IDs from pre-labeled sample collection tubes and those IDs will be bound to the participant MRN as a pending specimen. Scanning of two globally unique IDs ensures the tube only occurs once and is positively associated with the participant MRN. If one barcode is damaged during the collection or transport process the specimen can still be positively associated with the submitting participant.

6.5 Final Study Visit

The final ancillary study swab will be collected at the time of the cross-over phase in the Parent Protocol at which point the participant’s ancillary study participation will be completed

(Appendix). The blinded cross-over study for the pediatric expansion is expected to begin approximately 3-6 months after the day 21 visit.

6.6 Protocol Deviations

A protocol deviation is any noncompliance with the clinical trial protocol, GCP, or protocol-specific Manual of Procedures (MOP) requirements. Failure of a participant to collect nasal swabs at home or collection outside of the window will not be captured as protocol deviations as it will not impact study safety or overall scientific integrity. The noncompliance may be either on the part of the subject, the investigator, or the study site staff. As a result of deviations, corrective actions should be developed by the site and implemented promptly. It is the responsibility of the site Principal Investigator and other study personnel to use continuous vigilance to identify and report protocol deviations. All individual protocol deviations will be addressed in subject study records. All protocol deviations, either individual, product, or site-specific will be collected and the record stored in a sponsor-determined location. Protocol deviations must be sent to the local IRB/IEC per its guidelines. The site Principal Investigator and other study personnel are responsible for knowing and adhering to their IRB/IEC requirements.

7 DESCRIPTION OF CLINICAL AND LABORATORY EVALUATIONS

7.1 Laboratory Evaluations

7.1.1 Research Assays

At the enrollment visit (or soon after that if enrolled by video), each participant will be provided kits for collection containing tubes and swabs along with collection instructions in each kit. They will be instructed on the proper method of obtaining nasal swabs during the visit (in-person or tele-health). Additionally, the participants will be shown how to register with the application (MG Scanner^{TM}) to log each sample when it is collected.

At each required timepoint, the participant or his/her parent will collect a mid-turbinate nasal specimen of nasal secretions by swabbing the mid-turbinate of each nostril according to detailed standardized procedures demonstrated in the provided instructions. The swab will be placed in a vial containing buffer solution and maintained at room temperature.

The application will log the date/time of each sample collection during the observation period.

The swabs will be sent to the University of Maryland School of Medicine, Institute of Genome Sciences (UMSOM/IGS) laboratory for analyses.

7.1.1.1 Laboratory Specimen Preparation, Handling, and Storage

Samples will be shipped to UMSOM/IGS and will be run as they are received. Stability studies have demonstrated that all sample storage and shipping can be done at room temperature without affecting the SARS-CoV-2 RT-PCR test performance.

7.1.1.2 Laboratory Specimen Shipping

Participants will package collected swabs according to the provided instructions in provided shipping envelopes or boxes addressed to the UMSOM/IGS laboratory. Shipping to the UMSOM/IGS laboratory will occur every 1-2 weeks.

8 ASSESSMENT OF SAFETY

8.1 Assessing and Recording Safety Parameters

.

8.1.1 Adverse Events (AEs) Reporting

No adverse reactions are anticipated in this study, and the study procedures present no anticipated risks beyond the completion of the study. It is anticipated that the participants may experience discomfort when mid-turbinate nasal swab specimens are being collected; however, the risks and discomfort in this study are not greater than those ordinarily encountered during the routine collection of the mid-turbinate nasal swabs at a clinician's office.

Vaccine related AEs will not be collected as part of this ancillary study. The Sponsor will collect AE information via the CRF. AEs that last greater than 15 minutes will be collected from the time of swab collection to the time that the subject completes the study. Such data collection will include the start and stop dates of the event, causality with the swab collection (not related, related), outcome (ongoing, recovered, recovered with sequelae, not recovered, fatal), code for seriousness (SAE, not serious), and intensity (mild, moderate, or severe). The definitions of mild, moderate, or severe are as follows:

1 = Mild (awareness of a symptom but the symptom is easily tolerated)

2 = Moderate (discomfort enough to cause interference with usual activity)

3 = Severe (incapacitating; unable to perform usual activities; requires absenteeism or bed rest)

8.2 Safety Oversight (ISM, SMC, DSMB, as applicable)

A Safety oversight Committee is not required for this minimum risk study. Safety oversight will be the responsibility of the Principal Investigator

9 HUMAN SUBJECTS PROTECTION

9.1 Institutional Review Board/Independent Ethics Committee

Each site principal investigator will obtain IRB approval for this protocol to be conducted at his/her research site(s) and send supporting documentation to the DMID before initiating recruitment of subjects. The investigator will submit applicable information to the IRB/IEC on which it relies for the review, to conduct the review in accordance with 45 CFR 46, ICH E6 GCP, and as applicable, 21 CFR 56 (Institutional Review Boards) and 21 CFR 50 (Protection of Human Subjects), other federal, state, and local regulations. The IRB/IEC must be registered with OHRP as applicable to the research. DMID must receive the documentation that verifies IRB/IEC-approval for this protocol, associated informed consent documents, and upon request any recruitment material and handouts or surveys intended for the subjects, prior to the recruitment and enrollment of subjects.

Any amendments to the protocol or consent materials will be approved by the IRB/IEC before they are implemented. IRB/IEC review and approval will occur at least annually throughout the enrollment and follow-up of subjects and may cease if annual review is no longer required by applicable regulations and the IRB/IEC. The investigator will notify the IRB/IEC of deviations from the protocol and reportable SAEs, as applicable to the IRB/IEC policy.

Each institution engaged in this research will hold a current Federalwide Assurance (FWA) issued by the Office of Human Research Protection (OHRP) for federally funded research.

A single IRB of record, WCG IRB, will be accountable for compliance with regulatory requirements for this multi-centered study, at participating sites. Written reliance agreements between the single IRB and participating sites will be required. The reliance agreements will set forth the specific responsibilities of the IRB and each participating site. Participating sites will then rely on the IRB of record to satisfy the regulatory requirements relevant to the IRB review. The participating sites will maintain essential required documentation of IRB reviews, approvals, and correspondence, and must provide copies of any agreements and essential documentation to the DMID or regulatory authorities upon request.

The IRB/IEC will determine that adequate provisions are made for soliciting the permission of each child's parent(s) or legal guardian(s), including whether permission of one parent/guardian is sufficient for research or whether permission is to be obtained from both parents/guardians.

The IRB/IEC will determine how consent from subjects will be obtained when participation in the study is ongoing, and the subject has reached the age of majority.

9.2 Informed Consent Process

Informed consent is a process that is initiated prior to an individual agreeing to participate in a trial and continuing throughout the individual's trial participation. Before any study procedures are performed, informed consent will be obtained and documented. Subjects will receive a concise and focused presentation of key information about the clinical trial, verbally and with a written consent form. The explanation will be organized and presented in lay terminology and language that facilitates understanding why one might or might not want to participate.

An investigator or designee will describe the protocol to potential subjects' parent(s)/guardian(s). The key information about the purpose of the study, the procedures and experimental aspects of the study, risks and discomforts, any expected benefits to the subject, and alternative treatment will be presented first to the subject's parent(s)/guardian(s).

Subjects' parent(s)/guardian(s) will also receive an explanation that the trial involves research, and a detailed summary of the proposed study procedures. This will include aspects of the trial that are experimental, any expected benefits, all possible risks, and the expected duration of the subject's participation in the trial.

Subjects' parent(s)/guardian(s) will be informed that they will be notified in a timely manner if information becomes available that may be relevant to their willingness to continue participation in the trial. Subjects will receive an explanation as to whether any compensation and any medical treatments are available if injury occurs, and, if so, what they consist of, or where further information may be obtained. Subjects' parent(s)/guardian(s) will be informed of the anticipated financial expenses, if any, to the subject's parent(s)/guardian(s) for their child's participation in the trial, as well as any anticipated prorated payments, if any, to the subject and his/her parent(s)/guardian(s) for participation in the trial. They will be informed of whom to contact (e.g., the investigator) for answers to any questions relating to the research project.

Information will also include the foreseeable circumstances and/or reasons under which the subject's participation in the trial may be terminated. The subjects' parent(s)/guardian(s) will be informed that participation is voluntary and that they are free to withdraw from the study for any reason at any time without penalty or loss of benefits to which the subject and his/her parent(s)/guardian(s) are otherwise entitled.

The extent of the confidentiality of the subjects' records will be defined, and subjects' parent(s)/guardian(s) will be informed that applicable data protection legislation will be followed. Subjects will be informed that the monitor(s), auditors(s), IRB, NIAID, and regulatory authority(ies) will be granted direct access to the subject's original medical records for verification of clinical trial procedures and/or data without violating the confidentiality of the subject, to the extent permitted by the applicable laws and regulations, and that, by signing a written informed consent form, the subject's parent(s)/guardian(s) is/are authorizing such access.

Subjects' parent(s)/guardian(s) will be informed that records identifying the subject will be kept confidential, and, to the extent permitted by the applicable laws and/or regulations, will not be made publicly available and, if the results of the trial are published, the subject's identity will remain confidential. Subjects' parent(s)/guardian(s) will be informed whether private information collected from this research and/or specimens will be used for additional research, even if identifiers are removed.

Subjects' parent(s)/guardian(s) will be allowed sufficient time to consider participation in this research trial and have the opportunity to discuss this trial with their family, friends or legally authorized representative, or think about it prior to agreeing to participate.

Informed consent forms will be IRB-approved, and subjects' parent(s)/guardian(s) will be asked to read and review the consent form. Subjects' parent(s)/guardian(s) must sign the informed consent form prior to starting any study procedures being done specifically for this trial.

Once signed, a copy of the informed consent form will be given to the subjects' parent(s)/guardian(s) for their records. The subjects' parent(s)/guardian(s) may withdraw consent at any time throughout the course of the trial. The rights and welfare of the subject(s) will be protected by emphasizing that the quality of their medical care will not be adversely affected if the parent(s)/guardian(s) decline to allow their child to participate in this study.

Study personnel may employ recruitment efforts prior to obtaining study consent if a patient-specific screening consent is on record or if the IRB has agreed that chart review is allowed without a fully executed screening consent.

New information will be communicated by the site principal investigator or designee to subjects' parent(s)/guardian(s) who consent for their child to participate in this trial in accordance with IRB requirements. The informed consent document will be updated, and subjects' parent(s)/guardian(s) will be re-consented per IRB requirements, if necessary. Subjects' parent(s)/guardian(s) will be given a copy of all informed consent forms that they sign.

9.2.1 Requirements for Permission by Parents/Guardians and Assent by Children (in case of a minor)

Investigators will follow IRB/IEC requirements for enrollment of minors in this study. Minors will be informed about the study to the extent understandable to the minor. Investigators or designee will conduct the consent process with the parent(s)/legal guardian(s), who will be given an IRB/IEC-approved permission form, which may be referred to as a consent form, to read, review, and sign prior to any study procedures. The parent(s)/legal guardian(s) will be provided meaningful study information including a statement that this study involves research, the child may not benefit from the trial, and the study involves risk. The required elements will be clearly presented, including the purpose of the study, the experimental procedures, the potential risks and discomforts, known adverse effects, possible benefits of the study for the subject, use and disclosure of private information, and other elements that are part of obtaining proper consent. The subject's parent(s)/legal guardian(s) will be allowed sufficient time to discuss questions with the investigator or designee.

The investigator or designee will describe in simplified terms the details of the study procedures, risks and discomforts, benefits, and other consent elements, as appropriate. A separate IRB/IEC-approved assent form will be used for the minor (as appropriate), who may read and sign the form, or have it read to him/her prior to participation in study procedures. Assent may be obtained verbally or waived when approved by the IRB/IEC as appropriate to age. If a child declines to participate in the trial when assent is required by the IRB/IEC, the subject will not be enrolled even though the parent(s)/guardian(s) have provided permission.

To ensure that consent is an ongoing process throughout the subject's participation in the study, the investigator and staff will review information as needed with the subject (as appropriate) and the parent(s)/legal guardian(s) and confirm that assent and permission are continuing. The permission and assent documents will be updated when new information is acquired that may impact the decision to continue in the study, and the subject's assent and the parent(s)/legal guardian's permission will be obtained, as applicable.

The subject who reaches the age of majority will be consented at the next visit prior to study procedures. When no further visits are planned but the subject's participation is ongoing, the consent will be obtained via IRB/IEC-approved processes.

9.2.2 Other Informed Consent Procedures

Illiterate Subjects/Parent(s)/Guardian(s)

If subjects' parent(s)/guardian(s) are illiterate**,** they will not be allowed to participate in the study.

9.3 Consent for Future Use of Stored Specimens and Data

Residual samples/specimens are those that are left over after protocol-specified testing and this study has been completed. Subjects' parent(s)/guardian(s) will be asked for permission to keep any remaining (residual) specimens (extracted viral RNA) derived from nasal swab samples for possible use in future research studies, such as examining additional virologic assessments. These residual specimens will be stored, coded, indefinitely at UMSOM/IGS. The recipients of specimens will be informed that these specimens have a NIH certificate of confidentiality. The information provided to a recipient will not contain direct identifiable information. The research will not include human whole genome sequencing, nor will your child's nasal swabs be used to generate a cell line for genetic testing.

9.4 Subject Confidentiality

Subject confidentiality is strictly held in trust by the participating investigators, their staff, and the sponsor(s) and their agents. This confidentiality includes documentation, investigation data, subject's clinical information, and all other information generated during participation in the study. No information concerning the study, or the data generated from the study, will be released to any unauthorized third party without prior written approval of the DMID and the subject's parent(s)/guardian(s). Subject confidentiality will be maintained when study results are published or discussed in conferences. The study monitor, or other authorized representatives of the sponsor or governmental regulatory agencies, may inspect all documents and records required to be maintained by the investigator, including but not limited to, medical records (office, clinic, or hospital) and pharmacy records for the subjects in this study. The clinical study site will permit access to such records.

All records will be kept locked and all computer entry and networking programs will be carried out with coded numbers only and with password-protected systems. All non-clinical specimens, evaluation forms, reports, and other records that leave the site will be identified only by a coded number.

9.5 Certificate of Confidentiality

To protect privacy, we have received a Certificate of Confidentiality. With this Certificate, the researchers cannot be forced to release information that may identify the research subject, even by a court subpoena, in any federal, state, or local civil, criminal, administrative, legislative, or other proceedings. The researchers will use the Certificate to resist any demands for information

that would identify the subject, except as explained below.

The Certificate cannot be used to resist a demand for information from personnel of the United States Government that is used for auditing or evaluation of federally funded projects, like this study, or for information that must be released in order to meet the requirements of the Federal Food and Drug Administration (FDA).

A Certificate of Confidentiality does not prevent the subject from voluntarily releasing information about themselves or their involvement in this research. If any person or agency obtains a written consent to receive research information, then the researchers may not use the Certificate to withhold that information.

The Certificate of Confidentiality does not prevent the researchers from reporting without the subject's parent(s)/guardian(s) consent, information that would identify the subject as a participant in the research project regarding matters that must be legally reported including child and elder abuse, sexual abuse, or wanting to harm themselves or others.

The release of individual private information or specimens for other research will only occur if consent was obtained from the individual's parent(s)/guardian(s) for the individual to whom the information, document, or biospecimen pertains, or for the purposes of other research that is in compliance with applicable Federal regulations governing the protection of human subjects in research.

9.6 Costs, Compensation, and Research Related Injuries

There is no cost to subjects or their parent(s)/guardian(s) for the research tests while taking part in this trial. Subjects and their parent(s)/guardian(s) may be compensated for their participation in this trial. Compensation will be in accordance with the local IRB's policies and procedures, and subject to IRB approval.

10 STATISTICAL CONSIDERATIONS

This study will enroll from participants enrolled in the main study. Based on enrollment targets for the phase 3 trial, up to 6,000 participants may be enrolled, maintaining the 2:1 (vaccine:placebo) randomization from the phase 3 study.

10.1 Sample Size Considerations

Very little data are available on incidence rates of SARS-CoV-2 infection in children age 6-17. The CDC (https://www.cdc.gov/coronavirus/2019-ncov/cases-updates/burden.html) estimates a cumulative incidence in children 5-17 years old of 27,218 per 100,000 from February 2020-December 2021 which translates into approximately 5.7 per 100,000 person-weeks. A similar incidence estimate can be calculated based on prevalence in children presenting for non-COVID-19 related hospital visits in the summer of 2020 (Sola et al, JAMA Peds 2020). When estimating power, we conservatively estimate that only 3,000 children enroll and complete follow-up with twice per week swabbing. The Figure below provides the power (with two-sided alpha = 0.05) to reject the null hypothesis that VE=0 under various scenarios: VEs of 0.4, 0.6 and 0.8 and attack rates equal to or 0.5 to 2 times that observed in 2020. For example, If the attack is equal to 5.7 per 100,000 person-weeks with 6 weeks of follow-up, there is >90% power to detect a VE of 60%. If the attack rate half that from 2020 with 6 weeks of follow-up, then the study has >80% power to detect VE of 70%. These calculations assume all infections will be detected with twice per week swabbing.

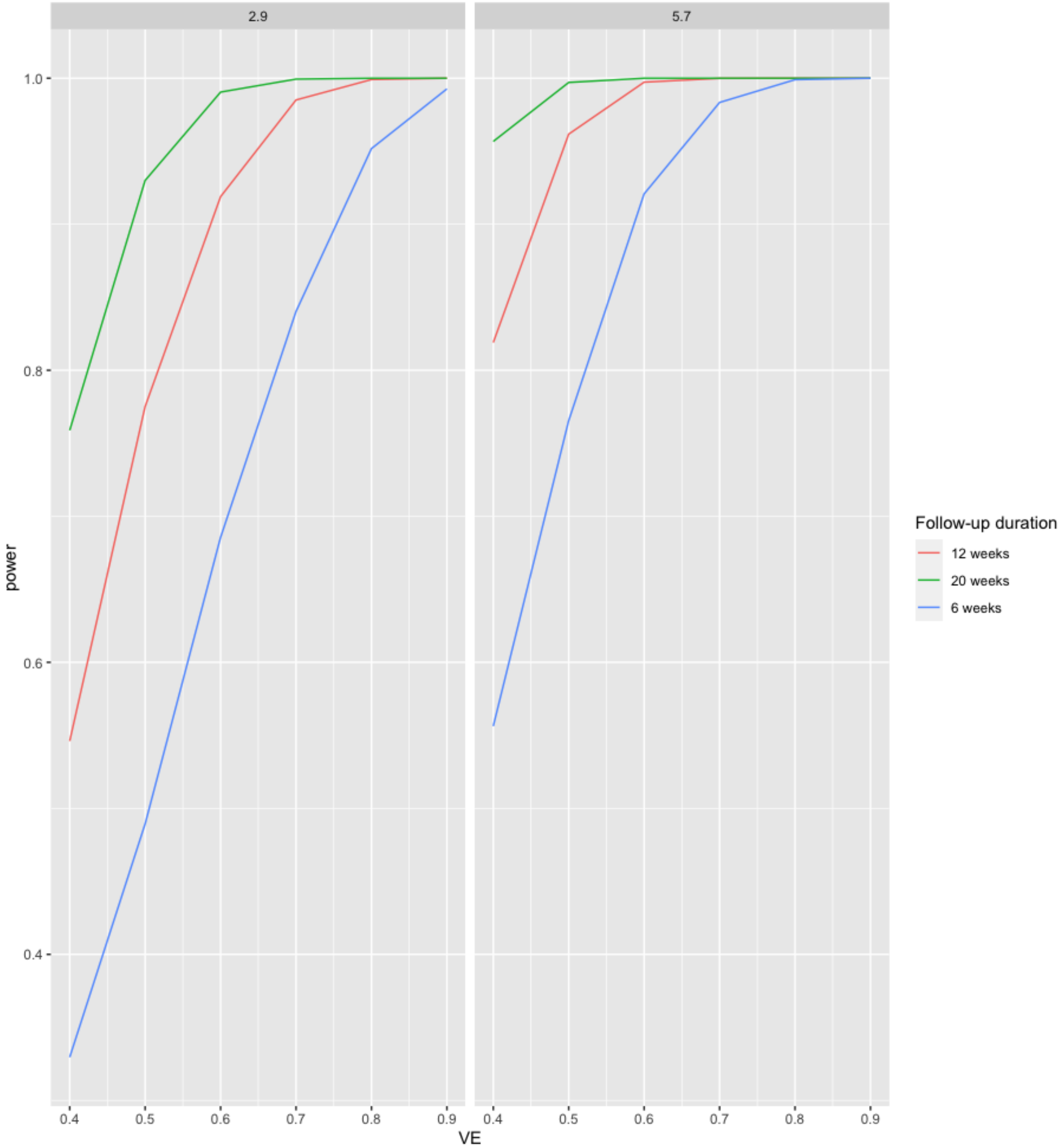
2.9
5.7
power
VE
1.0
0.8
0.6
0.4
0.4
0.5
0.6
0.7
0.8
0.9
Follow-up duration
12 weeks
20 weeks
6 weeks

Figure 1 Power to detect vaccine efficacy against infection with 3000 participants, assuming follow-up for 6, 12 or 20 weeks and incidence rate equal to 2.9 per 100,000 person-weeks (left panel) and 5.7 per 100,000 person-weeks (right panel).

10.2 Populations for Analysis

For analysis purposes, the following populations are defined:

Intention to Treat: All enrolled participants

Incident Infections: All participants with SARS-CoV-2 RNA detected by RNA-RT-PCR

More populations may be defined in the SAP as appropriate.

10.3 Analytic considerations

All tests will be conducted under a two-sided alpha to reject a null hypothesis that VE=0.

Primary Objective: Prevention of Infection

Endpoint: SARS-CoV-2 detected by RT-PCR on self-collected nasal swabs

Population: Intention to Treat

Incidence of SARS-CoV-2 infection will be analyzed using a Cox Proportional Hazards model with an outcome of time to first positive swab. Censoring will occur at the last negative swab for participants who never have a positive swab. The Statistical Analysis Plan will include further details including plans in the event of non-proportional hazards, differential loss to follow-up and adustment. Randomization arm will be merged from the primary study database.

Secondary Objective: Prevention of Asymptomatic/Very Mild Symptomatic Infection (A/VMS)

Endpoint: SARS-CoV-2 detected by RT-PCR on self-collected nasal swabs absent COVID-defining symptoms compatible with primary endpoint

Population: Intention to Treat

Incident infections that do not meet the COVID-19 illness endpoint definition (within a pre-defined number of days) will count towards the endpoint of A/VMS infection. Vaccine efficacy will be calculated as 1-[(number of endpoints in vaccine arm)/(number in vaccine arm)]/[(number of endpoints in placebo arm)/(number in placebo arm)]. If rates of censoring are high, either due to drop out or early cross-over, this analysis will instead be conducted using time to event analyses, such as Cox Proportional Hazards models. Details will be contained in the Statistical Analysis Plan. The analysis will be conducted on the Intention to Treat population. Data sources from the Phase 3 trial (e.g., study product assignment and clinical symptomatology, including adjudicated COVID-19 illness endpoints) will be accessed to ascertain the symptomatology of participants who are PCR-positive in the ancillary study.

Exploratory analyses conducted on the Intention to Treat and Incident Infections populations to meet the exploratory objectives of this protocol will be detailed in the Statistical Analysis Plan.

11 SOURCE DOCUMENTS AND ACCESS TO SOURCE DATA/DOCUMENTS

Each participating site will maintain appropriate medical and research records in compliance with ICH E6, Section 4.9 and regulatory and institutional requirements for the protection of confidentiality of subjects. Each site will permit authorized representatives of the DMID, its designees, and appropriate regulatory agencies to examine (and when required by applicable law, to copy) clinical records for the purposes of quality assurance reviews, audits, and evaluation of the study safety and progress. These representatives will be permitted access to all source data and source documents, which include, but are not limited to, hospital records, clinical and office charts, laboratory notes, memoranda, subjects' memory aid or evaluation checklists, pharmacy dispensing records, recorded data from automated instruments, copies or transcriptions certified after verification as being accurate and complete, microfiches, photographic negatives, microfilm or magnetic media, x-rays, and subject files and records kept at the pharmacy, at the laboratories, and medico-technical departments involved in the clinical trial.

12 QUALITY CONTROL AND QUALITY ASSURANCE

Each participating site(s) and its subcontractors are responsible for conducting routine quality assurance (QA) and quality control (QC) activities to internally monitor study progress and protocol compliance. The site principal investigator will provide direct access to all study-related sites, source data/data collection forms, and reports for the purpose of monitoring and auditing by the sponsor, and inspection by local and regulatory authorities. The site principal investigator will ensure all study personnel are appropriately trained and applicable documentations are maintained on site.

The DCC will implement quality control procedures beginning with the data entry system and generate data quality control checks that will be run on the database. Any missing data or data anomalies will be communicated to the participating site(s) for clarification and resolution.

13 DATA HANDLING AND RECORD KEEPING

13.1 Data Management Responsibilities

The investigator is responsible to ensure the accuracy, completeness, legibility, and timeliness of the data reported. All source documents should be completed in a neat, legible manner to ensure accurate interpretation of data. Black or blue permanent ink is required to ensure clarity of reproduced copies. When making changes or corrections, cross out the original entry with a single line, and initial and date the change. DO NOT ERASE, OVERWRITE, OR USE CORRECTION FLUID OR TAPE ON THE ORIGINAL.

With the exception of the informed consent/assent documents, data will be entered directly into the electronic CRF (eCRF), which will serve as source data.

13.2 Data Coordinating Center/Biostatistician Responsibilities

Data collection is the responsibility of the study personnel at the participating clinical study site under the supervision of the site principal investigator. During the study, the site principal investigator must maintain complete and accurate documentation for the study.

The data coordinating center for this study will be responsible for data management, quality review, analysis, and reporting of the study data.

13.3 Data Capture Methods

Clinical (including, but not limited to, AE/SAEs) data will be collected and entered directly into eCRFs via a 21 CFR Part 11-compliant internet data entry system provided by the study data coordinating center. The data system includes password protection and internal quality checks, such as automatic range checks, to identify data that appear inconsistent, incomplete, or inaccurate.

13.4 Types of Data

Data for this trial will include clinical and contact information required for bridging to the parent study.

13.5 Study Records Retention

Study records and reports including, but not limited to, eCRFs, source documents, ICFs, and laboratory test results will be retained for 2 years after a marketing application is approved for the study product for the indication for which it is being investigated; or, if no application is to be filed or if the application is not approved for the study product, until 2 years after the investigation is discontinued and the FDA has been notified. These documents will be retained for a longer period, however, if required by local regulations. ICFs for future use will be maintained as long as the sample/specimen exists.

No records will be destroyed without the written consent of the sponsor. It is the responsibility of the sponsor to inform the site principal investigator when these documents no longer need to be retained. The participating VTEU sites must contact DMID for authorization prior to the destruction of any study records.

14 CLINICAL MONITORING

Clinical site monitoring is conducted to ensure that the rights and well-being of trial subjects are protected and that the reported trial data are accurate, complete, and verifiable. Clinical monitoring also ensures that conduct of the trial is in compliance with the currently approved protocol/ amendment(s), ICH, GCP, and with applicable regulatory requirement(s) and Sponsor requirements. Clinical monitoring will also verify that any critical study procedures are completed following specific instructions in the protocol-specific guidance.

Monitoring for this study will be performed by DMID or their designee. Details of clinical site monitoring are documented in a clinical monitoring plan (CMP). The CMP describes in detail who will conduct the monitoring, at what frequency monitoring will be done, at what level of detail monitoring will be performed, and the distribution of monitoring reports. Monitoring visits will include, but are not limited to, review of regulatory files, CRFs, ICFs, medical and laboratory reports, training records, and protocol and GCP compliance. Site monitors will have access to each participating site, study personnel, and all study documentation according to the DMID-approved site monitoring plan. Study monitors will meet with site PIs to discuss any problems and outstanding issues and will document site visit findings and discussions.

15 PUBLICATION POLICY

Following completion of the study, the lead Principal Investigator is expected to publish the results of this research in a scientific journal. All investigators funded by the NIH must submit or have submitted for them to the National Library of Medicine's PubMed Central (http://www.ncbi.nlm.nih.gov/pmc/) an electronic version of their final, peer-reviewed manuscripts upon acceptance for publication, to be made publicly available no later than 12 months after the official date of publication. The NIH Public Access Policy ensures the public has access to the published results of NIH funded research. It requires investigators to submit final peer-reviewed journal manuscripts that arise from NIH funds to the digital archive PubMed Central upon acceptance for publication. Further, the policy stipulates that these papers must be accessible to the public on PubMed Central no later than 12 months after publication.

Refer to:

- NIH Public Access Policy, http://publicaccess.nih.gov/
- NIH Office of Extramural Research (OER) Grants and Funding, http://grants.nih.gov/grants/oer.htm

As of January 2018, all clinical trials supported by the NIH must be registered on ClinicalTrials.gov, no later than 21 days after the enrollment of the first subject. Results of all clinical trials supported by the NIH, generally, need to be submitted no later than 12 months following the primary completion date. A delay of up to 2 years is available for trials that meet certain criteria and have applied for certification of delayed posting.

As part of the result posting a copy of this protocol (and its amendments) and a copy of the Statistical Analysis Plan will be posted on ClincialTrials.gov.

For this trial the responsible party is University of Maryland School of Medicine, Center for Vaccine Development and Global Health, which will register the trial and post results.

The responsible party does not plan to request certification of delayed posting.

Refer to:

- Public Law 110-85, Section 801, Clinical Trial Databases
- 42CFR11
- NIH NOT-OD-16-149

16 APPENDIX

Appendix A. Schedule of Events

Procedure	Enrollment: D35+ of parent study	Home swab self-collection phase: participants will self-collect a nasal swab twice weekly during the following weeks of enrollment in the ancillary swab study*:																					EOS ‡
	D0	1	2	3	4	5	6	7	8	9	10	11	12	13	14	15	16	17	18	19	20	21	22
Informed Consent	X																						
Eligibility Criteria	X																						
Twice weekly nasal swabs	X	X	X	X	X	X	X	X	X	X	X	X	X	X	X	X	X	X	X	X	X	X	X

* The number of weeks that participants will collect two swabs is only an estimate and could range from approximately 6 to 20 weeks depending on when the participant is enrolled and when the cross-over study begins.

‡EOS: End of study; occurs when a swab is collected at the time of the cross-over phase of the parent Phase 3 study.

Final Protocol Version 4.0

TITLE

A nasal swab study to assess the efficacy of vaccination in the prevention of SARS-CoV-2 infection among individuals enrolled in a Phase 3 efficacy trial of a SARS-CoV-2 recombinant spike protein (rS) vaccine with Matrix-M1™ (M1) adjuvant

DMID Protocol Number: 21-0011

DMID Funding Mechanism: 2UM1AI148689-02

Sponsor: NIAID

Lead Principal Investigator: Monica McArthur, MD, PhD

DMID Clinical Project Manager: Peter A. Wolff, MHA

Draft or Version Number: v4.0

Day Month Year
20 AUGUST 2021

STATEMENT OF ASSURANCE

Each Institution will hold a current Federal Wide Assurance (FWA) issued by the Office of Human Research Protections (OHRP) for federally-funded human subjects research. Each FWA will designate at least one Institutional Review Board (IRB)/Independent Ethics Committee (IEC) registered with OHRP, for which the research will be reviewed and approved by the IRB/IEC and will be subject to continuing review [45 CFR 46.103(b)]. The IRB/IEC designated under an FWA may include an institution's IRB/IEC, an independent IRB/IEC, or an IRB/IEC of another institution after establishing a written agreement with that other institution.

STATEMENT OF COMPLIANCE

The study trial will be carried out in accordance with Good Clinical Practice (GCP) and as required by the following:

- United States Code of Federal Regulations (CFR) 45 CFR Part 46: Protection of Human Subjects
- Food and Drug Administration (FDA) Regulations, as applicable: 21 CFR Part 50 (Protection of Human Subjects), 21 CFR Part 54 (Financial Disclosure by Clinical Investigators), 21 CFR Part 56 (Institutional Review Boards), 21 CFR Part 11, and 21 CFR Part 312 (Investigational New Drug Application), 21 CFR 812 (Investigational Device Exemptions)
- International Council on Harmonisation: Good Clinical Practice (ICH E6); 62 Federal Register 25691 (1997); and future revisions
- Belmont Report: Ethical Principles and Guidelines for the Protection of Human Subjects of Research, Report of the National Commission for the Protection of Human Subjects of Biomedical and Behavioral Research
- National Institutes of Health (NIH) Office of Extramural Research, Research Involving Human Subjects, as applicable
- National Institute of Allergy and Infectious Diseases (NIAID) Clinical Terms of Award, as applicable
- Applicable Federal, State, and Local Regulations and Guidance

SIGNATURE PAGE

The signature below provides the necessary assurance that this trial will be conducted according to all stipulations of the protocol, including all statements regarding confidentiality, and according to local legal and regulatory requirements and applicable US federal regulations and ICH E6 Good Clinical Practice (GCP) guidelines.

I agree to conduct the study in compliance with GCP and applicable regulatory requirements.

I agree to conduct the study in accordance with the current protocol and will not make changes to the protocol without obtaining the sponsor's approval and IRB/IEC approval, except when necessary to protect the safety, rights, or welfare of subjects.

Site Investigator Signature:*

Signed: ______________________________ Date: ____________

Name

Title

TABLE OF CONTENTS

STATEMENT OF ASSURANCE 2
STATEMENT OF COMPLIANCE 3
SIGNATURE PAGE 4
TABLE OF CONTENTS 5
LIST OF FIGURES 8
LIST OF ABBREVIATIONS 9
PROTOCOL SUMMARY 12
1 KEY ROLES 14
2 BACKGROUND AND SCIENTIFIC RATIONALE 15
2.1 Background 15
2.2 Scientific Rationale 16
2.2.1 Purpose of Study 16
2.2.2 Study Population 16
2.3 Potential Risks and Benefits 17
2.3.1 Potential Risks 17
2.3.2 Potential Benefits 17
3 STUDY DESIGN, OBJECTIVES AND ENDPOINTS OR OUTCOME MEASURES 18
3.1 Study Design Description 18
3.2 Study Objectives 18
3.2.1 Primary 18
3.2.2 Secondary 19
3.2.3 Exploratory 19
3.3 Study Endpoints or Outcome Measures 19
3.3.1 Primary 19
3.3.2 Secondary 19
3.3.3 Exploratory 20

4 STUDY INTERVENTION/INVESTIGATIONAL PRODUCT 21
5 SELECTION OF SUBJECTS AND STUDY ENROLLMENT AND WITHDRAWAL....22
5.1.1 Subject Inclusion Criteria...22
5.1.2 Subject Exclusion Criteria 22
5.2 Withdrawal from the Study, Discontinuation of Study Product, or Study Termination 23
5.2.1 Withdrawal from the Study or Discontinuation of the Study Product...........23
5.2.2 Subject Replacement...23
5.2.3 Study Termination 23
6 STUDY PROCEDURES 24
6.1 Screening 24
6.2 Enrollment 24
6.3 Twice Weekly Swab Self-Collection at Home...25
6.4 MG Scanner Application 25
6.5 Final Study Visit...26
6.6 Protocol Deviations 26
7 DESCRIPTION OF CLINICAL AND LABORATORY EVALUATIONS 27
7.1 Laboratory Evaluations...27
7.1.1 Research Assays..27
8 ASSESSMENT OF SAFETY...28
8.1 Assessing and Recording Safety Parameters..28
8.1.1 Adverse Events (AEs) Reporting...28
8.2 Safety Oversight (ISM, SMC, DSMB, as applicable)..28
9 HUMAN SUBJECTS PROTECTION 29
9.1 Institutional Review Board/Independent Ethics Committee 29
9.2 Informed Consent Process 30
9.2.1 Requirements for Permission by Parents/Guardians and Assent by Children (in case of a minor) 32

9.2.2 Other Informed Consent Procedures.....32
9.3 Consent for Future Use of Stored Specimens and Data.....33
9.4 Subject Confidentiality.....33
9.5 Certificate of Confidentiality.....33
9.6 Costs, Compensation, and Research Related Injuries.....34
10 STATISTICAL CONSIDERATIONS.....35
10.1 Sample Size Considerations.....35
10.2 Populations for Analysis.....36
10.3 Analytic considerations.....37
11 SOURCE DOCUMENTS AND ACCESS TO SOURCE DATA/DOCUMENTS.....39
12 QUALITY CONTROL AND QUALITY ASSURANCE.....40
13 DATA HANDLING AND RECORD KEEPING.....41
13.1 Data Management Responsibilities.....41
13.2 Data Coordinating Center/Biostatistician Responsibilities.....41
13.3 Data Capture Methods.....41
13.4 Types of Data.....41
13.5 Study Records Retention.....42
14 CLINICAL MONITORING.....43
15 PUBLICATION POLICY.....44
16 LITERATURE REFERENCES.....46
17 APPENDIX.....47
Appendix A. Schedule of Events.....47
Appendix B. Protocol Change History.....48

LIST OF FIGURES

Figure 1 Power to detect vaccine efficacy against infection with 2000 participants, assuming follow-up for 6, 12 or 20 weeks and incidence rate equal to 2.9 per 100,000 person-weeks (left panel), 4.8 per 100,000 person-weeks (center panel), and 5.7 per 100,000 person-weeks (right panel)..36

LIST OF ABBREVIATIONS

AE	Adverse Event/Adverse Experience
A/VMS	Asymptomatic/Very Mild Symptomatic Infection
BLA	Biologics License Applications
CFR	Code of Federal Regulations
CI	Confidence Interval
CIOMS	Council for International Organizations of Medical Sciences
CMS	Clinical Material Services
CONSORT	Consolidated Standards of Reporting Trials
COVID-19	Coronavirus Disease 2019
CoVPN	COVID-19 Prevention Network
CRF	Case Report Form
CRO	Contract Research Organization
CSR	Clinical Study Report
CVD	Center for Vaccine Development and Global Health
DCC	Data Coordinating Center
DHHS	Department of Health and Human Services
DMID	Division of Microbiology and Infectious Diseases, NIAID, NIH, DHHS
DSMB	Data and Safety Monitoring Board
eCRF	Electronic Case Report Form
FDA	Food and Drug Administration
FDAAA	Food and Drug Administration Amendments Act

FWA	Federal Wide Assurance
GCP	Good Clinical Practice
HIPAA	Health Insurance Portability and Accountability Act
IB	Investigator’s Brochure
ICF	Informed Consent Form
ICH	International Council on Harmonisation
ICMJE	International Committee of Medical Journal Editors
IDE	Investigational Device Exemption
IEC	Independent or Institutional Ethics Committee
IGS	Institute for Genome Sciences
IND	Investigational New Drug Application
IRB	Institutional Review Board
ISM	Independent Safety Monitor
JAMA	Journal of the American Medical Association
M1	Matrix-M1™ adjuvant
MedDRA®	Medical Dictionary for Regulatory Activities
MOP	Manual of Procedures
N	Number (typically refers to subjects)
NDA	New Drug Application
NEJM	New England Journal of Medicine
NIAID	National Institute of Allergy and Infectious Diseases, NIH, DHHS
NIH	National Institutes of Health
OHRP	Office for Human Research Protections

OHSR	Office for Human Subjects Research
PHI	Protected Health Information
PI	Principal Investigator
PREVENT-19	Pre-fusion Protein Subunit Vaccine Efficacy Novavax Trial COVID-19
QA	Quality Assurance
QC	Quality Control
rS	Recombinant spike protein of SARS-CoV-2
rS/M1	SARS-CoV-2 recombinant spike (rS) protein nanoparticle vaccine with M1 adjuvant
SAE	Serious Adverse Event/Serious Adverse Experience
SARS-CoV-2	Severe Acute Respiratory Syndrome Coronavirus 2
SMC	Safety Monitoring Committee
SOP	Standard Operating Procedure
UMSOM	University of Maryland School of Medicine
US	United States
VE	Vaccine Efficacy
WHO	World Health Organization

PROTOCOL SUMMARY

**Title:**	A nasal swab study to assess the efficacy of vaccination in the prevention of SARS-CoV-2 infection among individuals enrolled in a Phase 3 efficacy trial of a SARS-CoV-2 recombinant spike protein (rS) vaccine with Matrix-M1™ (M1) adjuvant
**Design of the Study:**	In this ancillary swab study, up to 3,000 adolescent participants 12-17 years of age in the Phase 3 SARS-CoV-2 vaccine trial (PREVENT-19) sponsored by Novavax will be sampled twice weekly for approximately 16-28 weeks with nasal swabs for detection of SARS-CoV-2 infection by RT-PCR and incident infections tallied.
**Study Phase:**	Observational
**Study Population:**	Subset of participants in Protocol 2019nCoV-301, entitled "A Phase 3, Randomized, Observer-Blinded, Placebo-Controlled Study to Evaluate the Efficacy, Safety, and Immunogenicity of a SARS-CoV-2 Recombinant Spike Protein Nanoparticle Vaccine (SARS-CoV-2 rS) with Matrix-M1™ Adjuvant in Adult Participants ≥ 18 Years with a Pediatric Expansion to adolescents (12 to 17 years)" (PREVENT-19)
**Number of Sites:**	Up to 75 sites located in the US
**Description of Study Product or Intervention:**	Study participant will be randomized to receive either SARS-CoV-2 rS/M1 vaccine or placebo (2:1 allocation) on days 0 and 21 as part of the parent PREVENT-19 trial. In the current protocol, no interventional product will be administered.
**Study Objectives:**	Primary:

- To estimate the efficacy of SARS-CoV-2 rS/M1 vaccine against infection

Secondary:

- To estimate the efficacy of SARS-CoV-2 rS/M1 vaccine against asymptomatic/very mildly symptomatic infection
- To determine the durability of efficacy of SARS-CoV-2 rS/M1 vaccine against infection
- To determine the durability of efficacy of SARS-CoV-2 rS/M1 vaccine against asymptomatic/very mildly symptomatic infection

Exploratory:

- To determine the sequence of breakthrough SARS-CoV-2 infections
- To estimate the VE on duration of infection
- To estimate the VE against SARS-CoV-2 viral load as a proxy of transmission
- To estimate the efficacy of SARS-CoV-2 rS/M1 vaccine against a composite outcome of asymptomatic/ very mildly symptomatic infection plus pre-symptomatic infection
- To explore whether observed VE against COVID-19 illness reflects a transition from symptomatic into asymptomatic infections versus an absolute reduction in all SARS-CoV-2 infections

Duration of Individual Subject Participation: Approximately 4-7 months for each participant

Estimated Time to Last Subject/Last Study Day: Approximately 9 months from study activation to last subject/last study day

1 KEY ROLES

**Lead Principal Investigator:**	Monica McArthur, MD PhD, Assistant Professor of Pediatrics<br>Center for Vaccine Development and Global Health<br>University of Maryland School of Medicine
**DMID Clinical Project Manager:**	Peter A. Wolff, MHA, Clinical Project Manager<br>DMID, NIAID, NIH, DHHS
**Statistical and Data Coordinating Center:**	Fred Hutchinson Cancer Research Center, Statistical Center for HIV/AIDS Research and Prevention (SCHARP)

2 BACKGROUND AND SCIENTIFIC RATIONALE

2.1 Background

The primary goal of current COVID-19 vaccine trials is to measure vaccine efficacy (VE) against clinically significant infection. The aim is to save lives, reduce morbidity and sequelae, and alleviate the workload and financial burdens to the health care system. A hallmark of SARS-CoV-2 is its ability to efficiently replicate in the human host without or before causing symptoms. During these subclinical infections, individuals shed virus in the upper respiratory tract and may transmit SARS-CoV-2. Most trials do not directly assess vaccine efficacy against acquisition of virus overall as there is no routine sampling of participants (who do not meet endpoint definitions for illness) to detect SARS-CoV-2. Asymptomatic seroconversion to viral antigens not contained in the vaccine is often used as a surrogate, but the precision of these estimates is subject to variability in the sensitivity and specificity of the serologic assays and the durability of antibody following subclinical infection and may not predict viral shedding. Yet the vaccine effect on infection is important to understand, both for its potential impact on transmission and to better characterize the vaccine effect on individuals.

On a population level, vaccine efficacy against all infection, including asymptomatic or very mildly symptomatic infection, is an important endpoint. The impact of a pandemic virus's ability to spread sub-clinically permeates the educational systems, essential infrastructure and operations, and the social, psychological, financial, and political fabric of our society. For policy decision-makers, knowledge of vaccine efficacy against asymptomatic or very mildly symptomatic infection is critical for vaccine allocation and distribution, prioritization of groups to receive vaccine, re-opening and community mitigation strategies, and determining the need for continued individual infection control measures such as social distancing and face coverings. To address these knowledge gaps, we will conduct an ancillary swab study to estimate vaccine efficacy against acquisition of SARS CoV-2 infection, regardless of symptomatology.

As the SARS-CoV-2 pandemic continues to evolve, it is imperative to gather additional information on durability of vaccine induced protection as well as the effects of vaccination on duration and magnitude of viral shedding. This is particularly important in the context of SARS-CoV-2 variants of concern, such as the delta variant.

This ancillary swab study will leverage the ongoing placebo-controlled Phase 3 SARS-CoV-2 vaccine trial (PREVENT-19 NCT04611802) pediatric expansion study sponsored by Novavax (Gaithersburg, MD) that includes 3,000 adolescents (12-17 years of age). The primary aim of the pediatric expansion study of PREVENT-19 is to assess the safety and effectiveness of a SARS-CoV-2 recombinant spike (rS) protein nanoparticle vaccine administered with the Matrix-M1™ adjuvant (herein designated rS/M1 vaccine). Participants in the pediatric expansion are randomized to receive either SARS-CoV-2 rS/M1 vaccine or placebo (2:1 allocation) on days 0 and 21. Beginning August 10, 2021, a blinded crossover of the pediatric extension of the PREVENT-19 study was initiated. Participants in the pediatric expansion will receive two doses of the alternate treatment that they received initially. In other words, those who initially received 2 doses of placebo spaced 21 days apart will receive 2 dose of vaccine, and those who initially received 2 spaced doses of vaccine will receive 2 spaced doses of placebo. This will allow all participants to know that he/she has received active vaccine product while maintaining blinded status as to when the active product was received.

2.2 Scientific Rationale

2.2.1 Purpose of Study

The primary question that will be addressed in this ancillary study is whether SARS CoV-2 vaccines can prevent overall acquisition of infection and, therefore, have the ability to interrupt transmission. However, it is likely that some vaccinated individuals will continue to shed virus asymptomatically. Therefore, vaccine efficacy against the magnitude and duration of shedding will be included as exploratory aims, as these parameters can be considered to be proxies of reduced transmission potential, recognizing that definitive results require direct measurement of the frequency of infection in contacts of vaccinated compared to unvaccinated individuals.

2.2.2 Study Population

The pediatric expansion to the Phase 3 PREVENT-19 trial sponsored by Novavax is estimated to start enrollment in April-May 2021. The expansion will enroll ~3,000 adolescents (12-17 years of age). All children who are participating in the Phase 3 pediatric expansion trial are eligible for enrollment in the ancillary swab study. Approximately 75 sites are expected to participate in the pediatric expansion.

2.3 Potential Risks and Benefits

2.3.1 Potential Risks

Risks associated with this ancillary study are primarily associated with potential loss of confidentiality. The study team will make every effort to protect each participant's privacy and confidentiality. Participants will be assigned a unique study number (Subject ID) for all data and sample collection. Paper data collection forms and specimen labels will be identified only with Subject ID. Study files will be stored in locked file cabinets and electronic data collection and storage tools will be password protected. Access to participant data will be limited to authorized study personnel.

This study presents no greater than minimal risk to the participants. Risks associated with anterior nares nasal swab collection may include sneezing, eye watering, coughing, or possible epistaxis.

Notably, the ancillary swab study will be performed at the convenience of the Phase 3 study, where the goals of the Phase 3 study will take priority over this ancillary study (i.e., consideration for the Phase 3 study objectives and endpoints will supersede those of the ancillary study). The ancillary study is designed to generate additional valuable data on efficacy of SARS-CoV-2 rS/M1 vaccine against infection, including asymptomatic/very mildly symptomatic infection.

2.3.2 Potential Benefits

There are no direct benefits to participants participating in this ancillary study; however, the results of this ancillary study may provide valuable information that can help others in the future.

3 STUDY DESIGN, OBJECTIVES AND ENDPOINTS OR OUTCOME MEASURES

3.1 Study Design Description

In this ancillary swab study, up to 3,000 participants who are participating in the PREVENT-19 Pediatric Expansion study and who provide informed consent/assent for the ancillary swab study will be sampled twice weekly starting at the day 21 visit, or as soon as possible thereafter, of the initial injection period until December 17, 2021 (~16-28 weeks) with nasal swabs for detection of SARS-CoV-2 infection by RT-PCR and incident infections tallied. Data sources from the Phase 3 trial (e.g., study product assignment, daily symptomatology) will be used in the ancillary study to estimate the vaccine efficacy against SARS-CoV-2 infection, including asymptomatic infection or mildly symptomatic infection not meeting the COVID-19 primary endpoint definition for the Phase 3 study. Longer infections with higher viral loads, as a proxy for transmission potential, will be captured in this design.

Participants in the PREVENT-19 Phase 3 trial (pediatric expansion study) are randomized to receive either SARS-CoV-2 rS/M1 vaccine or placebo (2:1 allocation) on days 0 and 21. The pediatric expansion study will have a “blinded-crossover” after ~3 months of follow-up after the completion of the initial set of vaccinations. Pediatric participants will be scheduled for administration of 2 injections of the alternate study material 21 days apart.

The study design will allow a comparison of the incidence and other characteristics of SARS-CoV-2 infection in recipients of vaccine versus placebo. In addition, we have amended the initial design to continue swabbing after the blinded cross-over occurs and all participants are vaccinated. The cross-over coincides with the emergence of the delta Variant of Concern (VOC) when the rate of breakthrough infections is expected to increase. By extending swabbing, we will be able to assess the duration of vaccine efficacy by comparing the incidence and characteristics of break-through infection among participants who were vaccinated in April-June to those who were vaccinated in August-September 2021.

3.2 Study Objectives

3.2.1 Primary

- To estimate the efficacy of SARS-CoV-2 rS/M1 vaccine against infection

3.2.2 Secondary

- To estimate the efficacy of SARS-CoV-2 rS/M1 vaccine against asymptomatic/ very mildly symptomatic infection
- To determine the durability of efficacy of SARS-CoV-2 rS/M1 vaccine against infection
- To determine the durability of efficacy of SARS-CoV-2 rS/M1 vaccine against asymptomatic/very mildly symptomatic infection

3.2.3 Exploratory

- To determine the sequence of breakthrough SARS-CoV-2 infections
- To estimate the VE on duration of infection
- To estimate the VE against SARS-CoV-2 viral load as a proxy of transmission
- To estimate the efficacy of SARS-CoV-2 rS/M1 vaccine against a composite outcome of asymptomatic/ very mildly symptomatic infection plus pre-symptomatic infection
- To explore whether observed VE against COVID-19 illness reflects a transition from symptomatic into asymptomatic infections versus an absolute reduction in all SARS-CoV-2 infections

3.3 Study Endpoints or Outcome Measures

3.3.1 Primary

- Reverse transcriptase polymerase chain reaction (RT-PCR)-confirmed SARS-CoV-2 infection detected in nasal swabs collected twice weekly from the day 21 visit or thereafter, until ancillary study end in the vaccine group and the placebo group

3.3.2 Secondary

- RT-PCR-confirmed SARS-CoV-2 infection detected in nasal swabs collected twice weekly from the day 21 visit or thereafter, until ancillary study end, without COVID-19 illness as defined in the primary Phase 3 study endpoint
- RT-PCR-confirmed SARS-CoV-2 infection detected in nasal swabs collected twice weekly from the crossover dose 2 visit (or thereafter) until ancillary study end among the group who initially received vaccine compared to the group who initially received placebo

- RT-PCR-confirmed SARS-CoV-2 infection detected in nasal swabs collected twice weekly from the crossover dose 2 visit (or thereafter) until ancillary study end among the group who initially received vaccine compared to the group who initially received placebo without COVID-19 illness as defined in the primary Phase 3 study endpoint

3.3.3 Exploratory

- Viral genome sequence of PCR-positive samples
- Duration of shedding as indicated by RT-PCR in vaccinated vs placebo recipients
- Reduction in viral load (and perhaps subgenomic RNA), as a proxy for transmission, in PCR-positive samples from vaccinated vs placebo recipients
- Proportion of days that are symptom-free on days that positive swabs are collected
- Ratio of asymptomatic to symptomatic infections in vaccinated vs placebo recipients. Data on symptomatology will be extracted from the Phase 3 trial database, which will be separate but linkable to the ancillary study database.

4 STUDY INTERVENTION/INVESTIGATIONAL PRODUCT

Study participant will be randomized to receive either SARS-CoV-2 rS/M1 vaccine or placebo (2:1 allocation) on days 0 and 21 as part of the parent PREVENT-19 trial. In the current protocol, no interventional product will be administered. Each participant's blinded allocation will be accessed through the PREVENT-19 trial database during analysis.

5 SELECTION OF SUBJECTS AND STUDY ENROLLMENT AND WITHDRAWAL

Subject Inclusion and Exclusion Criteria must be confirmed by a study clinician licensed to make medical diagnoses.

No exemptions will be granted on Subject Inclusion/Exclusion Criteria.

Eligibility Criteria

5.1.1 Subject Inclusion Criteria

Each participant must meet all of the following criteria to be enrolled in this study:

1. Participant in the parent study
2. Less than 18 years and ≥ 12 years of age at time of enrollment
3. Ability and willingness to give informed consent/assent prior to study enrollment and comply with the study procedures
4. Received two doses of study product
5. Providing surveillance data on COVID-19 symptoms as described in the parent protocol
6. Access to a cell phone or Wi-Fi/Camera enabled device with ability to download and use the MG ScannerTM Application
7. Active independent or unique email address

5.1.2 Subject Exclusion Criteria

Participants meeting the following criterion will be excluded from the study:

1. Any condition that would, in the opinion of the investigator, place the participant at an unacceptable risk of injury or render him/her unable to comply with the study requirements.

5.2 Withdrawal from the Study, Discontinuation of Study Product, or Study Termination

5.2.1 Withdrawal from the Study or Discontinuation of the Study Product

Subjects may voluntarily withdraw their consent for study participation at any time without penalty or loss of benefits to which they are otherwise entitled.

An investigator may also withdraw a subject from participating in the study for any reason. If a subject withdraws or is withdrawn prior to completion of the study, the reason for this decision must be recorded in the case report forms (CRFs).

The reasons, might include, but are not limited to the following:

- Subject no longer meets eligibility criteria
- Subject becomes noncompliant
- Medical disease or condition, or new clinical finding(s) for which continued participation, in the opinion of the investigator might compromise the safety of the subject, interfere with the subject's successful completion of this study, or interfere with the evaluation of responses
- Subject lost to follow-up
- Subject unblinded in parent study

The investigator will inform the participant that already collected data will be retained and analyzed even if the participant withdraws from this study.

5.2.2 Subject Replacement

Subjects who withdraw, or are withdrawn from this study, or are lost to follow-up after signing the informed consent form (ICF) will not be replaced.

5.2.3 Study Termination

If the study is prematurely terminated by the sponsor, any regulatory authority, or the investigator for any reason, the investigator will promptly inform the study subjects and assure appropriate therapy or follow-up for the subjects, as necessary. The investigator will provide a detailed written explanation of the termination to the IRB/IEC.

6 STUDY PROCEDURES

6.1 Screening

Participants in the Phase 3 trial will have the ancillary study explained to them by study personnel during their day 21 visit or at another study encounter. If contact at the day 21 visit is not possible, a later appointment may be arranged for enrollment into the ancillary swab study. Those with expressed interest, will provide informed consent/assent prior to any study procedures. The consent form will include permission for investigators to review the participant's research records and to link with the parent study database at the appropriate time to obtain related information on randomization assignment, COVID-19 symptomatology, and RT-PCR results from study-specific swabs or other relevant events.

Informed consent/assent is required prior to enrollment and prior to any study-related procedures. Parental or guardian permission will be obtained for all children. Assent will be obtained from children according to the IRB requirements at each site. To ensure that the participant's rights are protected and that all study participants receive, read, have explanation of and sign an informed consent/assent prior to enrolling in this study, research team members will follow the informed consent process outlined in site-specific SOPs and as approved by the single IRB, as applicable.

Following the attainment of informed consent/assent, eligibility criteria will be reviewed. The screening visit will occur concurrently with enrollment in most cases. A window of 7 days between screening and enrollment is allowed.

6.2 Enrollment

After obtaining informed consent/assent as above, study staff will collect a limited array of demographic and clinical information from the participant. An application will be downloaded onto the participant's or participant's parent's smart phone or Wi-Fi/camera enabled device that will permit the participant to scan the bar code on nasal swab vials at home prior to sending them to the ancillary swab study central laboratory at the University of Maryland School of Medicine (UMSOM)/Institute for Genome Sciences (IGS) for SARS CoV-2 RT-PCR testing and storage. The application will log the date/time of each sample collection during the home swabbing period. No Protected Health Information (PHI) will be transferred through the application. Study personnel will instruct the participant on the methods for self-collection and shipment of nasal swab specimens. Participants will receive a packet of swab kits for their home use.

If the participant/participant's parent does not have an appropriate device available, University of Maryland will provide Wi-Fi and camera enabled devices for participants to use the MG Scanner mobile application to track their bi-weekly swabs. These devices will not have active cellular service or data plans. The devices will not be offered to all participants. They will be provided only to those who do not have a personal device that is capable of downloading and using the MG Scanner mobile application, or parents who do not have an adequate number of devices to accommodate the participants in their household (multiple siblings enrolled). Study devices will be shipped overnight from the University of Maryland to the participants home directly after enrollment. Site coordinators will assist study participants with the final app registration steps after confirmation that they have received their study device. The helpline will also be available to participants for any questions or concerns with downloading and setting up the MG Scanner application on the study devices. Devices will not be collected or returned at the end of the study.

6.3 Twice Weekly Swab Self-Collection at Home

Beginning as soon as possible, on or after day 21, or soon thereafter, (week 1 in Appendix A), the participant will self-collect an anterior nares nasal swab for SARS CoV-2 RT-PCR testing twice weekly. If the child is not able to self-collect independently, collection may be performed by the parent or by the child under parental supervision. For example, each participant will collect a swab on the Monday (+1) and Thursday (+1) of each week. The duration of nasal swabbing for each participant cannot be determined with precision at this time; it might range from approximately 16-28 weeks depending on when the participant was enrolled. Home nasal swabs will be retrieved by a courier and/or mail delivery service and delivered to the UMSOM/IGS laboratory.

Reminder e-mails will be sent on the day of expected sample collection. If a swab is missed, the participant/participant's parent will receive a phone call reminder. As this is a research study, the results of these tests will not be released to the participant/parent.

6.4 MG Scanner Application

The MG Scanner application is an internally developed mobile application for iOS and Android devices. The application is designed to facilitate the registration of participants for COVID-19 testing through indirect interaction with the Zoran system. MG scanner also provides functionality for the scanning and registration of collected samples for testing.

In this system the user registers his/her device through the application via an emailed invitation. This registration binds the device ID to the participant's medical record number (MRN). The MRN is unique to each individual and an individual only has one MRN in the Zoran system. This MRN is separate from the participant's hospital/clinic MRN or the parent study ID. The user can then submit a sample for testing through a sample scanning component of the app. The app will scan two globally unique IDs from pre-labeled sample collection tubes and those IDs will be bound to the participant MRN as a pending specimen. Scanning of two globally unique IDs ensures the tube only occurs once and is positively associated with the participant MRN. If one barcode is damaged during the collection or transport process the specimen can still be positively associated with the submitting participant.

6.5 Final Study Visit

The final ancillary study swab will be collected by December 17, 2021 at which point the participant's ancillary study participation will be completed (Appendix).

6.6 Protocol Deviations

A protocol deviation is any noncompliance with the clinical trial protocol, GCP, or protocol-specific Manual of Procedures (MOP) requirements. Failure of a participant to collect nasal swabs at home or collection outside of the window will not be captured as protocol deviations as it will not impact study safety or overall scientific integrity. The noncompliance may be either on the part of the subject, the investigator, or the study site staff. As a result of deviations, corrective actions should be developed by the site and implemented promptly. It is the responsibility of the site Principal Investigator and other study personnel to use continuous vigilance to identify and report protocol deviations. All individual protocol deviations will be addressed in subject study records. All protocol deviations, either individual, product, or site-specific will be collected and the record stored in a sponsor-determined location. Protocol deviations must be sent to the local IRB/IEC per its guidelines. The site Principal Investigator and other study personnel are responsible for knowing and adhering to their IRB/IEC requirements.

7 DESCRIPTION OF CLINICAL AND LABORATORY EVALUATIONS

7.1 Laboratory Evaluations

7.1.1 Research Assays

At the enrollment visit, each participant will be provided kits for collection containing tubes and swabs along with collection instructions in each kit. They will be instructed on the proper method of obtaining nasal swabs during the visit. Additionally, the participants will be shown how to register with the application (MG Scanner™) to log each sample when it is collected.

At each required timepoint, the participant or his/her parent will collect an anterior nares nasal specimen of nasal secretions by swabbing the anterior nares of each nostril according to detailed standardized procedures demonstrated in the provided instructions. The swab will be placed in a vial containing buffer solution and maintained at room temperature.

The application will log the date/time of each sample collection during the observation period.

The swabs will be sent to the University of Maryland School of Medicine, Institute of Genome Sciences (UMSOM/IGS) laboratory for analyses.

7.1.1.1 Laboratory Specimen Preparation, Handling, and Storage

Samples will be shipped to UMSOM/IGS and will be run as they are received. Stability studies have demonstrated that all sample storage and shipping can be done at room temperature without affecting the SARS-CoV-2 RT-PCR test performance.

7.1.1.2 Laboratory Specimen Shipping

Participants will package collected swabs according to the provided instructions in provided shipping envelopes or boxes addressed to the UMSOM/IGS laboratory. Shipping to the UMSOM/IGS laboratory will occur every 1-2 weeks.

8 ASSESSMENT OF SAFETY

8.1 Assessing and Recording Safety Parameters

8.1.1 Adverse Events (AEs) Reporting

No adverse reactions are anticipated in this study, and the study procedures present no anticipated risks beyond the completion of the study. It is anticipated that the participants may experience discomfort when anterior nares nasal swab specimens are being collected; however, the risks and discomfort in this study are not greater than those ordinarily encountered during the routine collection of the anterior nares nasal swabs at a clinician's office.

Vaccine related AEs will not be collected as part of this ancillary study. The Sponsor will collect AE information via the CRF. AEs that last greater than 15 minutes will be collected from the time of swab collection to the time that the subject completes the study. Such data collection will include the start and stop dates of the event, causality with the swab collection (not related, related), outcome (ongoing, recovered, recovered with sequelae, not recovered, fatal), code for seriousness (SAE, not serious), and intensity (mild, moderate, or severe). Only events that are moderate or severe in intensity will be recorded. The definitions of mild, moderate, or severe are as follows:

1 = Mild (awareness of a symptom but the symptom is easily tolerated)

2 = Moderate (discomfort enough to cause interference with usual activity)

3 = Severe (incapacitating; unable to perform usual activities; requires absenteeism or bed rest)

8.2 Safety Oversight (ISM, SMC, DSMB, as applicable)

A Safety oversight Committee is not required for this minimum risk study. Safety oversight will be the responsibility of the Principal Investigator

9 HUMAN SUBJECTS PROTECTION

9.1 Institutional Review Board/Independent Ethics Committee

Each site principal investigator will obtain IRB approval for this protocol to be conducted at his/her research site(s) and send supporting documentation to the DMID before initiating recruitment of subjects. The investigator will submit applicable information to the IRB/IEC on which it relies for the review, to conduct the review in accordance with 45 CFR 46, ICH E6 GCP, and as applicable, 21 CFR 56 (Institutional Review Boards) and 21 CFR 50 (Protection of Human Subjects), other federal, state, and local regulations. The IRB/IEC must be registered with OHRP as applicable to the research. DMID must receive the documentation that verifies IRB/IEC-approval for this protocol, associated informed consent documents, and upon request any recruitment material and handouts or surveys intended for the subjects, prior to the recruitment and enrollment of subjects.

Any amendments to the protocol or consent materials will be approved by the IRB/IEC before they are implemented. IRB/IEC review and approval will occur at least annually throughout the enrollment and follow-up of subjects and may cease if annual review is no longer required by applicable regulations and the IRB/IEC. The investigator will notify the IRB/IEC of deviations from the protocol and reportable SAEs, as applicable to the IRB/IEC policy.

Each institution engaged in this research will hold a current Federalwide Assurance (FWA) issued by the Office of Human Research Protection (OHRP) for federally funded research.

A single IRB of record, WCG IRB, will be accountable for compliance with regulatory requirements for this multi-centered study, at participating sites. Written reliance agreements between the single IRB and participating sites will be required. The reliance agreements will set forth the specific responsibilities of the IRB and each participating site. Participating sites will then rely on the IRB of record to satisfy the regulatory requirements relevant to the IRB review. The participating sites will maintain essential required documentation of IRB reviews, approvals, and correspondence, and must provide copies of any agreements and essential documentation to the DMID or regulatory authorities upon request.

The IRB/IEC will determine that adequate provisions are made for soliciting the permission of each child's parent(s) or legal guardian(s), including whether permission of one parent/guardian is sufficient for research or whether permission is to be obtained from both parents/guardians.

The IRB/IEC will determine how consent from subjects will be obtained when participation in the study is ongoing, and the subject has reached the age of majority.

9.2 Informed Consent Process

Informed consent is a process that is initiated prior to an individual agreeing to participate in a trial and continuing throughout the individual's trial participation. Before any study procedures are performed, informed consent will be obtained and documented. Subjects will receive a concise and focused presentation of key information about the clinical trial, verbally and with a written consent form. The explanation will be organized and presented in lay terminology and language that facilitates understanding why one might or might not want to participate.

An investigator or designee will describe the protocol to potential subjects' parent(s)/guardian(s). The key information about the purpose of the study, the procedures and experimental aspects of the study, risks and discomforts, any expected benefits to the subject, and alternative treatment will be presented first to the subject's parent(s)/guardian(s).

Subjects' parent(s)/guardian(s) will also receive an explanation that the trial involves research, and a detailed summary of the proposed study procedures. This will include aspects of the trial that are experimental, any expected benefits, all possible risks, and the expected duration of the subject's participation in the trial.

Subjects' parent(s)/guardian(s) will be informed that they will be notified in a timely manner if information becomes available that may be relevant to their willingness to continue participation in the trial. Subjects will receive an explanation as to whether any compensation and any medical treatments are available if injury occurs, and, if so, what they consist of, or where further information may be obtained. Subjects' parent(s)/guardian(s) will be informed of the anticipated financial expenses, if any, to the subject's parent(s)/guardian(s) for their child's participation in the trial, as well as any anticipated prorated payments, if any, to the subject and his/her parent(s)/guardian(s) for participation in the trial. They will be informed of whom to contact (e.g., the investigator) for answers to any questions relating to the research project.

Information will also include the foreseeable circumstances and/or reasons under which the subject's participation in the trial may be terminated. The subjects' parent(s)/guardian(s) will be informed that participation is voluntary and that they are free to withdraw from the study for any reason at any time without penalty or loss of benefits to which the subject and his/her parent(s)/ guardian(s) are otherwise entitled.

The extent of the confidentiality of the subjects' records will be defined, and subjects' parent(s)/guardian(s) will be informed that applicable data protection legislation will be followed. Subjects will be informed that the monitor(s), auditors(s), IRB, NIAID, and regulatory authority(ies) will be granted direct access to the subject's original medical records for verification of clinical trial procedures and/or data without violating the confidentiality of the subject, to the extent permitted by the applicable laws and regulations, and that, by signing a written informed consent form, the subject's parent(s)/guardian(s) is/are authorizing such access.

Subjects' parent(s)/guardian(s) will be informed that records identifying the subject will be kept confidential, and, to the extent permitted by the applicable laws and/or regulations, will not be made publicly available and, if the results of the trial are published, the subject's identity will remain confidential. Subjects' parent(s)/guardian(s) will be informed whether private information collected from this research and/or specimens will be used for additional research, even if identifiers are removed.

Subjects' parent(s)/guardian(s) will be allowed sufficient time to consider participation in this research trial and have the opportunity to discuss this trial with their family, friends or legally authorized representative, or think about it prior to agreeing to participate.

Informed consent forms will be IRB-approved, and subjects' parent(s)/guardian(s) will be asked to read and review the consent form. Subjects' parent(s)/guardian(s) must sign the informed consent form prior to starting any study procedures being done specifically for this trial.

Once signed, a copy of the informed consent form will be given to the subjects' parent(s)/guardian(s) for their records. The subjects' parent(s)/guardian(s) may withdraw consent at any time throughout the course of the trial. The rights and welfare of the subject(s) will be protected by emphasizing that the quality of their medical care will not be adversely affected if the parent(s)/guardian(s) decline to allow their child to participate in this study.

New information will be communicated by the site principal investigator or designee to subjects' parent(s)/guardian(s) who consent for their child to participate in this trial in accordance with IRB requirements. The informed consent document will be updated, and subjects' parent(s)/guardian(s) will be re-consented per IRB requirements, if necessary. Subjects' parent(s)/guardian(s) will be given a copy of all informed consent forms that they sign.

9.2.1 Requirements for Permission by Parents/Guardians and Assent by Children (in case of a minor)

Investigators will follow IRB/IEC requirements for enrollment of minors in this study. Minors will be informed about the study to the extent understandable to the minor. Investigators or designee will conduct the consent process with the parent(s)/legal guardian(s), who will be given an IRB/IEC-approved permission form, which may be referred to as a consent form, to read, review, and sign prior to any study procedures. The parent(s)/legal guardian(s) will be provided meaningful study information including a statement that this study involves research, the child may not benefit from the trial, and the study involves risk. The required elements will be clearly presented, including the purpose of the study, the experimental procedures, the potential risks and discomforts, known adverse effects, possible benefits of the study for the subject, use and disclosure of private information, and other elements that are part of obtaining proper consent. The subject's parent(s)/legal guardian(s) will be allowed sufficient time to discuss questions with the investigator or designee.

The investigator or designee will describe in simplified terms the details of the study procedures, risks and discomforts, benefits, and other consent elements, as appropriate. A separate IRB/IEC-approved assent form will be used for the minor (as appropriate), who may read and sign the form, or have it read to him/her prior to participation in study procedures. Assent may be obtained verbally or waived when approved by the IRB/IEC as appropriate to age. If a child declines to participate in the trial when assent is required by the IRB/IEC, the subject will not be enrolled even though the parent(s)/guardian(s) have provided permission.

To ensure that consent is an ongoing process throughout the subject's participation in the study, the investigator and staff will review information as needed with the subject (as appropriate) and the parent(s)/legal guardian(s) and confirm that assent and permission are continuing. The permission and assent documents will be updated when new information is acquired that may impact the decision to continue in the study, and the subject's assent and the parent(s)/legal guardian's permission will be obtained, as applicable.

The subject who reaches the age of majority will be consented at the next visit prior to study procedures. When no further visits are planned but the subject's participation is ongoing, the consent will be obtained via IRB/IEC-approved processes.

9.2.2 Other Informed Consent Procedures

Illiterate Subjects/Parent(s)/Guardian(s)

If subjects' parent(s)/guardian(s) are illiterate**,** they will not be allowed to participate in the study.

9.3 Consent for Future Use of Stored Specimens and Data

Residual samples/specimens are those that are left over after protocol-specified testing and this study has been completed. Subjects' parent(s)/guardian(s) will be asked for permission to keep any remaining (residual) specimens (extracted viral RNA) derived from nasal swab samples for possible use in future research studies, such as examining additional virologic assessments. These residual specimens will be stored, coded, indefinitely at UMSOM/IGS. The recipients of specimens will be informed that these specimens have a NIH certificate of confidentiality. The information provided to a recipient will not contain direct identifiable information. The research will not include human whole genome sequencing, nor will your child's nasal swabs be used to generate a cell line for genetic testing.

9.4 Subject Confidentiality

Subject confidentiality is strictly held in trust by the participating investigators, their staff, and the sponsor(s) and their agents. This confidentiality includes documentation, investigation data, subject's clinical information, and all other information generated during participation in the study. No information concerning the study, or the data generated from the study, will be released to any unauthorized third party without prior written approval of the DMID and the subject's parent(s)/guardian(s). Subject confidentiality will be maintained when study results are published or discussed in conferences. The study monitor, or other authorized representatives of the sponsor or governmental regulatory agencies, may inspect all documents and records required to be maintained by the investigator, including but not limited to, medical records (office, clinic, or hospital) and pharmacy records for the subjects in this study. The clinical study site will permit access to such records.

All records will be kept locked and all computer entry and networking programs will be carried out with coded numbers only and with password-protected systems. All non-clinical specimens, evaluation forms, reports, and other records that leave the site will be identified only by a coded number.

9.5 Certificate of Confidentiality

To protect privacy, we have received a Certificate of Confidentiality. With this Certificate, the researchers cannot be forced to release information that may identify the research subject, even by a court subpoena, in any federal, state, or local civil, criminal, administrative, legislative, or other proceedings. The researchers will use the Certificate to resist any demands for information

that would identify the subject, except as explained below.

The Certificate cannot be used to resist a demand for information from personnel of the United States Government that is used for auditing or evaluation of federally funded projects, like this study, or for information that must be released in order to meet the requirements of the Federal Food and Drug Administration (FDA).

A Certificate of Confidentiality does not prevent the subject from voluntarily releasing information about themselves or their involvement in this research. If any person or agency obtains a written consent to receive research information, then the researchers may not use the Certificate to withhold that information.

The Certificate of Confidentiality does not prevent the researchers from reporting without the subject's parent(s)/guardian(s) consent, information that would identify the subject as a participant in the research project regarding matters that must be legally reported including child and elder abuse, sexual abuse, or wanting to harm themselves or others.

The release of individual private information or specimens for other research will only occur if consent was obtained from the individual's parent(s)/guardian(s) for the individual to whom the information, document, or biospecimen pertains, or for the purposes of other research that is in compliance with applicable Federal regulations governing the protection of human subjects in research.

9.6 Costs, Compensation, and Research Related Injuries

There is no cost to subjects or their parent(s)/guardian(s) for the research tests while taking part in this trial. Subjects and their parent(s)/guardian(s) may be compensated for their participation in this trial. Compensation will be in accordance with the local IRB's policies and procedures, and subject to IRB approval.

10 STATISTICAL CONSIDERATIONS

This study will enroll from participants enrolled in the main study. Based on enrollment targets for the phase 3 trial, up to 3,000 participants may be enrolled, maintaining the 2:1 (vaccine:placebo) randomization from the phase 3 study.

10.1 Sample Size Considerations

Very little data are available on incidence rates of SARS-CoV-2 infection in children age 12-17. The CDC (https://www.cdc.gov/coronavirus/2019-ncov/cases-updates/burden.html) estimates a cumulative incidence in children 5-17 years old of 27,218 per 100,000 from February 2020-December 2021 which translates into approximately 5.7 per 100,000 person-weeks. A similar incidence estimate can be calculated based on prevalence in children presenting for non-COVID-19 related hospital visits in the summer of 2020 (Sola et al, JAMA Pediatr 2021). When estimating power, we conservatively estimate that only 2,000 children enroll and complete follow-up with twice per week swabbing. The Figure below provides the power (with two-sided alpha = 0.05) to reject the null hypothesis that VE=0 under various scenarios: VEs of 0.4, 0.6 and 0.8 and attack rates equal to or 0.5 to 2 times that observed in 2020. For example, If the attack is equal to 5.7 per 100,000 person-weeks with 6 weeks of follow-up, there is 80% power to detect a VE of 60%. If the attack rate is half that from 2020 (2.9 per 100,000 person-weeks) with 6 weeks of follow-up, then the study has >80% power to detect VE of 80%. These calculations assume all infections will be detected with twice per week swabbing.

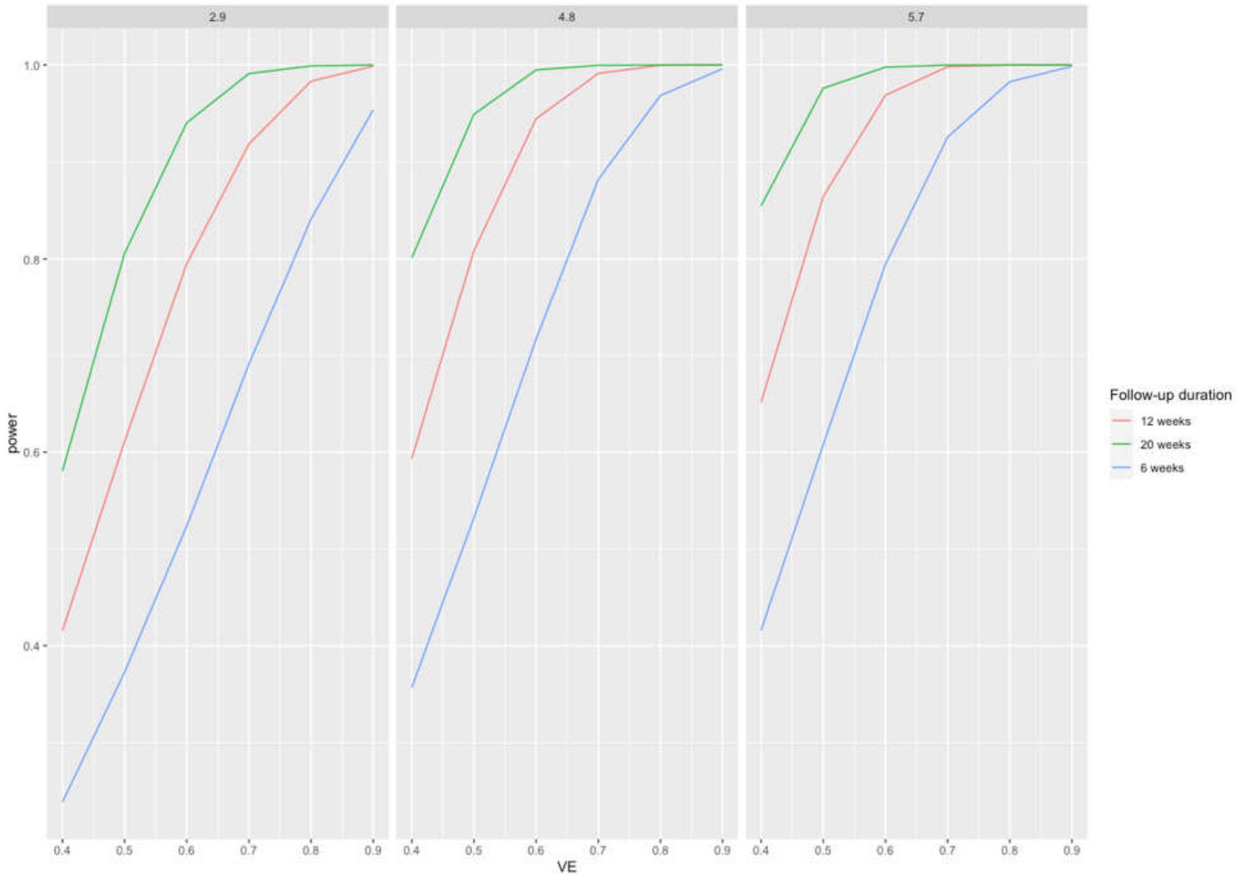

Figure 1 Power to detect vaccine efficacy against infection with 2000 participants, assuming follow-up for 6, 12 or 20 weeks and incidence rate equal to 2.9 per 100,000 person-weeks (left panel), 4.8 per 100,000 person-weeks (center panel), and 5.7 per 100,000 person-weeks (right panel).

10.2 Populations for Analysis

For analysis purposes, the following populations are defined:

Intention to Treat: All enrolled participants

Incident Infections: All participants with SARS-CoV-2 RNA detected by RNA-RT-PCR

More populations may be defined in the SAP as appropriate.

10.3 Analytic considerations

All tests will be conducted under a two-sided alpha to reject a null hypothesis that VE=0.

Primary Objective: Prevention of Infection

Endpoint: SARS-CoV-2 detected by RT-PCR on self-collected nasal swabs

Population: Intention to Treat

Incidence of SARS-CoV-2 infection will be analyzed using a Cox Proportional Hazards model with an outcome of time to first positive swab. Censoring will occur at the last negative swab for participants who never have a positive swab. The Statistical Analysis Plan will include further details including plans in the event of non-proportional hazards, differential loss to follow-up and adustment. Randomization arm will be merged from the primary study database.

Secondary Objective: Prevention of Asymptomatic/Very Mild Symptomatic Infection (A/VMS)

Endpoint: SARS-CoV-2 detected by RT-PCR on self-collected nasal swabs absent COVID-defining symptoms compatible with primary endpoint

Population: Intention to Treat

Incident infections that do not meet the COVID-19 illness endpoint definition (within a pre-defined number of days) will count towards the endpoint of A/VMS infection. Vaccine efficacy will be calculated as 1-[(number of endpoints in vaccine arm)/(number in vaccine arm)]/[(number of endpoints in placebo arm)/(number in placebo arm)]. If rates of censoring are high, either due to drop out or early cross-over, this analysis will instead be conducted using time to event analyses, such as Cox Proportional Hazards models. Details will be contained in the Statistical Analysis Plan. The analysis will be conducted on the Intention to Treat population. Data sources from the Phase 3 trial (e.g., study product assignment and clinical symptomatology,

including adjudicated COVID-19 illness endpoints) will be accessed to ascertain the symptomatology of participants who are PCR-positive in the ancillary study.

Exploratory analyses conducted on the Intention to Treat and Incident Infections populations to meet the exploratory objectives of this protocol will be detailed in the Statistical Analysis Plan.

11 SOURCE DOCUMENTS AND ACCESS TO SOURCE DATA/DOCUMENTS

Each participating site will maintain appropriate medical and research records in compliance with ICH E6, Section 4.9 and regulatory and institutional requirements for the protection of confidentiality of subjects. Each site will permit authorized representatives of the DMID, its designees, and appropriate regulatory agencies to examine (and when required by applicable law, to copy) clinical records for the purposes of quality assurance reviews, audits, and evaluation of the study safety and progress. These representatives will be permitted access to all source data and source documents, which include, but are not limited to, hospital records, clinical and office charts, laboratory notes, memoranda, subjects' memory aid or evaluation checklists, pharmacy dispensing records, recorded data from automated instruments, copies or transcriptions certified after verification as being accurate and complete, microfiches, photographic negatives, microfilm or magnetic media, x-rays, and subject files and records kept at the pharmacy, at the laboratories, and medico-technical departments involved in the clinical trial.

12 QUALITY CONTROL AND QUALITY ASSURANCE

Each participating site(s) and its subcontractors are responsible for conducting routine quality assurance (QA) and quality control (QC) activities to internally monitor study progress and protocol compliance. The site principal investigator will provide direct access to all study-related sites, source data/data collection forms, and reports for the purpose of monitoring and auditing by the sponsor, and inspection by local and regulatory authorities. The site principal investigator will ensure all study personnel are appropriately trained and applicable documentations are maintained on site.

The DCC will implement quality control procedures beginning with the data entry system and generate data quality control checks that will be run on the database. Any missing data or data anomalies will be communicated to the participating site(s) for clarification and resolution.

13 DATA HANDLING AND RECORD KEEPING

13.1 Data Management Responsibilities

The investigator is responsible to ensure the accuracy, completeness, legibility, and timeliness of the data reported. All source documents should be completed in a neat, legible manner to ensure accurate interpretation of data. Black or blue permanent ink is required to ensure clarity of reproduced copies. When making changes or corrections, cross out the original entry with a single line, and initial and date the change. DO NOT ERASE, OVERWRITE, OR USE CORRECTION FLUID OR TAPE ON THE ORIGINAL.

With the exception of the informed consent/assent documents, data will be entered directly into the electronic CRF (eCRF), which will serve as source data. If the eCRF is unavailable, paper source documents may be used and subsequently entered into the electronic database.

13.2 Data Coordinating Center/Biostatistician Responsibilities

Data collection is the responsibility of the study personnel at the participating clinical study site under the supervision of the site principal investigator. During the study, the site principal investigator must maintain complete and accurate documentation for the study.

The data coordinating center for this study will be responsible for data management, quality review, analysis, and reporting of the study data.

13.3 Data Capture Methods

Clinical (including, but not limited to, AE/SAEs) data will be collected and entered directly into eCRFs via a 21 CFR Part 11-compliant internet data entry system provided by the study data coordinating center. The data system includes password protection and internal quality checks, such as automatic range checks, to identify data that appear inconsistent, incomplete, or inaccurate. If the eCRFs are not available at a given time, paper source documents may be used and data subsequently entered into the electronic database.

13.4 Types of Data

Data for this trial will include clinical, laboratory, and contact information required for bridging to the parent study.

13.5 Study Records Retention

Study records and reports including, but not limited to, eCRFs, source documents, ICFs, and laboratory test results will be retained for 2 years after a marketing application is approved for the study product for the indication for which it is being investigated; or, if no application is to be filed or if the application is not approved for the study product, until 2 years after the investigation is discontinued and the FDA has been notified. These documents will be retained for a longer period, however, if required by local regulations. ICFs for future use will be maintained as long as the sample/specimen exists.

No records will be destroyed without the written consent of the sponsor. It is the responsibility of the sponsor to inform the site principal investigator when these documents no longer need to be retained. The participating VTEU sites must contact DMID for authorization prior to the destruction of any study records.

14 CLINICAL MONITORING

Clinical site monitoring is conducted to ensure that the rights and well-being of trial subjects are protected and that the reported trial data are accurate, complete, and verifiable. Clinical monitoring also ensures that conduct of the trial is in compliance with the currently approved protocol/ amendment(s), ICH, GCP, and with applicable regulatory requirement(s) and Sponsor requirements. Clinical monitoring will also verify that any critical study procedures are completed following specific instructions in the protocol-specific guidance.

Monitoring for this study will be performed by DMID or their designee. Details of clinical site monitoring are documented in a clinical monitoring plan (CMP). The CMP describes in detail who will conduct the monitoring, at what frequency monitoring will be done, at what level of detail monitoring will be performed, and the distribution of monitoring reports. Monitoring visits will include, but are not limited to, review of regulatory files, CRFs, ICFs, medical and laboratory reports, training records, and protocol and GCP compliance. Site monitors will have access to each participating site, study personnel, and all study documentation according to the DMID-approved site monitoring plan. Study monitors will meet with site PIs to discuss any problems and outstanding issues and will document site visit findings and discussions. Remote site monitoring may also occur.

15 PUBLICATION POLICY

Following completion of the study, the lead Principal Investigator is expected to publish the results of this research in a scientific journal. All investigators funded by the NIH must submit or have submitted for them to the National Library of Medicine's PubMed Central (http://www.ncbi.nlm.nih.gov/pmc/) an electronic version of their final, peer-reviewed manuscripts upon acceptance for publication, to be made publicly available no later than 12 months after the official date of publication. The NIH Public Access Policy ensures the public has access to the published results of NIH funded research. It requires investigators to submit final peer-reviewed journal manuscripts that arise from NIH funds to the digital archive PubMed Central upon acceptance for publication. Further, the policy stipulates that these papers must be accessible to the public on PubMed Central no later than 12 months after publication.

Refer to:

- NIH Public Access Policy, http://publicaccess.nih.gov/
- NIH Office of Extramural Research (OER) Grants and Funding, http://grants.nih.gov/grants/oer.htm

As of January 2018, all clinical trials supported by the NIH must be registered on ClinicalTrials.gov, no later than 21 days after the enrollment of the first subject. Results of all clinical trials supported by the NIH, generally, need to be submitted no later than 12 months following the primary completion date. A delay of up to 2 years is available for trials that meet certain criteria and have applied for certification of delayed posting.

As part of the result posting a copy of this protocol (and its amendments) and a copy of the Statistical Analysis Plan will be posted on ClincialTrials.gov.

For this trial the responsible party is University of Maryland School of Medicine, Center for Vaccine Development and Global Health, which will register the trial and post results.

The responsible party does not plan to request certification of delayed posting.

Refer to:

- Public Law 110-85, Section 801, Clinical Trial Databases
- 42CFR11
- NIH NOT-OD-16-149

16 LITERATURE REFERENCES

Sola AM, David AP, Rosbe KW, Baba A, Ramirez-Avila L, Chan DK. Prevalence of SARS-CoV-2 Infection in Children Without Symptoms of Coronavirus Disease 2019. JAMA Pediatr. 2021 Feb 1;175(2):198-201

17 APPENDIX

Appendix A. Schedule of Events

Procedure	Enrollment: D21+ of parent study	Home swab self-collection phase: participants will self-collect a nasal swab twice weekly during the following weeks of enrollment in the ancillary swab study*																											EOS **
	Day 0	1	2	3	4	5	6	7	8	9	10	11	12	13	14	15	16	17	18	19	20	21	22	23	24	25	26	27	28
Informed consent	X																												
Eligibility criteria	X																												
Twice weekly nasal swabs	X	X	X	X	X	X	X	X	X	X	X	X	X	X	X	X	X	X	X	X	X	X	X	X	X	X	X	X	X

EOS: End of study December 17, 2021

* The number of weeks that participants will collect two swabs is only an estimate and could range from approximately 6 to 20 weeks depending on when the participant is enrolled and when the cross-over study occurs.

**EOS: End of study; occurs when a swab is collected at the time of the second cross-over injection of the parent Phase 3 study.

Appendix B. Protocol Change History

Location of change	Change/modification in Version 4.0
Protocol summary/Design of the Study	Updated duration of sampling to 16-28 weeks.
Protocol summary/Study Objectives	Added 2 additional secondary endpoints:<br>• To determine the durability of efficacy of SARS-CoV-2 rS/M1 vaccine against infection<br>• To determine the durability of efficacy of SARS-CoV-2 rS/M1 vaccine against asymptomatic/very mildly symptomatic infection
Protocol summary/Duration of Individual Subject Participation	Updated study duration to approximately 4-7 months.
2.1 Background	Added paragraphs on the importance of assessing durability of protection and describing the blinded cross-over.
3.1 Study Design Description	Added paragraph describing the extension of swab collection to allow assessment of duration of vaccine efficacy.
3.2 Study Objectives/3.2.2 Secondary	Added 2 additional secondary endpoints:<br>• To determine the durability of efficacy of SARS-CoV-2 rS/M1 vaccine against infection<br>• To determine the durability of efficacy of SARS-CoV-2 rS/M1 vaccine against asymptomatic/very mildly symptomatic infection

3.3 Study Endpoints or Outcome Measures/3.3.2 Secondary	Added 2 additional secondary endpoints:<br>• RT-PCR-confirmed SARS-CoV-2 infection detected in nasal swabs collected twice weekly from the crossover dose 2 visit (or thereafter) until ancillary study end among the group who initially received vaccine compared to the group who initially received placebo<br>• RT-PCR-confirmed SARS-CoV-2 infection detected in nasal swabs collected twice weekly from the crossover dose 2 visit (or thereafter) until ancillary study end among the group who initially received vaccine compared to the group who initially received placebo without COVID-19 illness as defined in the primary Phase 3 study endpoint
6.3 Twice weekly swab self-collection at home	Updated approximate duration of participation to 16-28 weeks.
6.5 Final Study Visit	Updated final swab collection to be December 17, 2021.
17 Appendix	Updated Schedule of Events to reflect new EoS.
**Location of change**	**Change/Modification in Version 3.0**
Statement of Compliance, bullet 3	Updated wording to be consistent with October 2015 guidelines

Section 2.3.1 Potential Risks, Section 6.3 Twice Weekly Swab Collection at Home, Section 7.1.1 Research Assays, 8.1.1 Adverse Events (AEs) Reporting	Corrected “mid-turbinate” to “anterior nares”.
Section 3.1 Study Design Description, Section 6.5 Final Study Visit	Clarified End of Study will occur at the second dose of the blinded cross-over.
Section 6.2 Enrollment	Added information on provisional devices to be provided to participants who do not have access to their own Wi-Fi/camera-enabled device.
Section 6.3 Twice Weekly Swab Collection at Home	Clarified that swab collection should occur as soon as possible on or after day 21 of the parent study.
Section 5.1.1 Subject Inclusion Criteria	Updated wording in inclusion criteria 6 and 7 to reflect allowable devices and need for unique email address.

Protocol Changes from Original (Version 1.0)

to Final (Version 4.0)

Protocol 21-0011 Summary of changes version 2.0 (22 April 2021)

**Location of Change**	**Change/Modification in Version 2.0**
Section 3.1 Study Design Description	Cut description of potential conditions that might allow for cross-over to reflect updated primary study (PREVENT-19) commitment to cross-over.
Section 6.1 Screening	Cut text describing potential tele-health enrollment visit.
Section 6.3 Twice Weekly Swab Self-collection at Home	Added description of reminder emails and phone calls to improve retention
Section 7.1.1 Research Assays	Cut text to remove discussion of possible remote enrollment
Section 8.1.1 Adverse Events (AEs) Reporting	Added "Only events that are moderate or severe in intensity will be recorded."
Section 9.2 Informed Consent Process	Removed potential for recruitment prior to study consent.
Section 13.1 Data Management Responsibilities	Added allowance for the use of paper source documents if the eCRF is temporarily unavailable, with subsequent data entry into the electronic database.
Section 13.4 Types of Data	Added "laboratory" to account for laboratory data in addition to clinical and contact information for bridging to parent study.
Section 14 Clinical Monitoring	Added potential for remote site monitoring.

Protocol 21-0011 Summary of changes version 3.0 (17 June 2021)

**Location of change**	**Change/Modification in Version 3.0**
Statement of Compliance, bullet 3	Updated wording to be consistent with October 2015 guidelines
Section 2.3.1 Potential Risks, Section 6.3 Twice Weekly Swab Collection at Home, Section 7.1.1 Research Assays, 8.1.1 Adverse Events (AEs) Reporting	Corrected "mid-turbinate" to "anterior nares".
Section 3.1 Study Design Description,<br>Section 6.5 Final Study Visit	Clarified End of Study will occur at the second dose of the blinded cross-over.
Section 6.2 Enrollment	Added information on provisional devices to be provided to participants who do not have access to their own WiFi/camera-enabled device.

Section 6.3 Twice Weekly Swab Collection at Home	Clarified that swab collection should occur as soon as possible on or after day 21 of the parent study.
Section 5.1.1 Subject Inclusion Criteria	Updated wording in inclusion criteria 6 and 7 to reflect allowable devices and need for unique email address.

Protocol 21-0011 Summary of changes version 4.0 (20 Aug 2021) – Final version

Location of change	Change/modification in Version 4.0 – Final version
Protocol summary/Design of the Study	Updated duration of sampling to 16-28 weeks.
Protocol summary/Study Objectives	Added 2 additional secondary endpoints:• To determine the durability of efficacy of SARS-CoV-2rS/M1 vaccine against infection• To determine the durability of efficacy of SARS-CoV-2rS/M1 vaccine against asymptomatic/very mildlysymptomatic infection
Protocol summary/Duration of Individual Subject Participation	Updated study duration to approximately 4-7 months.
2.1 Background	Added paragraphs on the importance of assessing durability of protection and describing the blinded cross-over.
3.1 Study Design Description	Added paragraph describing the extension of swab collection to allow assessment of duration of vaccine efficacy.
3.2 Study Objectives/ 3.2.2 Secondary	Added 2 additional secondary endpoints:• To determine the durability of efficacy of SARS-CoV-2rS/M1 vaccine against infection• To determine the durability of efficacy of SARS-CoV-2rS/M1 vaccine against asymptomatic/very mildly symptomatic infection
3.3 Study Endpoints or Outcome Measures/ 3.3.2 Secondary	Added 2 additional secondary endpoints:<br>• RT-PCR-confirmed SARS-CoV-2 infection detected in nasal swabs collected twice weekly from the cross-over dose 2 visit (or thereafter) until ancillary study end among the group who initially received vaccine compared to the group who initially received placebo<br>• RT-PCR-confirmed SARS-CoV-2 infection detected in nasal swabs collected twice weekly from the cross-over dose 2 visit (or thereafter) until ancillary study end among the group who initially received vaccine compared to the group who initially received placebo without COVID-19 illness as defined in the primary Phase 3 study endpoint
6.3 Twice weekly swab self-collection at home	Updated approximate duration of participation to 16-28 weeks.
6.5 Final Study Visit	Updated final swab collection to be December 17, 2021.
17 Appendix	Updated Schedule of Events to reflect new EoS.

Original Statistical Analysis Plan Version 1.0

STATISTICAL ANALYSIS PLAN

A nasal swab study to assess the efficacy of vaccination in the prevention of SARS-CoV-2 infection among individuals enrolled in a Phase 3 efficacy trial of a SARS-CoV-2 recombinant spike protein (rS) vaccine with Matrix-M1™ (M1) adjuvant

DMID Protocol Number: 21-0011
CoVPN Number: 3004-01

Effective Date: 21/04/2022

Version: 1.0

All proprietary rights reserved

STATISTICAL ANALYSIS PLAN

**Protocol Name:**	A nasal swab study to assess the efficacy of vaccination in the prevention of SARS-CoV-2 infection among individuals enrolled in a Phase 3 efficacy trial of a SARS-CoV-2 recombinant spike protein (rS) vaccine with Matrix-M1™ (M1) adjuvant
**Protocol Number:**	**DMID Protocol Number:** 21-0011
**Author(s):**	Elizabeth R. Brown, ScD<br>Yuqing Jiao, MS
**Version:**	*1.0*

**Author(s):**

Elizabeth Brown, ScD	*See appended approval*	*See appended approval*
Lead Statistician	Signature	Date: 21/04/2022
Yuqing Jiao	*See appended approval*	*See appended approval*
Statistical Research Associate	Signature	Date: 21/04/2022

All proprietary rights reserved

TABLE OF CONTENTS

1. LIST OF ABBREVIATIONS, ACRONYMS AND DEFINITIONS 5
2. INTRODUCTION 5
2.1 GENERAL DESIGN CONSIDERATIONS 5
2.2 STUDY OBJECTIVES AND ENDPOINTS 6
2.3 RANDOMIZATION 7
2.4 BLINDING 8
2.5 SAMPLE SIZE AND POWER 8
3. GENERAL DATA ANALYSIS CONSIDERATIONS 9
3.1 ANALYSIS SET(S) 9
3.2 STATISTICAL ANALYSIS ISSUES 9
3.3 INCORRECT/MISSING SWABBING DATE 10
4. INTERIM ANALYSIS AND DATA MONITORING COMMITTEE 11
5. GENERAL ANALYSIS METHODS 11
6. TRIAL PARTICIPANT DISPOSITION 11
6.1 DISPOSITION OF PARTICIPANT 11
6.1.1 Screening and Enrollment 11
6.1.2 Compliance to Swabbing 11
6.1.3 Study Discontinuation 12
6.2 TREATMENT EXPOSURE 12
7. BASELINE DATA 12
8. SAFETY ANALYSES 12
9. PRIMARY EFFICACY/EFFECTIVENESS ANALYSES 13
9.1 DESCRIPTIVE MEASURES OF SARS-COV-2 ACQUISITION: 13
9.2 PRIMARY OBJECTIVE: PREVENTION OF INFECTION 13
10. SECONDARY ENDPOINTS 15
10.1 SECONDARY OBJECTIVE: PREVENTION OF ASYMPTOMATIC/VERY MILD SYMPTOMATIC INFECTION (A/VMS) 15
10.2 SECONDARY OBJECTIVE: TO DETERMINE THE DURABILITY OF EFFICACY OF SARS-COV-2 RS/M1 VACCINE AGAINST INFECTION 15
10.3 SECONDARY OBJECTIVE: TO DETERMINE THE DURABILITY OF EFFICACY OF SARS-COV-2 RS/M1 VACCINE AGAINST ASYMPTOMATIC/VERY MILDLY SYMPTOMATIC INFECTION 15
11. EXPLORATORY ENDPOINTS 15
11.1 EXPLORATORY OBJECTIVE: TO DETERMINE THE SEQUENCE OF BREAKTHROUGH SARS-COV-2 INFECTIONS 15
11.2 EXPLORATORY OBJECTIVE: TO ESTIMATE THE VE ON DURATION OF INFECTION 16

All proprietary rights reserved

11.3 Exploratory Objective: To estimate the VE against SARS-CoV-2 viral load as a proxy of transmission 16
11.4 Exploratory Objective: To estimate the efficacy of SARS-CoV-2 rS/M1 vaccine against a composite outcome of asymptomatic/very mildly symptomatic infection plus pre-symptomatic infection 16
11.5 Exploratory Objective: To explore whether observed VE against COVID-19 illness reflects a transition from symptomatic into asymptomatic infections versus an absolute reduction in all SARS-CoV-2 infections 16

12. ADDITIONAL PLANNED ANALYSES 16

13. REFERENCES 17

14. CHANGE HISTORY 17

1. LIST OF ABBREVIATIONS, ACRONYMS AND DEFINITIONS

Term/Abbreviation	Definition
IDCRC	Infectious Disease Clinical Research Consortium
SDSU	Statistical and Data Sciences Unit
parent study	A Phase 3, Randomized, Observer-Blinded, Placebo-Controlled Study to Evaluate the Efficacy, Safety, and Immunogenicity of a SARS-CoV-2 Recombinant Spike Protein Nanoparticle Vaccine (SARS-CoV-2 rS) with Matrix-M1™ Adjuvant in Adult Participants ⩾ 18 Years with a Pediatric Expansion in Adolescents (12 to 17 Years). Protocol 2019nCoV-301
Initial series	The series of two injections of either vaccine or placebo given on days 0 and 21
Crossover series	The second series of two injections of either vaccine or placebo given at crossover and 21 days later

2. INTRODUCTION

This SAP covers the final analysis of data collected in the SNIFF Study, a substudy of adolescents aged 12-17 enrolled in the Novavax study (the parent study).

2.1 General Design Considerations

**Short Title:**	SNIFF
**Design of the Study:**	In this ancillary swab study, up to 3,000 adolescent participants 12-17 years of age in the Phase 3 SARS-CoV-2 vaccine trial (PREVENT-19) sponsored by Novavax will be sampled twice weekly for approximately 16-28 weeks with nasal swabs for detection of SARS-CoV-2 infection by RT-PCR and incident infections tallied.
**Study Phase:**	Observational
**Study Population:**	Subset of participants in Protocol 2019nCoV-301, entitled "A Phase 3, Randomized, Observer-Blinded, Placebo-Controlled Study to Evaluate the Efficacy, Safety, and Immunogenicity of a SARS-CoV-2 Recombinant Spike Protein Nanoparticle Vaccine (SARS-CoV-2 rS) with Matrix-M1™ Adjuvant in Adult Participants ≥ 18 Years with a Pediatric Expansion to adolescents (12 to 17 years)" (PREVENT-19)
**Study Duration**	Follow-up is expected to be completed December 2021. Total duration of the study will be approximately 6 months from first enrolment.
**Number of Sites:**	Up to 75 sites located in the US

**Description of Study Product or Intervention:**	Participants in the PREVENT-19 Phase 3 trial (pediatric expansion study) are randomized to receive either SARS-CoV-2 rS/M1 vaccine or placebo (2:1 allocation) on days 0 and 21. The pediatric expansion study will have a "blinded-crossover" after ~3 months of follow-up after the completion of the initial set of vaccinations. Pediatric participants will be scheduled for administration of 2 injections of the alternate study material 21 days apart. In the current protocol, no interventional product will be administered.

2.2 Study Objectives and Endpoints

Objectives	Endpoints
Primary	
• To estimate the efficacy of SARS-CoV-2 rS/M1 vaccine against infection	• Reverse transcriptase polymerase chain reaction (RT-PCR)-confirmed SARS-CoV-2 infection detected in nasal swabs collected twice weekly from the day 21 visit or thereafter, until the second dose in the second vaccination (crossover) series in the vaccine group and the placebo group.
Secondary	
• To estimate the efficacy of SARS-CoV-2 rS/M1 vaccine against asymptomatic/ very mildly symptomatic infection	• RT-PCR-confirmed SARS-CoV-2 infection detected in nasal swabs collected twice weekly from the day 21 visit or thereafter, until the second dose in the second vaccination (crossover) series, without COVID-19 illness as defined in the primary Phase 3 study endpoint. The COVID-19 disease endpoint will be defined in the main study database.
• To determine the durability of efficacy of SARS-CoV-2 rS/M1 vaccine against infection	• RT-PCR-confirmed SARS-CoV-2 infection detected in nasal swabs collected twice weekly from the crossover dose 2 visit (or thereafter) until ancillary study end among the group who initially received vaccine compared to the group who initially received placebo

All proprietary rights reserved

• To determine the durability of efficacy of SARS-CoV-2 rS/M1 vaccine against asymptomatic/very mildly symptomatic infection	• RT-PCR-confirmed SARS-CoV-2 infection detected in nasal swabs collected twice weekly from the crossover dose 2 visit (or thereafter) until the second dose in the second vaccination (crossover) series among the group who initially received vaccine compared to the group who initially received placebo without COVID-19 illness as defined in the primary Phase 3 study endpoint. The COVID-19 disease endpoint will be defined in the main study database.
Exploratory	
• To determine the sequence of breakthrough SARS-CoV-2 infections*	• Viral genome sequence of RT-PCR-positive samples
• To estimate the VE on duration of infection	• Duration of shedding as indicated by RT-PCR in vaccinated vs placebo recipients
• To estimate the VE against SARS-CoV-2 viral load as a proxy of transmission	• Reduction in viral load (and perhaps subgenomic RNA), as a proxy for transmission, in RT-PCR-positive samples from vaccinated vs placebo recipients
• To estimate the efficacy of SARS-CoV-2 rS/M1 vaccine against a composite outcome of asymptomatic/very mildly symptomatic infection plus pre-symptomatic infection	• Proportion of days that are symptom-free on days that RT-PCR-positive swabs are collected
• To explore whether observed VE against COVID-19 illness reflects a transition from symptomatic into asymptomatic infections versus an absolute reduction in all SARS-CoV-2 infections	• Ratio of asymptomatic to symptomatic infections in vaccinated vs placebo recipients. Data on symptomatology will be extracted from the Phase 3 trial database, which will be separate but linkable to the ancillary

*This document will provide analytical plans for all but the first exploratory endpoint.

2.3 Randomization

Randomization is part of the parent study. All details are included in that protocol.

2.4 Blinding

The blinding codes are not maintained or accessible by SCHARP staff or any members of the IDCRC SDSU and will only be provided to SCHARP after the lock of the database for this study.

2.5 Sample Size and Power

Very little data are available on incidence rates of SARS-CoV-2 infection in children aged 12-17. The CDC (https://www.cdc.gov/coronavirus/2019-ncov/cases-updates/burden.html) estimates a cumulative incidence in children 5-17 years old of 27,218 per 100,000 from February 2020-December 2021 which translates into approximately 5.7 per 1,000 person-weeks. A similar incidence estimate can be calculated based on prevalence in children presenting for non-COVID-19 related hospital visits in the summer of 2020 (Sola et al, JAMA Pediatr 2021). When estimating power for the protocol, we estimated that 2,000 children would enroll and complete follow-up with twice per week swabbing. Figure 1 provides the power (with two-sided alpha = 0.05) to reject the null hypothesis that VE=0 under various scenarios: VEs of 0.4, 0.6 and 0.8 and attack rates equal to or 0.5 to 2 times that observed in 2020. For example, if the attack is equal to 5.7 per 1,000 person-weeks with 6 weeks of follow-up, there is 80% power to detect a VE of 60%. If the attack rate is half that from 2020 (2.9 per 1,000 person-weeks) with 6 weeks of follow-up, then the study has >80% power to detect VE of 80%. These calculations assume all infections will be detected with twice per week swabbing.

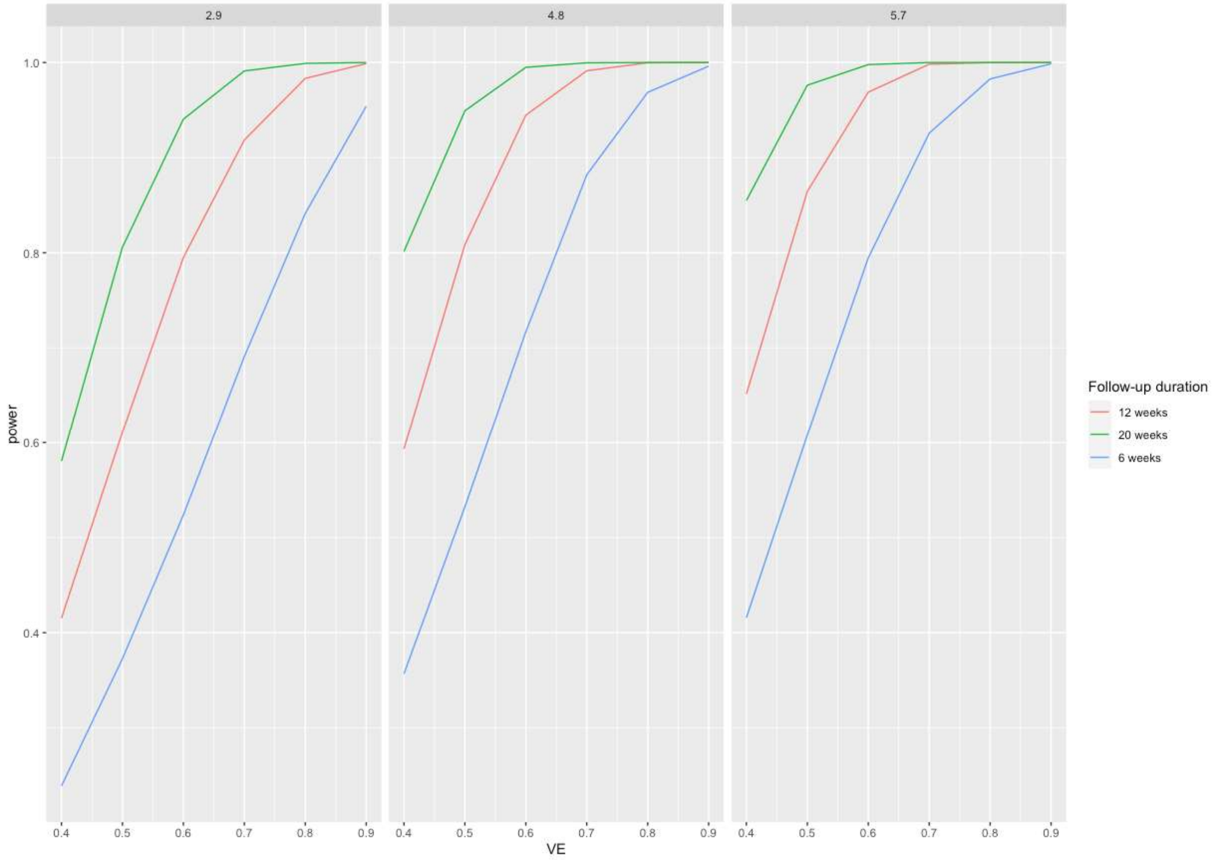

Figure 1 Power to detect vaccine efficacy against infection with 2000 participants, assuming follow-up for 6, 12 or 20 weeks and incidence rate equal to 2.9 per 1,000 person-weeks (left panel), 4.8 per 1,000 person-weeks (center panel), and 5.7 per 1,000 person-weeks.

3. GENERAL DATA ANALYSIS CONSIDERATIONS

3.1 Analysis Set(s)

For analysis purposes, the following populations are defined:

Intention to Treat (ITT): All enrolled participants

Modified Intention to Treat (mITT): All enrolled participants who were not identified as having SARS-CoV-2 infection, either through RT-PCR or serological testing, prior to administration of the second dose of the first dosing series.

Primary analysis set (PAS): The intention of the SNIFF protocol was to enroll participants at their second dose of the first vaccination series and immediately start swabbing. This would allow exclusion of all participants who were previously infected. However, many participants enrolled after the second dose of the initial series, and it may be impossible to determine if they acquired infection in between that second dose and the first swab. We define this data set to account for this by limiting inclusion to mITT participants enrolled before 4 weeks post second dose of the initial series. Primary analyses will be conducted on this data set.

Primary analyses set 2 (PAS2): All mITT participants enrolled by July 12, 2021. Primary analyses will be conducted on this data set. This analysis set serves as a complement to PAS in case too many participants have enrolled after 4 weeks post second dose of the primary series. 82.4% were enrolled by this date.

Post crossover analysis set (PCAS): All mITT participants enrolled before 4 weeks post second dose of the crossover series without evidence of prior infection before or at the second dose of the crossover series.

PCR-positive (PP-ITT): All participants in the ITT population with SARS-CoV-2 RNA detected by RNA-RT-PCR as part of the SNIFF study (as determined by the lab).

PCR-positive PAS (PP-PAS): All participants in the PAS with SARS-CoV-2 RNA detected by RNA-RT-PCR as part of the SNIFF study (as determined by the lab).

PCR-positive PAS (PP-PAS2): All participants in the PAS2 with SARS-CoV-2 RNA detected by RNA-RT-PCR as part of the SNIFF study (as determined by the lab).

3.2 Statistical Analysis Issues

Key information such as product assignment (randomization), clinical symptomatology, adjudicated COVID-19 illness endpoints, RNA PCR results, demographics, risk factors, timing of vaccination, outside vaccination receipt date, and laboratory results (specifically seroconversion indicative of prior infection) are collected in the parent protocol. Those data will be sent to SCHARP after the study ends to merge with the SNIFF data for analyses.

This document was written without knowledge of the primary study database or access to any data summaries from the parent study, including enrollment characteristics of the population, and may need to be updated once this database is provided to the statistical team.

SARS-CoV-2 Viral Load

Viral load assessment will be conducted via digital PCR methods using N1 and N3 target sets and expressed in copies/swab on a per sample basis for all specimens with a "detected" interpretation.

Change of predominant variant in the United States during follow-up time

From the first enrollment in this study to the end of follow-up, the predominant SARS-Cov-2 strain changed from alpha to delta (Figure 2). We will plan to repeat analyses in the subset of sites in states where delta was the most prevalent strain (>80%) at the start of enrollment (according to nextstrain.org).

Durability analysis

This SAP reflects a version of the protocol in which durability was included as a secondary endpoint. Due to the changing prevalence of variants throughout the follow-up time and the expedited crossover timelines in the parent study, it will be challenging to estimate durability. First, the follow-up time for the group vaccinated early may be too short to expect a meaningful decline in efficacy. Second, durability estimates will be confounded by variant (see Figure 2).

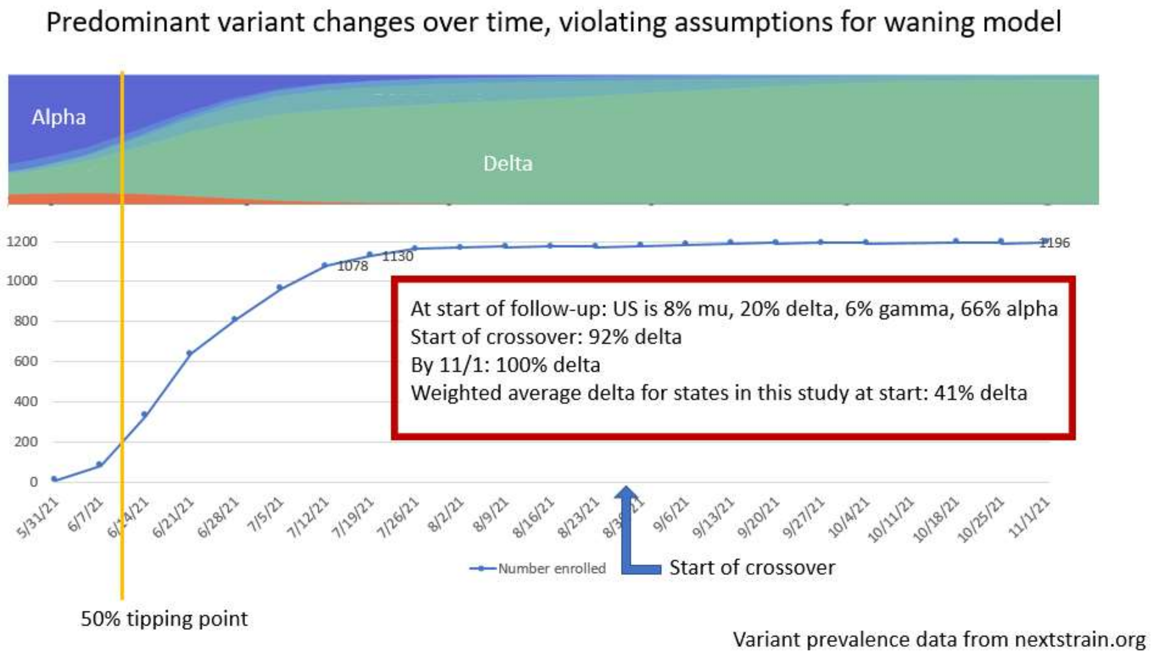

Figure 2 Enrollment into SNIFF and prevalence of predominant variants in the United States over calendar time. Data on prevalence of variants is provided by nextstrain.org, accessed on 11/30/2021.

3.3 Incorrect/missing Swabbing Date

At times in the study, participants neglected to scan their swabs before returning them or scanned multiple swabs on the same date. Cases with evidence of an incident infection, either by receipt of a

swab with RNA detected by PCR, or evidence of infection from the parent study, will be reviewed by an adjudication committee to determine timing of infection.

4. INTERIM ANALYSIS AND DATA MONITORING COMMITTEE

No interim analyses were planned for this study nor was there a data monitoring committee.

5. GENERAL ANALYSIS METHODS

Descriptive statistics that will be used to summarize continuous variables are as follows: mean and standard deviation, median and interquartile range, quartiles, range, and number of missing data values. For categorical variables, descriptive statistics that will be used include the following: frequencies, relative frequencies, and the number of missing data values. Descriptive analyses summarizing baseline and follow-up data will be stratified by state. Line, scatter, and box plots will be used, as appropriate, for longitudinal data representations. Covariates from regression models will be assessed using Wald tests and corresponding 95% confidence intervals, unless otherwise specified. A two-sided alpha level of 0.05 will be used for all statistical tests, unless otherwise specified.

6. TRIAL PARTICIPANT DISPOSITION

6.1 Disposition of Participant

6.1.1 Screening and Enrollment

Dates of site activation, first enrollment, and last enrollment, as well as the number of participants screened, number and percentage of participants enrolled, and the screening-to-enrollment ratio will be displayed in a table overall and by site and randomization arm.

The number of participants included in each data set will be tabulated by site and randomization arm.

Reasons for participants who are not eligible for the study will be displayed in a listing.

6.1.2 Compliance to Swabbing

Participants are expected to swab on Mondays and Thursdays. Thus the weeks are separated into two collection windows – window1 (Monday, Tuesday, Wednesday) and window 2 (Thursday, Friday, Saturday, Sunday). Alluvial plots will be used to capture completeness of swabbing. Categories: 0 windows, 1 window, or 2 windows per week, discontinued (exited study). These will be created to describe the population overall and by randomization arm.

The number of expected swabs versus resulted scanned swabs will be tabulated overall and by site and randomization arm.

All proprietary rights reserved

6.1.3 Study Discontinuation

The number and percentages of participants who completed the study, as well as the reasons for non-completion, will be displayed in a table overall and by site and by randomization arm and by follow-up period.
Time from enrollment to study discontinuation and time from crossover to study discontinuation will be summarized using Kaplan-Meier curves overall and by arm.

6.2 Treatment Exposure

Treatment exposure will be defined by randomization arm from the parent protocol and number of doses received. The number and percentage of participants who received no doses, one dose, and two doses will be displayed in a table overall and by site and randomization arm.

7. BASELINE DATA

Baseline demographic characteristics of the participants, such as site, state, age, sex, randomization arm, race, exposure risk information such as whether "the subject is a student attending school in person" will be displayed in a table overall and by site, and in a table overall and by randomization arm. Baseline is defined as time of enrollment into SNIFF. Demographic characteristics are collected in the parent study with a collection time that may not align with enrollment into SNIFF; therefore, baseline characteristics will be determined from the data collected most recently before enrollment into SNIFF.

The set of characteristics collected at baseline (including randomization arm) in the parent study will be compared between those who enrolled in SNIFF versus those who did not.

8. SAFETY ANALYSES

Limited safety data were collected as part of the SNIFF study and are expected to be low in frequency. If applicable, summaries of adverse events will include a cumulative listing of adverse events and a table displaying the total number of adverse events by severity grade and relationship to study product.

All proprietary rights reserved

9. PRIMARY EFFICACY/EFFECTIVENESS ANALYSES

9.1 Descriptive measures of SARS-CoV-2 acquisition:

Table 1 Table for use when summarizing incident outcomes, including infection, overall and with and without COVID-defining symptoms.

Randomization arm	Exposure	Number of participants	Number of incident endpoints	Person-years	Incidence rate (95% CI)
Vaccine -> Placebo	Two doses of vaccine			See note 2	
Placebo -> Vaccine	Single dose of vaccine			See note 1	
	Two doses of vaccine			See note 2	
	Unvaccinated (two placebo doses)			See note 3	

Note 1: Total number of person-years post first dose and pre first of second dose in the crossover period, infection (maybe due to RT-PCR or antibody evidence of infection as per endpoints in analyses described below) or censoring.
Note 2: Total number of person-years post second dose and pre first of infection (maybe due to RT-PCR or antibody evidence of infection as per endpoints in analyses described below) or censoring.
Note 3: Total number of person-years post second dose of placebo and pre first of infection (maybe due to RT-PCR or antibody evidence of infection as per endpoints in analyses described below) or censoring.

The table above will be replicated by time periods, stratifying by calendar month as well as pre and post crossover. Results will be plotted over time. Additional calendar periods may be defined by predominant variant at the site with further stratification by the variant associated with the endpoint, recognizing that not all infections have enough virus present to be sequenced.

Descriptive Plots
Kaplan-Meier curves will be used to display incidence of infection across study groups. Multiple curves will be produced, corresponding to the four exposure groups in Table 1 for the PAS, PAS2 and PCAS. Endpoint definitions follow in the analysis descriptions for primary and secondary analyses.

Each of these sets of plots will be produced once where the time scale is calendar time and once where the time scale is study time (time since enrollment).

9.2 Primary Objective: Prevention of Infection

Primary analysis
Endpoint: SARS-CoV-2 detected by RT-PCR on self-collected SNIFF-provided nasal swabs, right-censored at the last negative test at or before the earliest of the second dose of the crossover vaccination series (vaccine or placebo), 4 weeks post first crossover dose (applicable for cases where the second dose in the crossover period is missed or late), termination, or receipt of EUA vaccine.
Data sets: Primary analyses set; supported by analyses on the mITT set

Approach: Incidence of SARS-CoV-2 infection will be analyzed using a Cox Proportional Hazards model using follow-up time through 4 weeks post crossover. Calendar time will not be accounted for in the primary analysis since enrollment was completed in a short window of time. This analysis only counts infections until the second dose of the crossover series. Follow-up time may be right-censored earlier if a participant receives an EUA vaccine or otherwise is terminated early from the study. Randomization arm will be merged from the primary study database. Efficacy will be estimated as 1-HR (hazard ratio) from a Cox proportional hazards model. The primary analysis will not use any endpoint information collected in the parent study. This analysis will only include randomization as a covariate. The proportional hazards assumption will be assessed graphically.

Sensitivity analysis 1

The analysis will be repeated counting follow-up time up to 2 weeks post first dose of the crossover series.

Endpoint: SARS-CoV-2 detected by RT-PCR on self-collected SNIFF-provided nasal swabs, right-censored at the last negative test at or before the earliest of the second dose of the crossover series (vaccine or placebo), 2 weeks post first crossover dose (applicable for cases where the second dose is missed or late), termination, or receipt of EUA vaccine.

Supporting analysis 1

If any participants are identified as having infection in the parent study but not in the SNIFF study, the primary analysis will be repeated by revising the primary endpoint to include the first time to SARS-CoV-2 infection as defined in the parent study or SNIFF.

Endpoint: SARS-CoV-2 detected by RT-PCR on self-collected SNIFF-provided or Novavax-provided nasal swabs, right-censored at the collection time of the last RT-PCR negative swab at or before the earliest of the second dose of the crossover series (vaccine or placebo), 2 weeks post first crossover dose (applicable for cases where the second dose is missed or late), termination, or receipt of EUA vaccine. Note: Novavax-provided swabs may either be self-collected at home or collected at study visits.

Supporting analysis 2

Because the SNIFF population is a subset of the parent study and was selected post-randomization from the parent study, the primary analysis and supporting analysis 1 will be repeated with adjustment for potential confounders. These will be selected from baseline variables that are univariably (adjusted for state) predictive of risk of acquisition of infection or differ by randomization arm with $p<0.10$. If there are inadequate numbers of participants per state, state may be dropped as an adjustment variable.

Supporting analysis 3: Delta variant analysis

Supporting analysis 2 will be repeated with each person’s follow-up time starting at the time at which delta variant became over 80% predominant in their state according to nextstrain.org. Adjustment for covariates will be included as per supporting analysis 2.

All proprietary rights reserved

10. SECONDARY ENDPOINTS

10.1 Secondary Objective: Prevention of Asymptomatic/Very Mild Symptomatic Infection (A/VMS)

Endpoint: SARS-CoV-2 detected by RT-PCR on self-collected SNIFF-provided nasal swabs, right-censored at the last negative test at or before the earliest of: a COVID-defining endpoint from the parent trial, 4 weeks post first crossover dose, termination, or receipt of EUA vaccine.
Data sets: Primary analyses set; supported by analyses on the mITT set
Approach: We will use the approach presented for the primary outcome to ensure proper accounting for variable follow-up time due to early crossover. Data sources from the Phase 3 trial (e.g., study product assignment and clinical symptomatology, including adjudicated COVID-19 illness endpoints) will be accessed to ascertain the symptomatology of participants who are RT-PCR-positive in the ancillary study.

Table 1 in Section 9.1 will also be completed for this endpoint.

10.2 Secondary Objective: To determine the durability of efficacy of SARS-CoV-2 rS/M1 vaccine against infection

Endpoint: SARS-CoV-2 detected by RT-PCR on self-collected SNIFF-provided nasal swabs, right-censored at the last negative RT-PCR test at or before the earliest of termination or receipt of EUA vaccine.
Data set: Post crossover analyses set (PCAS)
Approach: The analyses for the primary endpoint will be repeated with the time origin being time of second dose (placebo or active) in the crossover series. Durability will be assessed through a time-invariant hazard ratio with supporting analyses investigating evidence of non-proportionality of the hazards that could indicate loss of efficacy in the early vaccination arm. The study may not be adequately powered for this analysis. Evidence will also be supported by the data that will be presented in the mock table (Table 1 in this document) which will examine incidence rates over calendar time in both randomization arms.

If there is adequate power, the supporting analysis for the primary objective will be repeated for this objective.

10.3 Secondary Objective: To determine the durability of efficacy of SARS-CoV-2 rS/M1 vaccine against asymptomatic/very mildly symptomatic infection

Endpoint: SARS-CoV-2 detected by RT-PCR on self-collected SNIFF-provided nasal swabs, right-censored at the last negative RT-PCR test at or before the earliest of: a COVID-defining endpoint from the parent trial, termination or receipt of EUA vaccine.
Data set: Post crossover analyses set (PCAS)
Approach: The analysis in section 10.2 will be repeated for this outcome.

11. EXPLORATORY ENDPOINTS

This section describes the planned analyses for exploratory endpoints. As appropriate for exploratory endpoints, we expect that these analyses will be supported by ad hoc analyses as suggested by the data.

11.1 Exploratory Objective: To determine the sequence of breakthrough SARS-CoV-2 infections

SARS-CoV-2 Genome Sequence Analysis – Samples with “detected” interpretations by the N1/N3 SARS-CoV-2 assay will be referred for additional screening and whole genome sequencing. Samples with N1 cycle threshold values (Ct) of <= 32 will be prepared for whole genome sequencing. Samples with Ct

values > 32 will be evaluated according to their likelihood to generate a quality genome sequence to maximize the number of viral genomes from unique research participants. The genomic data will be analyzed to produce a pangolin lineage call (ex. B.1.1.7) and Greek alpha label (ex. Alpha) as applicable for variants of interest/concern.

11.2 Exploratory Objective: To estimate the VE on duration of infection

Endpoint: Duration of shedding as indicated by RT-PCR as measured by the number of RT-PCR positive swabs. If a swab is missing between two RT-PCR positive swabs, it will be treated as if it were RT-PCR positive. If there are missing swabs just before the first or after the last RT-PCR positive swab, the endpoint will be interval censored with the interval being: (number of RT-PCR positive swabs, number of RT-PCR positive swabs + number of missing adjacent swabs).
Data sets: PP-ITT, PP-PAS, PP-PAS2
Approach: A Poisson model will be fit with randomization arm as the predictor of interest. Covariates will be included to account for potential post-randomization selection bias. These will include sex, age, and presence of comorbidities measured at the time point closest to but before infection.

11.3 Exploratory Objective: To estimate the VE against SARS-CoV-2 viral load as a proxy of transmission

Endpoint: Peak viral load (and subgenomic RNA, if tested), as a proxy for transmission, in RT-PCR positive swabs
Data sets: PP-ITT, PP-PAS, PP-PAS2
Approach: Linear regression summaries of the peak viral load on the log10 scale will be used with vaccination status (no vaccine, 1 dose, 2 doses) as the covariate of interest, adjusted for age and sex.

11.4 Exploratory Objective: To estimate the efficacy of SARS-CoV-2 rS/M1 vaccine against a composite outcome of asymptomatic/very mildly symptomatic infection plus pre-symptomatic infection

Endpoint: Proportion of days that are symptom-free on days that RT-PCR positive swabs are collected
Data sets: mITT, PAS, PAS2
Approach: Poisson model with log offset of number of days with RT-PCR positive swabs and appropriate adjustment for post randomization selection bias as determined in supporting analyses for the primary endpoint.

11.5 Exploratory Objective: To explore whether observed VE against COVID-19 illness reflects a transition from symptomatic into asymptomatic infections versus an absolute reduction in all SARS-CoV-2 infections

Endpoint: Ratio of asymptomatic to symptomatic infections in vaccinated vs unvaccinated (defined as pre-second dose) recipients. Data on symptomatology will be extracted from the Phase 3 trial database, which will be separate but linkable to the ancillary study.
Approach: This objective will be met as a composite of previous analyses and will be addressed in a synthesizing discussion of overall results. Post hoc analyses may be added to aid in understanding of results.

12. ADDITIONAL PLANNED ANALYSES

In this section, we list planned supporting analyses at a high level. Details for these analyses will be guided from the results reported based on the analyses in this document.

- Repeat analyses in calendar time instead of study time.

All proprietary rights reserved

- Infection endpoint expanded to include serological testing from the parent study.
- Estimating the association between time since vaccination and characteristics of viral kinetics.
- Modeling of viral load curves to estimate duration of shedding in days as well as peak viral loads. Models are in development that extend those presented in Stankiewicz Karta et al (2021).
- Multistate models to estimate the disease course, reflecting transitions from uninfected to asymptomatic infection (definition may vary), to symptomatic infection to recovery. This would be similar to an illness-death model.
- Models that treat vaccination status as a time-varying covariate. These models will also need to include information on background distribution of variants over time and geography.
- Models that compare total potential infectious time averted due to vaccination.

13. REFERENCES

Stankiewicz Karita, Helen C., et al. "Trajectory of viral load in a prospective population-based cohort with incident SARS-CoV-2 G614 infection." *medRxiv* (2021).

14. CHANGE HISTORY

Version		Affected Section(s)	Activity Description
Number	Effective Date		

All proprietary rights reserved

Final Statistical Analysis Plan Version 2.0

STATISTICAL ANALYSIS PLAN

A nasal swab study to assess the efficacy of vaccination in the prevention of SARS-CoV-2 infection among individuals enrolled in a Phase 3 efficacy trial of a SARS-CoV-2 recombinant spike protein (rS) vaccine with Matrix-M1™ (M1) adjuvant

DMID Protocol Number: 21-0011
CoVPN Number: 3004-01

Effective Date: 30/06/2022

Version: 2.0

All proprietary rights reserved

STATISTICAL ANALYSIS PLAN

**Protocol Name:**	A nasal swab study to assess the efficacy of vaccination in the prevention of SARS-CoV-2 infection among individuals enrolled in a Phase 3 efficacy trial of a SARS-CoV-2 recombinant spike protein (rS) vaccine with Matrix-M1™ (M1) adjuvant
**Protocol Number:**	**DMID Protocol Number:** 21-0011
**Author(s):**	Elizabeth R. Brown, ScD<br>Yuqing Jiao, MS
**Version:**	*2.0*

**Author(s):**

Elizabeth Brown, ScD	*See appended approval*	*See appended approval*
Lead Statistician	Signature	Date: 30/06/2022
Yuqing Jiao	*See appended approval*	*See appended approval*
Statistical Research Associate	Signature	Date: 30/06/2022

TABLE OF CONTENTS

1. LIST OF ABBREVIATIONS, ACRONYMS AND DEFINITIONS 5

2. INTRODUCTION 5

2.1 General Design Considerations 5
2.2 Study Objectives and Endpoints 6
2.3 Randomization 7
2.4 Blinding 8
2.5 Sample Size and Power 8

3. GENERAL DATA ANALYSIS CONSIDERATIONS 9

3.1 Analysis Set(s) 9
3.2 Statistical Analysis Issues 9
3.3 Incorrect/missing Swabbing Date 10

4. INTERIM ANALYSIS AND DATA MONITORING COMMITTEE 11

5. GENERAL ANALYSIS METHODS 11

6. TRIAL PARTICIPANT DISPOSITION 11

6.1 Disposition of Participant 11
***6.1.1 Screening and Enrollment** 11*
***6.1.2 Compliance to Swabbing** 11*
***6.1.3 Study Discontinuation** 12*
6.2 Treatment Exposure 12

7. BASELINE DATA 12

8. SAFETY ANALYSES 12

9. PRIMARY EFFICACY/EFFECTIVENESS ANALYSES 13

9.1 Descriptive measures of SARS-CoV-2 acquisition: 13
9.2 Primary Objective: Prevention of Infection 13

10. SECONDARY ENDPOINTS 15

10.1 Secondary Objective: Prevention of Asymptomatic/Very Mild Symptomatic Infection (A/VMS) 15
10.2 Secondary Objective: To determine the durability of efficacy of SARS-CoV-2 rS/M1 vaccine against infection 15
10.3 Secondary Objective: To determine the durability of efficacy of SARS-CoV-2 rS/M1 vaccine against asymptomatic/very mildly symptomatic infection 15

11. EXPLORATORY ENDPOINTS 15

11.1 Exploratory Objective: To determine the sequence of breakthrough SARS-CoV-2 infections 15
11.2 Exploratory Objective: To estimate the VE on duration of infection 16

All proprietary rights reserved

11.3 Exploratory Objective: To estimate the VE against SARS-CoV-2 viral load as a proxy of transmission ... 16
11.4 Exploratory Objective: To estimate the efficacy of SARS-CoV-2 rS/M1 vaccine against a composite outcome of asymptomatic/very mildly symptomatic infection plus pre-symptomatic infection ... 16
11.5 Exploratory Objective: To explore whether observed VE against COVID-19 illness reflects a transition from symptomatic into asymptomatic infections versus an absolute reduction in all SARS-CoV-2 infections ... 16

12. ADDITIONAL PLANNED ANALYSES ... 16

13. REFERENCES ... 17

14. CHANGE HISTORY ... 17

All proprietary rights reserved

1. LIST OF ABBREVIATIONS, ACRONYMS AND DEFINITIONS

Term/Abbreviation	Definition
IDCRC	Infectious Disease Clinical Research Consortium
SDSU	Statistical and Data Sciences Unit
parent study	A Phase 3, Randomized, Observer-Blinded, Placebo-Controlled Study to Evaluate the Efficacy, Safety, and Immunogenicity of a SARS-CoV-2 Recombinant Spike Protein Nanoparticle Vaccine (SARS-CoV-2 rS) with Matrix-M1™ Adjuvant in Adult Participants ⩾ 18 Years with a Pediatric Expansion in Adolescents (12 to 17 Years). Protocol 2019nCoV-301
Initial series	The series of two injections of either vaccine or placebo given on days 0 and 21
Crossover series	The second series of two injections of either vaccine or placebo given at crossover and 21 days later

2. INTRODUCTION

This SAP covers the final analysis of data collected in the SNIFF Study, a substudy of adolescents aged 12-17 enrolled in the Novavax study (the parent study).

2.1 General Design Considerations

**Short Title:**	SNIFF
**Design of the Study:**	In this ancillary swab study, up to 3,000 adolescent participants 12-17 years of age in the Phase 3 SARS-CoV-2 vaccine trial (PREVENT-19) sponsored by Novavax will be sampled twice weekly for approximately 16-28 weeks with nasal swabs for detection of SARS-CoV-2 infection by RT-PCR and incident infections tallied.
**Study Phase:**	Observational
**Study Population:**	Subset of participants in Protocol 2019nCoV-301, entitled "A Phase 3, Randomized, Observer-Blinded, Placebo-Controlled Study to Evaluate the Efficacy, Safety, and Immunogenicity of a SARS-CoV-2 Recombinant Spike Protein Nanoparticle Vaccine (SARS-CoV-2 rS) with Matrix-M1™ Adjuvant in Adult Participants ≥ 18 Years with a Pediatric Expansion to adolescents (12 to 17 years)" (PREVENT-19)
**Study Duration**	Follow-up is expected to be completed December 2021. Total duration of the study will be approximately 6 months from first enrolment.
**Number of Sites:**	Up to 75 sites located in the US

All proprietary rights reserved

Description of Study Product or Intervention:

Participants in the PREVENT-19 Phase 3 trial (pediatric expansion study) are randomized to receive either SARS-CoV-2 rS/M1 vaccine or placebo (2:1 allocation) on days 0 and 21. The pediatric expansion study will have a "blinded-crossover" after ~3 months of follow-up after the completion of the initial set of vaccinations. Pediatric participants will be scheduled for administration of 2 injections of the alternate study material 21 days apart. In the current protocol, no interventional product will be administered.

2.2 Study Objectives and Endpoints

Objectives	Endpoints
Primary	
• To estimate the efficacy of SARS-CoV-2 rS/M1 vaccine against infection	• Reverse transcriptase polymerase chain reaction (RT-PCR)-confirmed SARS-CoV-2 infection detected in nasal swabs collected twice weekly from the day 21 visit or thereafter, until the second dose in the second vaccination (crossover) series in the vaccine group and the placebo group.
Secondary	
• To estimate the efficacy of SARS-CoV-2 rS/M1 vaccine against asymptomatic/ very mildly symptomatic infection	• RT-PCR-confirmed SARS-CoV-2 infection detected in nasal swabs collected twice weekly from the day 21 visit or thereafter, until the second dose in the second vaccination (crossover) series, without COVID-19 illness as defined in the primary Phase 3 study endpoint. The COVID-19 disease endpoint will be defined in the main study database.
• To determine the durability of efficacy of SARS-CoV-2 rS/M1 vaccine against infection	• RT-PCR-confirmed SARS-CoV-2 infection detected in nasal swabs collected twice weekly from the crossover dose 2 visit (or thereafter) until ancillary study end among the group who initially received vaccine compared to the group who initially received placebo

• To determine the durability of efficacy of SARS-CoV-2 rS/M1 vaccine against asymptomatic/very mildly symptomatic infection	• RT-PCR-confirmed SARS-CoV-2 infection detected in nasal swabs collected twice weekly from the crossover dose 2 visit (or thereafter) until the second dose in the second vaccination (crossover) series among the group who initially received vaccine compared to the group who initially received placebo without COVID-19 illness as defined in the primary Phase 3 study endpoint. The COVID-19 disease endpoint will be defined in the main study database.
Exploratory	
• To determine the sequence of breakthrough SARS-CoV-2 infections*	• Viral genome sequence of RT-PCR-positive samples
• To estimate the VE on duration of infection	• Duration of shedding as indicated by RT-PCR in vaccinated vs placebo recipients
• To estimate the VE against SARS-CoV-2 viral load as a proxy of transmission	• Reduction in viral load (and perhaps subgenomic RNA), as a proxy for transmission, in RT-PCR-positive samples from vaccinated vs placebo recipients
• To estimate the efficacy of SARS-CoV-2 rS/M1 vaccine against a composite outcome of asymptomatic/very mildly symptomatic infection plus pre-symptomatic infection	• Proportion of days that are symptom-free on days that RT-PCR-positive swabs are collected
• To explore whether observed VE against COVID-19 illness reflects a transition from symptomatic into asymptomatic infections versus an absolute reduction in all SARS-CoV-2 infections	• Ratio of asymptomatic to symptomatic infections in vaccinated vs placebo recipients. Data on symptomatology will be extracted from the Phase 3 trial database, which will be separate but linkable to the ancillary

*This document will provide analytical plans for all but the first exploratory endpoint.

2.3 Randomization

Randomization is part of the parent study. All details are included in that protocol.

2.4 Blinding

The blinding codes are not maintained or accessible by SCHARP staff or any members of the IDCRC SDSU and will only be provided to SCHARP after the lock of the database for this study.

2.5 Sample Size and Power

Very little data are available on incidence rates of SARS-CoV-2 infection in children aged 12-17. The CDC (https://www.cdc.gov/coronavirus/2019-ncov/cases-updates/burden.html) estimates a cumulative incidence in children 5-17 years old of 27,218 per 100,000 from February 2020-December 2021 which translates into approximately 5.7 per 1,000 person-weeks. A similar incidence estimate can be calculated based on prevalence in children presenting for non-COVID-19 related hospital visits in the summer of 2020 (Sola et al, JAMA Pediatr 2021). When estimating power for the protocol, we estimated that 2,000 children would enroll and complete follow-up with twice per week swabbing. Figure 1 provides the power (with two-sided alpha = 0.05) to reject the null hypothesis that VE=0 under various scenarios: VEs of 0.4, 0.6 and 0.8 and attack rates equal to or 0.5 to 2 times that observed in 2020. For example, if the attack is equal to 5.7 per 1,000 person-weeks with 6 weeks of follow-up, there is 80% power to detect a VE of 60%. If the attack rate is half that from 2020 (2.9 per 1,000 person-weeks) with 6 weeks of follow-up, then the study has >80% power to detect VE of 80%. These calculations assume all infections will be detected with twice per week swabbing.

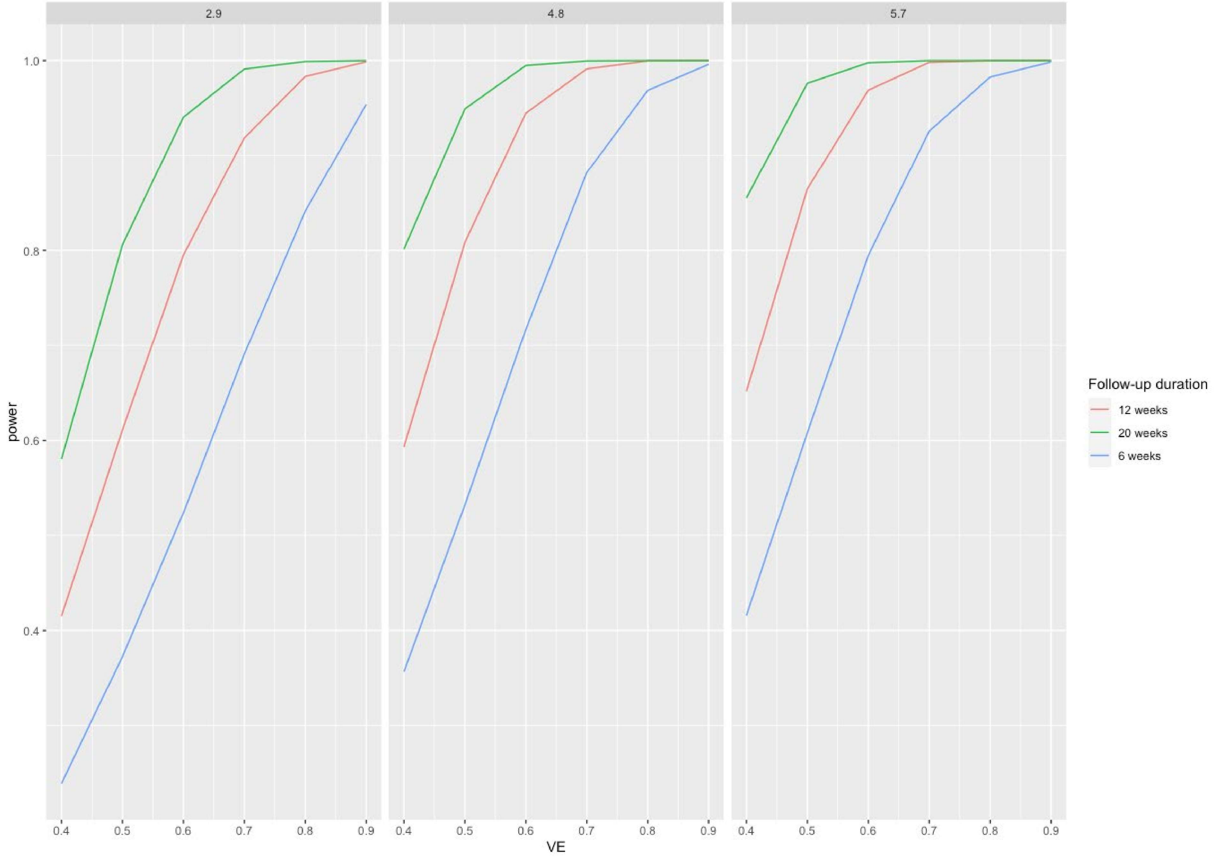

Figure 1 Power to detect vaccine efficacy against infection with 2000 participants, assuming follow-up for 6, 12 or 20 weeks and incidence rate equal to 2.9 per 1,000 person-weeks (left panel), 4.8 per 1,000 person-weeks (center panel), and 5.7 per 1,000 person-weeks.

All proprietary rights reserved

3. GENERAL DATA ANALYSIS CONSIDERATIONS

3.1 Analysis Set(s)

For analysis purposes, the following populations are defined:

Intention to Treat (ITT): All enrolled participants who had at least one valid result in the lab results dataset

Modified Intention to Treat (mITT): All ITT participants who were not identified as having SARS-CoV-2 infection, either through RT-PCR or serological testing, prior to administration of the second dose of the first dosing series.

Primary analysis set (PAS): The intention of the SNIFF protocol was to enroll participants at their second dose of the first vaccination series and immediately start swabbing. This would allow exclusion of all participants who were previously infected. However, many participants enrolled after the second dose of the initial series, and it may be impossible to determine if they acquired infection in between that second dose and the first swab. We define this data set to account for this by limiting inclusion to mITT participants enrolled before 4 weeks post second dose of the initial series. Primary analyses will be conducted on this data set.

Primary analyses set 2 (PAS2): All mITT participants enrolled by July 12, 2021. Primary analyses will be conducted on this data set. This analysis set serves as a complement to PAS in case too many participants have enrolled after 4 weeks post second dose of the primary series. 82.4% were enrolled by this date.

Post crossover analysis set (PCAS): All mITT participants enrolled before 4 weeks post second dose of the crossover series without evidence of prior infection before or at the second dose of the crossover series.

PCR-positive (PP-ITT): All participants in the ITT population with SARS-CoV-2 RNA detected by RNA-RT-PCR as part of the SNIFF study (as determined by the lab).

PCR-positive PAS (PP-PAS): All participants in the PAS with SARS-CoV-2 RNA detected by RNA-RT-PCR as part of the SNIFF study (as determined by the lab).

PCR-positive PAS (PP-PAS2): All participants in the PAS2 with SARS-CoV-2 RNA detected by RNA-RT-PCR as part of the SNIFF study (as determined by the lab).

3.2 Statistical Analysis Issues

Key information such as product assignment (randomization), clinical symptomatology, adjudicated COVID-19 illness endpoints, RNA PCR results, demographics, risk factors, timing of vaccination, outside vaccination receipt date, and laboratory results (specifically seroconversion indicative of prior infection) are collected in the parent protocol. Those data will be sent to SCHARP after the study ends to merge with the SNIFF data for analyses.

All proprietary rights reserved

This document was written without knowledge of the primary study database or access to any data summaries from the parent study, including enrollment characteristics of the population, and may need to be updated once this database is provided to the statistical team.

SARS-CoV-2 Viral Load

Viral load assessment will be conducted via digital PCR methods using N1 and N3 target sets and expressed in copies/swab on a per sample basis for all specimens with a "detected" interpretation.

Change of predominant variant in the United States during follow-up time

From the first enrollment in this study to the end of follow-up, the predominant SARS-Cov-2 strain changed from alpha to delta (Figure 2). We will plan to repeat analyses in the subset of sites in states where delta was the most prevalent strain (>80%) at the start of enrollment (according to nextstrain.org).

Durability analysis

This SAP reflects a version of the protocol in which durability was included as a secondary endpoint. Due to the changing prevalence of variants throughout the follow-up time and the expedited crossover timelines in the parent study, it will be challenging to estimate durability. First, the follow-up time for the group vaccinated early may be too short to expect a meaningful decline in efficacy. Second, durability estimates will be confounded by variant (see Figure 2).

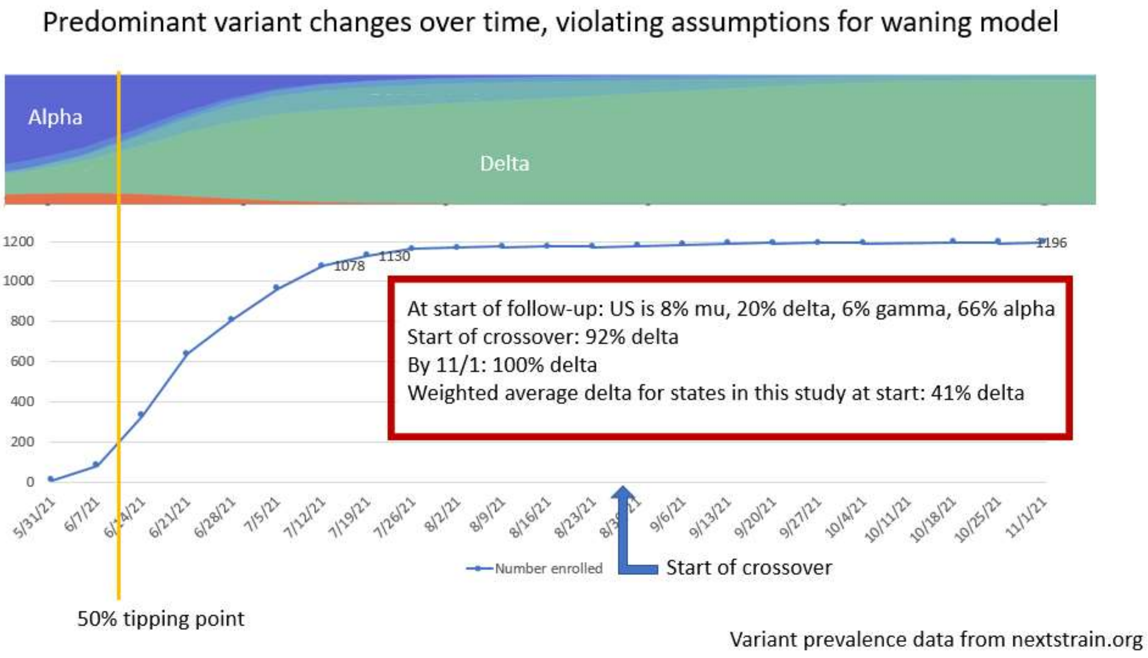

Figure 2 Enrollment into SNIFF and prevalence of predominant variants in the United States over calendar time. Data on prevalence of variants is provided by nextstrain.org, accessed on 11/30/2021.

3.3 Incorrect/missing Swabbing Date

At times in the study, participants neglected to scan their swabs before returning them or scanned multiple swabs on the same date. Cases with evidence of an incident infection, either by receipt of a

swab with RNA detected by PCR, or evidence of infection from the parent study, will be reviewed by an adjudication committee to determine timing of infection.

4. INTERIM ANALYSIS AND DATA MONITORING COMMITTEE

No interim analyses were planned for this study nor was there a data monitoring committee.

5. GENERAL ANALYSIS METHODS

Descriptive statistics that will be used to summarize continuous variables are as follows: mean and standard deviation, median and interquartile range, quartiles, range, and number of missing data values. For categorical variables, descriptive statistics that will be used include the following: frequencies, relative frequencies, and the number of missing data values. Descriptive analyses summarizing baseline and follow-up data will be stratified by state. Line, scatter, and box plots will be used, as appropriate, for longitudinal data representations. Covariates from regression models will be assessed using Wald tests and corresponding 95% confidence intervals, unless otherwise specified. A two-sided alpha level of 0.05 will be used for all statistical tests, unless otherwise specified.

6. TRIAL PARTICIPANT DISPOSITION

6.1 Disposition of Participant

6.1.1 Screening and Enrollment

Dates of site activation, first enrollment, and last enrollment, as well as the number of participants screened, number and percentage of participants enrolled, and the screening-to-enrollment ratio will be displayed in a table overall and by site and randomization arm.

The number of participants included in each data set will be tabulated by site and randomization arm.

Reasons for participants who are not eligible for the study will be displayed in a listing.

6.1.2 Compliance to Swabbing

Participants are expected to swab on Mondays and Thursdays. Thus the weeks are separated into two collection windows – window1 (Monday, Tuesday, Wednesday) and window 2 (Thursday, Friday, Saturday, Sunday). Alluvial plots will be used to capture completeness of swabbing. Categories: 0 windows, 1 window, or 2 windows per week, discontinued (exited study). These will be created to describe the population overall and by randomization arm.

The number of expected swabs versus resulted scanned swabs will be tabulated overall and by site and randomization arm.

All proprietary rights reserved

6.1.3 Study Discontinuation

The number and percentages of participants who completed the study, as well as the reasons for non-completion, will be displayed in a table overall and by site and by randomization arm and by follow-up period.
Time from enrollment to study discontinuation and time from crossover to study discontinuation will be summarized using Kaplan-Meier curves overall and by arm.

6.2 Treatment Exposure

Treatment exposure will be defined by randomization arm from the parent protocol and number of doses received. The number and percentage of participants who received no doses, one dose, and two doses will be displayed in a table overall and by site and randomization arm.

7. BASELINE DATA

Baseline demographic characteristics of the participants, such as site, state, age, sex, randomization arm, race, exposure risk information such as whether “the subject is a student attending school in person” will be displayed in a table overall and by site, and in a table overall and by randomization arm. Baseline is defined as time of enrollment into SNIFF. Demographic characteristics are collected in the parent study with a collection time that may not align with enrollment into SNIFF; therefore, baseline characteristics will be determined from the data collected most recently before enrollment into SNIFF.

The set of characteristics collected at baseline (including randomization arm) in the parent study will be compared between those who enrolled in SNIFF versus those who did not.

8. SAFETY ANALYSES

Limited safety data were collected as part of the SNIFF study and are expected to be low in frequency. If applicable, summaries of adverse events will include a cumulative listing of adverse events and a table displaying the total number of adverse events by severity grade and relationship to study product.

All proprietary rights reserved

9. PRIMARY EFFICACY/EFFECTIVENESS ANALYSES

9.1 Descriptive measures of SARS-CoV-2 acquisition:

Table 1 Table for use when summarizing incident outcomes, including infection, overall and with and without COVID-defining symptoms.

Randomization arm	Exposure	Number of participants	Number of incident endpoints	Person-years	Incidence rate (95% CI)
Vaccine -> Placebo	Two doses of vaccine			See note 2	
Placebo -> Vaccine	Single dose of vaccine			See note 1	
	Two doses of vaccine			See note 2	
	Unvaccinated (two placebo doses)			See note 3	

Note 1: Total number of person-years post first dose and pre first of second dose in the crossover period, infection (maybe due to RT-PCR or antibody evidence of infection as per endpoints in analyses described below) or censoring.
Note 2: Total number of person-years post second dose and pre first of infection (maybe due to RT-PCR or antibody evidence of infection as per endpoints in analyses described below) or censoring.
Note 3: Total number of person-years post second dose of placebo and pre first of infection (maybe due to RT-PCR or antibody evidence of infection as per endpoints in analyses described below) or censoring.

The table above will be replicated by time periods, stratifying by calendar month as well as pre and post crossover. Results will be plotted over time. Additional calendar periods may be defined by predominant variant at the site with further stratification by the variant associated with the endpoint, recognizing that not all infections have enough virus present to be sequenced.

Descriptive Plots
Kaplan-Meier curves will be used to display incidence of infection across study groups. Multiple curves will be produced, corresponding to the four exposure groups in Table 1 for the PAS, PAS2 and PCAS. Endpoint definitions follow in the analysis descriptions for primary and secondary analyses.

Each of these sets of plots will be produced once where the time scale is calendar time and once where the time scale is study time (time since enrollment).

9.2 Primary Objective: Prevention of Infection

Primary analysis
Endpoint: SARS-CoV-2 detected by RT-PCR on self-collected SNIFF-provided nasal swabs, right-censored at the last negative test at or before the earliest of the second dose of the crossover vaccination series (vaccine or placebo), 4 weeks post first crossover dose (applicable for cases where the second dose in the crossover period is missed or late), termination, or receipt of EUA vaccine.
Data sets: Primary analyses set; supported by analyses on the mITT set

All proprietary rights reserved

Approach: Incidence of SARS-CoV-2 infection will be analyzed using a Cox Proportional Hazards model using follow-up time through 4 weeks post crossover. Calendar time will not be accounted for in the primary analysis since enrollment was completed in a short window of time. This analysis only counts infections until the second dose of the crossover series. Follow-up time may be right-censored earlier if a participant receives an EUA vaccine or otherwise is terminated early from the study. Randomization arm will be merged from the primary study database. Efficacy will be estimated as 1-HR (hazard ratio) from a Cox proportional hazards model. The primary analysis will not use any endpoint information collected in the parent study. This analysis will only include randomization as a covariate. The proportional hazards assumption will be assessed graphically.

Sensitivity analysis 1

The analysis will be repeated counting follow-up time up to 2 weeks post first dose of the crossover series.

Endpoint: SARS-CoV-2 detected by RT-PCR on self-collected SNIFF-provided nasal swabs, right-censored at the last negative test at or before the earliest of the second dose of the crossover series (vaccine or placebo), 2 weeks post first crossover dose (applicable for cases where the second dose is missed or late), termination, or receipt of EUA vaccine.

Supporting analysis 1

If any participants are identified as having infection in the parent study but not in the SNIFF study, the primary analysis will be repeated by revising the primary endpoint to include the first time to SARS-CoV-2 infection as defined in the parent study or SNIFF.

Endpoint: SARS-CoV-2 detected by RT-PCR on self-collected SNIFF-provided or Novavax-provided nasal swabs, right-censored at the collection time of the last RT-PCR negative swab at or before the earliest of the second dose of the crossover series (vaccine or placebo), 2 weeks post first crossover dose (applicable for cases where the second dose is missed or late), termination, or receipt of EUA vaccine. Note: Novavax-provided swabs may either be self-collected at home or collected at study visits.

Supporting analysis 2

Because the SNIFF population is a subset of the parent study and was selected post-randomization from the parent study, the primary analysis and supporting analysis 1 will be repeated with adjustment for potential confounders. These will be selected from baseline variables that are univariably (adjusted for state) predictive of risk of acquisition of infection or differ by randomization arm with $p<0.10$. If there are inadequate numbers of participants per state, state may be dropped as an adjustment variable.

Supporting analysis 3: Delta variant analysis

Supporting analysis 2 will be repeated with each person's follow-up time starting at the time at which delta variant became over 80% predominant in their state according to nextstrain.org. Adjustment for covariates will be included as per supporting analysis 2.

All proprietary rights reserved

10. SECONDARY ENDPOINTS

10.1 Secondary Objective: Prevention of Asymptomatic/Very Mild Symptomatic Infection (A/VMS)

Endpoint: SARS-CoV-2 detected by RT-PCR on self-collected SNIFF-provided nasal swabs, right-censored at the last negative test at or before the earliest of: a COVID-defining endpoint from the parent trial, 4 weeks post first crossover dose, termination, or receipt of EUA vaccine.
Data sets: Primary analyses set; supported by analyses on the mITT set
Approach: We will use the approach presented for the primary outcome to ensure proper accounting for variable follow-up time due to early crossover. Data sources from the Phase 3 trial (e.g., study product assignment and clinical symptomatology, including adjudicated COVID-19 illness endpoints) will be accessed to ascertain the symptomatology of participants who are RT-PCR-positive in the ancillary study.

Table 1 in Section 9.1 will also be completed for this endpoint.

10.2 Secondary Objective: To determine the durability of efficacy of SARS-CoV-2 rS/M1 vaccine against infection

Endpoint: SARS-CoV-2 detected by RT-PCR on self-collected SNIFF-provided nasal swabs, right-censored at the last negative RT-PCR test at or before the earliest of termination or receipt of EUA vaccine.
Data set: Post crossover analyses set (PCAS)
Approach: The analyses for the primary endpoint will be repeated with the time origin being time of second dose (placebo or active) in the crossover series. Durability will be assessed through a time-invariant hazard ratio with supporting analyses investigating evidence of non-proportionality of the hazards that could indicate loss of efficacy in the early vaccination arm. The study may not be adequately powered for this analysis. Evidence will also be supported by the data that will be presented in the mock table (Table 1 in this document) which will examine incidence rates over calendar time in both randomization arms.

If there is adequate power, the supporting analysis for the primary objective will be repeated for this objective.

10.3 Secondary Objective: To determine the durability of efficacy of SARS-CoV-2 rS/M1 vaccine against asymptomatic/very mildly symptomatic infection

Endpoint: SARS-CoV-2 detected by RT-PCR on self-collected SNIFF-provided nasal swabs, right-censored at the last negative RT-PCR test at or before the earliest of: a COVID-defining endpoint from the parent trial, termination or receipt of EUA vaccine.
Data set: Post crossover analyses set (PCAS)
Approach: The analysis in section 10.2 will be repeated for this outcome.

11. EXPLORATORY ENDPOINTS

This section describes the planned analyses for exploratory endpoints. As appropriate for exploratory endpoints, we expect that these analyses will be supported by ad hoc analyses as suggested by the data.

11.1 Exploratory Objective: To determine the sequence of breakthrough SARS-CoV-2 infections

SARS-CoV-2 Genome Sequence Analysis – Samples with “detected” interpretations by the N1/N3 SARS-CoV-2 assay will be referred for additional screening and whole genome sequencing. Samples with N1 cycle threshold values (Ct) of <= 32 will be prepared for whole genome sequencing. Samples with Ct

values > 32 will be evaluated according to their likelihood to generate a quality genome sequence to maximize the number of viral genomes from unique research participants. The genomic data will be analyzed to produce a pangolin lineage call (ex. B.1.1.7) and Greek alpha label (ex. Alpha) as applicable for variants of interest/concern.

11.2 Exploratory Objective: To estimate the VE on duration of infection

Endpoint: Duration of shedding as indicated by RT-PCR as measured by the number of RT-PCR positive swabs. If a swab is missing between two RT-PCR positive swabs, it will be treated as if it were RT-PCR positive. If there are missing swabs just before the first or after the last RT-PCR positive swab, the endpoint will be interval censored with the interval being: (number of RT-PCR positive swabs, number of RT-PCR positive swabs + number of missing adjacent swabs).
Data sets: PP-ITT, PP-PAS, PP-PAS2
Approach: A Poisson model will be fit with randomization arm as the predictor of interest. Covariates will be included to account for potential post-randomization selection bias. These will include sex, age, and presence of comorbidities measured at the time point closest to but before infection.

11.3 Exploratory Objective: To estimate the VE against SARS-CoV-2 viral load as a proxy of transmission

Endpoint: Peak viral load (and subgenomic RNA, if tested), as a proxy for transmission, in RT-PCR positive swabs
Data sets: PP-ITT, PP-PAS, PP-PAS2
Approach: Linear regression summaries of the peak viral load on the log10 scale will be used with vaccination status (no vaccine, 1 dose, 2 doses) as the covariate of interest, adjusted for age and sex.

11.4 Exploratory Objective: To estimate the efficacy of SARS-CoV-2 rS/M1 vaccine against a composite outcome of asymptomatic/very mildly symptomatic infection plus pre-symptomatic infection

Endpoint: Proportion of days that are symptom-free on days that RT-PCR positive swabs are collected
Data sets: mITT, PAS, PAS2
Approach: Poisson model with log offset of number of days with RT-PCR positive swabs and appropriate adjustment for post randomization selection bias as determined in supporting analyses for the primary endpoint.

11.5 Exploratory Objective: To explore whether observed VE against COVID-19 illness reflects a transition from symptomatic into asymptomatic infections versus an absolute reduction in all SARS-CoV-2 infections

Endpoint: Ratio of asymptomatic to symptomatic infections in vaccinated vs unvaccinated (defined as pre-second dose) recipients. Data on symptomatology will be extracted from the Phase 3 trial database, which will be separate but linkable to the ancillary study.
Approach: This objective will be met as a composite of previous analyses and will be addressed in a synthesizing discussion of overall results. Post hoc analyses may be added to aid in understanding of results.

12. ADDITIONAL PLANNED ANALYSES

In this section, we list planned supporting analyses at a high level. Details for these analyses will be guided from the results reported based on the analyses in this document.

- Repeat analyses in calendar time instead of study time.

- Infection endpoint expanded to include serological testing from the parent study.
- Estimating the association between time since vaccination and characteristics of viral kinetics.
- Modeling of viral load curves to estimate duration of shedding in days as well as peak viral loads. Models are in development that extend those presented in Stankiewicz Karta et al (2021).
- Multistate models to estimate the disease course, reflecting transitions from uninfected to asymptomatic infection (definition may vary), to symptomatic infection to recovery. This would be similar to an illness-death model.
- Models that treat vaccination status as a time-varying covariate. These models will also need to include information on background distribution of variants over time and geography.
- Models that compare total potential infectious time averted due to vaccination.

13. REFERENCES

Stankiewicz Karita, Helen C., et al. "Trajectory of viral load in a prospective population-based cohort with incident SARS-CoV-2 G614 infection." *medRxiv* (2021).

14. CHANGE HISTORY

Version		Affected Section(s)	Activity Description
Number	Effective Date		
2.0	June 30, 2022	Section 3.1	Section 3.1: Update ITT and mITT criteria

All proprietary rights reserved

From: Brown ScD, Elizabeth R
To: Jiao, Yuqing
Subject: Re: Please Review and Approve SNIFF SAP v2.0
Date: Thursday, June 30, 2022 2:23:54 PM

I, Elizabeth Brown, ScD, Professor, approve. This email is substitute for a handwritten signature on SNIFF SAP v2.0.

From: Jiao, Yuqing <yjiao@scharp.org>
Date: Thursday, June 30, 2022 at 2:22 PM
To: Brown ScD, Elizabeth R <erbrown@fredhutch.org>
Subject: Please Review and Approve SNIFF SAP v2.0

Hi Elizabeth,

Could you review and approved the attached SNIFF SAP v2.0 please? If you approve, please reply with this statement indicating approval:
"I, (legal first and last name, job title), approve. This email is substitute for a handwritten signature on SNIFF SAP v2.0."

Thank you,
Yuqing

From: Jiao, Yuqing
To: Jiao, Yuqing
Subject: RE: Please Review and Approve SNIFF SAP v2.0
Date: Thursday, June 30, 2022 2:23:12 PM

I, Yuqing Jiao, statistical research associate, approve. This email is substitute for a handwritten signature on SNIFF SAP v2.0.

From: Jiao, Yuqing <yjiao@scharp.org>
Sent: Thursday, June 30, 2022 2:22 PM
To: Jiao, Yuqing <yjiao@scharp.org>
Subject: Please Review and Approve SNIFF SAP v2.0

Hi,

Could you review and approved the attached SNIFF SAP v2.0 please? If you approve, please reply with this statement indicating approval:
"I, (legal first and last name, job title), approve. This email is substitute for a handwritten signature on SNIFF SAP v2.0."

Thank you,
Yuqing

Statistical Analysis Plan Changes from Original (Version 1.0)

to Final (Version 2.0)

Protocol 21-0011 Summary of change in Statistical Analysis Plan
Version 1.0 (21 April 2022) to version 2.0 – Final (30 June 2022)

Version		Affected Section(s)	Activity Description
Number	Effective Date		
2.0	June 30, 2022	Section 3.1	Section 3.1: Update ITT and mITT criteria

RESEARCH ASSENT
V2.0; 22 APR 2021

Protocol Title: A nasal swab study to assess the efficacy of vaccination in the prevention of SARS-CoV-2 infection among individuals enrolled in a Phase 3 efficacy trial of a SARS-CoV-2 recombinant spike protein (rS) vaccine with Matrix-M1™ (M1) adjuvant

Principal Investigator: *[Investigator name]*
During office hours: [investigator contact number]
After hours and holidays: [investigator contact number]

We want to tell you about a research study we are doing. A research study is a way to learn information about something. We would like to find out more about whether SARS-CoV-2 rS/M1 vaccine prevents SARS-CoV-2 infection, including infections that cause no symptoms or very mild symptoms. SARS-CoV-2 is the virus that causes COVID-19.

We know that many people become infected with SARS-CoV-2 without ever becoming ill and can pass the infection to others. The purpose of the swab study is to find out whether the vaccine also prevents people from becoming infected with SARS-CoV-2. If you agree to join the swab study, you (or your parent) will collect swabs from your nose at home twice a week for about 20 weeks by gently placing a nasal swab (a small stick with a soft tip) into the nose and twirling it.

We plan to enroll up to 3,000 children in this study. If you agree to join this study, you will be asked to allow us to collect information about you.

You will not be helped by being in this study. We may learn something that will help other people in the future. Being in the study (or not) won't change how your doctors and nurses care for you.

You do not have to join this study. It is up to you. You can say okay now, and you can change your mind later. All you have to do is tell us. No one will be mad at you if you change your mind.

Before you say yes to be in this study, we will answer any questions you have.

If you want to be in this study, please sign your name. You will get a copy of this form to keep for yourself.

Study Participant

____________________________________ ___________________
(Sign your name here) (Date)

____
(Print your name here)

Person Obtaining Assent

_____________________________________ ___________________
(Sign your name here) (Date)

____
(Print your name here)

RESEARCH CONSENT FORM AND HIPAA AUTHORIZATION
(Parent or Guardian Permission Form)
V2.0; 22 APR 2021

Protocol Title: A nasal swab study to assess the efficacy of vaccination in the prevention of SARS-CoV-2 infection among individuals enrolled in a Phase 3 efficacy trial of a SARS-CoV-2 recombinant spike protein (rS) vaccine with Matrix-M1™ (M1) adjuvant

Principal Investigator: *[Investigator name]*
During office hours: [investigator contact number]
After hours and holidays: [investigator contact number]

CONCISE SUMMARY:
SARS-CoV-2, the virus that causes COVID-19, affects different people in different ways. Infected people can have no symptoms at all (known as asymptomatic infection) or have a wide range of symptoms – from mild to severe COVID-19 illness. A vaccine called "SARS-CoV-2 recombinant spike protein nanoparticle (SARS-CoV-2 rS) with Matrix-M1™ adjuvant" was shown to be safe in adults. Now this vaccine is being tested in the Pediatric PREVENT-19 trial to see whether it is safe and produces immune responses in children similar to those in adults.

We are inviting your child to take part in a swab study because he/she is enrolled in the Pediatric PREVENT-19 trial. The swab study will test whether the SARS-CoV-2 rS) with Matrix-M1™ vaccine also prevents children from getting asymptomatic SARS-CoV-2 infections that cause no symptoms but can still be passed to others. If you agree for your child to join the swab study, you will collect nasal swabs from your child (or supervise him/her collecting nasal swabs) at home twice a week for about 20 weeks by gently placing a nasal swab (a small stick with a soft tip) into the nose (both nostrils) and twirling it. The potential risk of taking part in this study is discomfort while obtaining the nasal swabs. There is also a potential risk of loss of confidentiality so we will label your child's study records and samples only with a study number and store them in a secure place. You, of course, may choose for your child not to participate or to withdraw from the swab study at any time. Even if you give consent, if your child does not want to participate, he/she will not be enrolled. Similarly, if your child wishes to enroll, but you choose not to allow him/her to participate, he/she will not be enrolled. Any child who turns 18 prior to the end of the study will be asked to provide consent either in person or remotely. If you are interested in learning more about this study, please continue to read below.

WHY IS THIS STUDY BEING PERFORMED?

Your child is being asked to take part in this research swab study because he/she is enrolled in "A 2-Part, Phase 3, Randomized, Observer-Blinded, Placebo-Controlled Study to Evaluate the Efficacy, Safety and Immunogenicity of a SARS-CoV-2 Recombinant Spike Protein Nanoparticle Vaccine (SARS-CoV-2 rS) with Matrix-M1™ Adjuvant in Adult Participants ≥ 18 Years, and the Safety and Effectiveness in Adolescents (12 to 17 Years) - PREVENT-19". The primary aim of the PREVENT-19 study is to find out whether the vaccine prevents COVID-19 illness and whether it is safe and effective in children. The PREVENT-19 study has two phases: a *primary injection phase* when your child will get two doses of either vaccine or placebo, and a *cross-over phase* several months later when participants who got placebo get two doses of vaccine, and those who got vaccine get two doses of placebo.

This swab study is an add-on study to the PREVENT-19 trial to learn whether SARS-CoV-2 rS/M1 vaccine prevents SARS-CoV-2 infection, including infections that cause no symptoms or very mild symptoms. When someone is infected with SARS-CoV-2, the virus can be found in his or her nose.

To see whether your child is infected with SARS-CoV-2, you will swab his/her nose (or supervise him/her swabbing the nose) twice a week from the day 21 clinic visit (or soon after that) until he/she receives vaccine or placebo as part of the *cross-over phase*. You will collect the swab by gently placing a nasal swab (a small stick with a soft tip) into your child's nose and twirling it, then placing the swab in a small tube. You will be instructed on how to swab your child's nose, how to package the swab for pick-up, and how to use a smart phone app to scan and track the swab shipments to our laboratory.

HOW MANY PEOPLE WILL TAKE PART IN THIS SWAB STUDY?

Up to 3,000 participants will be enrolled at up to 75 sites in the United States.

HOW LONG WILL YOU BE IN THIS STUDY?

Your participation in the study will last about 2-6 months.

WHAT IS INVOLVED IN THIS STUDY?

<u>Visit 1:</u>

We will explain the study to you and your child during your day 21 clinic visit or thereafter. The study staff will review the study requirements with you and your child to make sure that he/she can be in the study. The study staff will also go over the study plan and the informed consent document with you and your child. Then the following will occur. Overall, these steps will take about 20-30 minutes.

- Your child will be given a participant ID number.
- We will check your contact information (address, email, and phone number).
- We will help you or your child to download and register for the MG Scanner™ app.
- You will be taught how to collect a nasal swab from your child (or he/she will be taught to collect his/her own swab) and we will watch you or your child collect the first swab to make sure it is done correctly.
- You and your child will be given 40 swabs to keep at home to collect nasal swabs from your child twice a week.

Home swab collection

You will collect nasal swabs from your child two times a week (on Monday and Thursday), scan the vial barcode into your smart phone, and place the swabs in a plastic bag that we will give you. You will keep the swabs at room temperature – do not freeze or refrigerate them. We will also supply you with packaging so that you can send the swabs to our laboratory. You will receive reminders when it's time to collect the swab by a message in your email. If you miss collecting your swabs, we will call your cell phone. If you have any questions or problems with your swab collection, swab shipping, or the use of your App, please call us at (410)706-3890 between 7am and 7pm, Monday through Friday.

Final swab study visit

A few months into the study, the *cross-over phase* of the study will begin. People who got placebo will get 2 doses of vaccine 21 days apart and people who got vaccine will get 2 doses of placebo 21 days apart. You will collect the final swab from your child at the time of the cross-over phase. After the final swab is collected, your child's participation in the swab study will be completed.

Other data collection

We will look at your child's study records from the PREVENT-19 vaccine trial for information that will let us understand whether the vaccine prevents SARS-CoV-2 infection. The information that we look at will be linked by your child's participant ID and will not include personal identifiers.

Testing your swabs

Your child's swabs will be tested for the SARS-CoV-2 virus in the laboratory at Institute for Genome Sciences at the University of Maryland School of Medicine. The research will not include human whole genome sequencing, nor will your child's nasal swabs be used to generate a cell line for genetic testing.

Since this is a research study, you will not receive the results of your child's test. You and your child will not be contacted after the completion of the research. Your child's specimens will not be used for commercial profit.

WHAT ARE THE RISKS OF THE STUDY?

Nasal Swabs:

Collection of nasal swabs can cause sneezing, coughing, eye watering, mild discomfort and rarely nose bleeds. When these side effects occur, they last for a very short time.

Confidentiality:

Your/your child's contact information, including name, phone number, address, and email address, will be collected as part of the study. The information will be shared with the research team at the University of Maryland School of Medicine in Baltimore, Maryland, so they can make reminder calls and coordinate sample shipment. Additionally, there will be a link between your child's participant ID for this study and your child's participant ID for the PREVENT-19 study. There is a risk for loss of confidentiality. As described below, we will make every effort to protect your confidentiality.

WHAT ARE THE BENEFITS OF THE STUDY?

Your child will not benefit directly from your participation in this study. The results of this study will help us understand if SARS-CoV-2 rS/M1 vaccine prevents SARS-CoV-2 infection, including infections that have few or no symptoms.

WHAT IF YOU DO NOT WANT YOUR CHILD TO TAKE PART IN THE SWAB STUDY?

This is not a treatment study. Your alternative is for your child to not take part. If you choose for your child not to take part, this will not affect his/her participation in the PREVENT-19 study or the healthcare that your child receives.

ARE THERE COSTS TO YOU?

It will not cost you anything for your child to take part in this study. All items needed for the study will be provided.

IS THERE COMPENSATION FOR PARTICIPATING?

Your child will receive compensation for completing the study procedures. Your study team will give you a payment schedule and tell you how these payments will be done.

WILL YOUR CHILD'S INFORMATION BE KEPT CONFIDENTIAL?

All efforts, within reason, will be made to keep your child's personal information and research record confidential but total confidentiality cannot be guaranteed. Your child's information may be seen by members of the study team and others responsible for ensuring the safety and rights of participants and the proper conduct of the study. These organizations may include groups such as

the National Institute of Allergy and Infectious Diseases (NIAID) and it's contractors, Food and Drug Administration (FDA), study safety monitors, the Institutional Review Board and the Office for Research and Compliance, and the Centers for Disease Control and Prevention (CDC). We will share only the minimum necessary information in order to conduct this study. Your child's personal information may also be shared if required by law. Study investigators may share your child's information with other scientists without identifiers or use it for other research projects not listed in this form.

The informed consent documents and a face sheet containing your/your child's contact information will include your child's name and personal information. All other study records and samples will be labeled only with a study number and will not show your child's name. Your child's study records will be stored in locked cabinets and/or rooms in [*Site name*]. If captured electronically, personal data such as your child's name and contact information are password protected. Your child's samples will be stored in a sample bank at the University of Maryland School of Medicine. Only authorized personnel will have access to your child's stored data.

Records and samples labeled with your child's study identification number can be linked to your child's name. If you request, all links to your child's name or other information that personally identifies your child will be destroyed/removed from your child's samples after testing for the swab study is completed. Your child's specimens could be used for future research studies not described in this consent form without additional informed consent from you. If that occurs, information that could identify your child will be removed from the specimens. Your decision to remove the link to your child's identity will not involve any penalty or loss of access to treatment or other benefits to which your child is otherwise entitled.

We have a Certificate of Confidentiality from the US government to help protect your child's privacy. With the certificate, we do not have to release information about your child to someone who is not connected to the study, such as the courts or police. Sometimes we can't use the certificate. Since the US National Institutes of Health funds this research, we cannot withhold information from it.

Researchers may publish the results of this study or share the results at scientific meetings. Your child will not be identified in any published or shared information.

CAN YOU DECIDE FOR YOUR CHILD TO STOP PARTICIPATING IN THE STUDY?

Your child's participation in this study is voluntary. Your child does not have to take part in this research. You are free to withdraw your consent for your child to participate at any time. Refusal to take part or to stop taking part in the study will involve no penalty or loss of benefits to which your child is otherwise entitled.

If you decide to for your child to stop taking part, or if you have questions, concerns, or complaints, or if you need to report a medical injury related to the research, please contact the investigator, [*Investigator contact information]*.

Can YOUR CHILD be removed from the research?

The investigators in charge of the research study or the funding agencies (Centers for Disease Control and Prevention, NIH) can remove your child from the research study without your approval. Possible reasons for removal include failure to follow instructions of the research staff. The funding agency can also end the research study early. The study doctor will tell you about this and you will have the chance to ask questions if this were to happen. If you decide for your child to stop taking part in the Phase 3 trial, your child must also stop taking part in the swab study.

If your child suffers an injury directly related to your participation in this project, *[insert site name]* and/or one of its affiliated institutions or health care groups will help you obtain medical treatment for the specific injury and provide referrals to other health care facilities, as appropriate. *[Insert site name]* and/or its affiliated institutions or health care groups will not provide you with financial compensation or reimbursement for the cost of care provided to treat a research-related injury or for other expenses arising from a research-related injury. The institution or group providing medical treatment will charge your insurance carrier, you, or any other party responsible for your child's treatment costs. If you incur uninsured medical costs, they are your responsibility. No long-term medical care of financial compensation for research related injuries will be provided by the NIH of the Federal Gevernment. The study staff can give you more information about this if your child has a study injury.

By signing this Consent Form, you are not giving up any legal rights. If this research project is conducted in a negligent manner and your child is injured as a direct result, you may be able to recover the costs of care and other damages from the individuals or organizations responsible for your injury.

If you have any questions, concerns or complaints about the research or your child's rights as a participant, or any injury or if your child is unwell, please contact the study doctor at the phone number(s) listed above on the first page.

To protect your child's safety, rights, wellbeing and dignity, all research is reviewed by an independent group of people called an Institutional Review Board. You may talk to them at 855-818-2289 or researchquestions@wcgirb.com if:

- You have questions, concerns, or complaints that are not being answered by the research team.
- You are not getting answers from the research team.
- You cannot reach the research team.
- You want to talk to someone else about the research.
- You have questions about your child's rights as a research subject.

Signing this consent form indicates that you have read this consent form (or have had it read to you), that your questions have been answered to your satisfaction, and that you voluntarily agree for your child to participate in this research study. You will receive a copy of this signed consent form.

If you agree for your child to participate in this study, please sign your name below.

____
Child's Printed Name

____
Parent or Guardian's Printed Name

____
Parent or Guardian's Signature

Date:____________________________

____
Investigator or Designee Obtaining Consent Signature

Date:___________________________ Time:___________________

Witness: (to be completed only if the parent or legal guardian is illiterate and had the consent form read to him or her) I attest that the consent form was read to the parent/legal guardian and the parent/legal guardian had any questions answered before agreeing to allow his/her child to participate.

Printed name:_____________________________

Signature:_______________________________

Date:___________________________________

RESEARCH CONSENT FORM AND HIPAA AUTHORIZATION
(Parent or Guardian Permission Form)
V6.0; 20 AUGUST 2021

**TITLE:**	A nasal swab study to assess the efficacy of vaccination in the prevention of SARS-CoV-2 infection among individuals enrolled in a Phase 3 efficacy trial of a SARS-CoV-2 recombinant spike protein (rS) vaccine with Matrix-M1™ (M1) adjuvant
**PROTOCOL NO.:**	Coronavirus CVD 9000, 21-0011<br>IRB Protocol #20210587
**SPONSOR:**	National Institute of Allergy and Infectious Diseases (NIAID)
**INVESTIGATOR:**	Name<br>Address<br>City, State Zip<br>Country
**STUDY-RELATED PHONE NUMBER(S):**	Phone Number<br>Phone Number (24 hours)<br>[24 hour number is required]

CONCISE SUMMARY:
SARS-CoV-2, the virus that causes COVID-19, affects different people in different ways. Infected people can have no symptoms at all (known as asymptomatic infection) or have a wide range of symptoms – from mild to severe COVID-19 illness. A vaccine called "SARS-CoV-2 recombinant spike protein nanoparticle (SARS-CoV-2 rS) with Matrix-M1™ adjuvant" was shown to be safe in adults. Now this vaccine is being tested in the Pediatric PREVENT-19 trial to see whether it is safe and produces immune responses in children similar to those in adults.

We are inviting your child to take part in a swab study because he/she is enrolled in the Pediatric PREVENT-19 trial. The swab study will test whether the SARS-CoV-2 rS) with Matrix-M1™ vaccine also prevents children from getting asymptomatic SARS-CoV-2 infections that cause no symptoms but can still be passed to others. If you agree for your child to join the swab study, you will collect nasal swabs from your child (or supervise him/her collecting nasal swabs) at home twice a week for up to 28 weeks by gently placing a nasal swab (a small stick with a soft tip) into the nose (both nostrils) and twirling it. The potential risk of taking part in this study is discomfort while obtaining the nasal swabs. There is also a potential risk of loss of confidentiality so we will label your child's study records and samples only with a study number and store them in a secure place. You, of course, may choose for your child not to participate or to withdraw from the swab study at any time. Even if you give consent, if your child does not want to participate, he/she will not be enrolled. Similarly, if your child wishes to enroll, but you choose not to allow him/her to participate, he/she will not be enrolled. Any child who turns 18 prior to the end of the study will be asked to provide consent either in person or remotely. If you are interested in learning more about this study, please continue to read below.

WHY IS THIS STUDY BEING PERFORMED?
Your child is being asked to take part in this research swab study because he/she is enrolled in "A Phase 3, Randomized, Observer-Blinded, Placebo-Controlled Study to Evaluate the Efficacy, Safety, and Immunogenicity of a SARS-CoV-2 Recombinant Spike Protein Nanoparticle Vaccine (SARS-CoV-2 rS) with Matrix-M1™ Adjuvant in Adult Participants ≥ 18 Years with a Pediatric Expansion to Adolescents (12 to 17 Years)" also known as the PREVENT-19 study. The primary aim of the PREVENT-19 study is to find out whether the vaccine prevents COVID-19 illness and whether it is safe and effective in children. The PREVENT-19 study has two phases: a *primary injection phase* when your child will get two doses of either vaccine or placebo, and a *cross-over phase* several months later when participants who received placebo will get two doses of vaccine, and those who received vaccine will get two doses of placebo.

This swab study is an add-on study to the PREVENT-19 trial to learn whether SARS-CoV-2

rS/M1 vaccine prevents SARS-CoV-2 infection, including infections that cause no symptoms or very mild symptoms. When someone is infected with SARS-CoV-2, the virus can be found in his or her nose. Analyzing nasal swabs for SARS-CoV-2 is investigational.

To see whether your child is infected with SARS-CoV-2, you will swab his/her nose (or supervise him/her swabbing the nose) twice a week from the day 21 clinic visit (or soon after that) until December 17, 2021. You will collect the swab by gently placing a nasal swab (a small stick with a soft tip) into your child's nose and twirling it, then placing the swab in a small tube. You will be instructed on how to swab your child's nose, how to package the swab for pick-up, and how to use a smart phone app to scan and track the swab shipments to our laboratory at the University of Maryland School of Medicine.

The sample collection will continue after the cross-over phase. Extending swab collection, as the new school year begins, we hope to better understand how variants, like delta, might impact infection among people who are vaccinated.

HOW MANY PEOPLE WILL TAKE PART IN THIS SWAB STUDY?
Up to 3,000 participants will be enrolled at up to 75 sites in the United States.

HOW LONG WILL YOUR CHILD BE IN THIS STUDY?
Your child's participation in the study will last about 2-7 months.

WHAT IS INVOLVED IN THIS STUDY?
<u>Visit 1:</u>
We will explain the study to you and your child during your day 21 clinic visit of the PREVENT-19 study or as soon as possible thereafter. The study staff will review the study requirements with you and your child to make sure that he/she can be in the study. The study staff will also go over the study plan and the informed consent document with you and your child. Then the following will occur. Overall, these steps will take about 20-30 minutes.

- Your child will be given a participant ID number.
- We will check your contact information (address, email, and phone number).
- We will help you or your child to download and register for the MG Scanner™ app.
- You will be taught how to collect a nasal swab from your child (or he/she will be taught to collect his/her own swab) and we will watch you or your child collect the first swab to make sure it is done correctly.
- You and your child will be given 40 swabs to keep at home to collect nasal swabs from your child twice a week.

<u>Home swab collection</u>
You will collect nasal swabs from your child two times a week (preferably on Monday and Thursday), scan the vial barcode into your smart phone or Wi-Fi/camera enabled device, and

place the swabs in a plastic bag that we will give you. You will keep the swabs at room temperature – do not freeze or refrigerate them. We will also supply you with packaging so that you can send the swabs to our laboratory. You will receive reminders when it's time to collect the swab by a message in your email. If you miss collecting your swabs, we will call your cell phone. If you have any questions or problems with your swab collection, swab shipping, or the use of your App, please call us at (410)706-3890 between 10am and 6pm Eastern time, Monday through Friday.

If you do not have an appropriate smart phone or Wi-Fi/camera enabled device, or do not have an adequate number of devices to accommodate the number of participants in your household, the University of Maryland will provide one to you to use the MG Scanner mobile application to track your child's bi-weekly swabs. These devices do not have active cellular service or data plans. These devices will not be collected or returned at the end of the study.

Final swab study visit

A few months into the study, the *cross-over phase* of the PREVENT-19 study will occur. People who received placebo will get 2 doses of vaccine 21 days apart and people who received vaccine will get 2 doses of placebo 21 days apart. Once the cross-over is completed, your child will continue to collect twice weekly nasal swabs until December 17, 2021. After the final swab is collected, your child's participation in the swab study will be completed.

Other data collection

We will look at your child's study records from the PREVENT-19 vaccine trial for information that will let us understand whether the vaccine prevents SARS-CoV-2 infection. The information that we look at will be linked by your child's participant ID and will not include personal identifiers.

Testing your swabs

Your child's swabs will be tested for the SARS-CoV-2 virus in the laboratory at Institute for Genome Sciences at the University of Maryland School of Medicine. The research will not include human whole genome sequencing, nor will your child's nasal swabs be used to generate a cell line for genetic testing.

Since this is a research study, you will not receive the results of your child's test. You and your child will not be contacted after the completion of the research. Your child's specimens will not be used for commercial profit.

WHAT ARE THE RISKS OF THE STUDY?

Nasal Swabs:

Collection of nasal swabs can cause sneezing, coughing, eye watering, mild discomfort and rarely nose bleeds. When these side effects occur, they last for a very short time. There may be other unforeseeable risks.

Confidentiality:
Your/your child's contact information, including name, phone number, address, and email address, will be collected as part of the study. The information will be shared with the research team at the University of Maryland School of Medicine in Baltimore, Maryland, so they can make reminder calls and coordinate sample shipment. Additionally, there will be a link between your child's participant ID for this study and your child's participant ID for the PREVENT-19 study. There is a risk for loss of confidentiality. As described below, we will make every effort to protect your confidentiality.

WHAT ARE THE BENEFITS OF THE STUDY?

Your child will not benefit directly from your participation in this study. The results of this study will help us understand if SARS-CoV-2 rS/M1 vaccine prevents SARS-CoV-2 infection, including infections that have few or no symptoms.

WHAT IF YOU DO NOT WANT YOUR CHILD TO TAKE PART IN THE SWAB STUDY?

This is not a treatment study. Your alternative is for your child to not take part. If you choose for your child not to take part, this will not affect his/her participation in the PREVENT-19 study or the healthcare that your child receives.

ARE THERE COSTS TO YOU?

It will not cost you anything for your child to take part in this study. All items needed for the study will be provided.

IS THERE COMPENSATION FOR PARTICIPATING?

Your child will receive compensation for completing the study procedures. Your study team will give you a payment schedule and tell you how these payments will be done.

WILL YOUR CHILD'S INFORMATION BE KEPT CONFIDENTIAL?

All efforts, within reason, will be made to keep your child's personal information and research record confidential but total confidentiality cannot be guaranteed. Your child's information may be seen by members of the study team and others responsible for ensuring the safety and rights of participants and the proper conduct of the study. These organizations may include groups such as the National Institute of Allergy and Infectious Diseases (NIAID) and its contractors, Food and Drug Administration (FDA), study safety monitors, the Institutional Review Board and the Office for Research and Compliance, and the Centers for Disease Control and Prevention (CDC). We will share only the minimum necessary information in order to conduct this study. Your child's personal information may also be shared if required by law. Study investigators may share your child's information with other scientists without identifiers or use it for other research projects not listed in this form.

The informed consent documents and a face sheet containing your/your child's contact

information will include your child's name and personal information. Additionally, the address labels with which your child's samples are shipped will include your child's personal information. All other study records and samples will be labeled only with a study number and will not show your child's name. Your child's study records will be stored in locked cabinets and/or rooms in [Site name]. If captured electronically, personal data such as your child's name and contact information are password protected. Your child's samples will be stored in a sample bank at the University of Maryland School of Medicine. Only authorized personnel will have access to your child's stored data.

Records and samples labeled with your child's study identification number can be linked to your child's name. If you request, all links to your child's name or other information that personally identifies your child will be destroyed/removed from your child's samples after testing for the swab study is completed. Your child's specimens could be used for future research studies not described in this consent form without additional informed consent from you. If that occurs, information that could identify your child will be removed from the specimens. Your decision to remove the link to your child's identity will not involve any penalty or loss of access to treatment or other benefits to which your child is otherwise entitled.

We have a Certificate of Confidentiality from the US government to help protect your child's privacy. With the certificate, we do not have to release information about your child to someone who is not connected to the study, such as the courts or police. Sometimes we can't use the certificate. Since the US National Institutes of Health funds this research, we cannot withhold information from it.

Researchers may publish the results of this study or share the results at scientific meetings. Your child will not be identified in any published or shared information.

CAN YOU DECIDE FOR YOUR CHILD TO STOP PARTICIPATING IN THE STUDY?

Your child's participation in this study is voluntary. Your child does not have to take part in this research. You are free to withdraw your consent for your child to participate at any time. Refusal to take part or to stop taking part in the study will involve no penalty or loss of benefits to which your child is otherwise entitled.

If you decide to for your child to stop taking part, or if you have questions, concerns, or complaints, or if you need to report a medical injury related to the research, please contact the investigator, [Investigator contact information], at the number on the first page.

Can YOUR CHILD be removed from the research?

The investigators in charge of the research study or the funding agencies (Centers for Disease Control and Prevention, NIH) can remove your child from the research study without your approval. Possible reasons for removal include failure to follow instructions of the research staff. The funding agency can also end the research study early. The study doctor will tell you about

this and you will have the chance to ask questions if this were to happen. If you decide for your child to stop taking part in the Phase 3 trial, your child must also stop taking part in the swab study.

If your child suffers an injury directly related to your participation in this project, [insert site name] and/or one of its affiliated institutions or health care groups will help you obtain medical treatment for the specific injury and provide referrals to other health care facilities, as appropriate. [Insert site name] and/or its affiliated institutions or health care groups has no plans to provide you with financial compensation or reimbursement for the cost of care provided to treat a research-related injury or for other expenses arising from a research-related injury. The institution or group providing medical treatment will charge your insurance carrier, you, or any other party responsible for your child's treatment costs. If you incur uninsured medical costs, they may be your responsibility. There are no plans for long-term medical care or financial compensation for research related injuries to be provided by the NIH of the Federal Government. The study staff can give you more information about this if your child has a study injury.

By signing this Consent Form, you are not giving up any legal rights to pursue a claim through the legal system. If this research project is conducted in a negligent manner and your child is injured as a direct result, you may be able to recover the costs of care and other damages from the individuals or organizations responsible for your injury.

The US government will pay the study doctor and/or the study site to cover their costs of conducting this study.

If you have any questions, concerns or complaints about the research or your child's rights as a participant, or any injury or if your child is unwell, please contact the study doctor at the phone number(s) listed above on the first page.

To protect your child's safety, rights, wellbeing and dignity, all research is reviewed by an independent group of people called an Institutional Review Board. You may talk to them at 855-818-2289 or researchquestions@wcgirb.com if:

- You have questions, concerns, or complaints that are not being answered by the research team.
- You are not getting answers from the research team.
- You cannot reach the research team.
- You want to talk to someone else about the research.
- You have questions about your child's rights as a research subject.

Signing this consent form indicates that you have read this consent form, that your questions have been answered to your satisfaction, and that you voluntarily agree for your child to participate in this research study. You will receive a copy of this signed and dated consent form.

If you agree for your child to participate in this study, please sign your name below.

____
Child's Printed Name

____
Parent or Guardian's Printed Name

____
Parent or Guardian's Signature

Date:______________________________

____
Signature of adult subjects capable of consent (previously provided assent)

____
Printed name of adult subjects capable of consent (previously provided assent)

Date:______________________________

All children are required to assent, unless the investigator determines that the capability of the child is so limited that the child cannot reasonably be consulted.

If assent is obtained, have the child sign an assent form, unless the investigator determines that the child is not capable of signing

____
Printed Name of Investigator or Designee Obtaining Consent

____
Investigator or Designee Obtaining Consent Signature

Date:___________________________

Time:__________________

****For Sites in California****

AUTHORIZATION TO USE AND DISCLOSE INFORMATION FOR RESEARCH PURPOSES

What information may be used and given to others?

The study doctor will get your child's personal and medical information. For example:

- Past and present medical records
- Research records
- Records about phone calls made as part of this research
- Records about your child's study visits.

Who may use and give out information about your child?

The study doctor and the study staff. [They may also share the research information with [enter SMO company name], an agent for the study doctor. delete if the site does not have an SMO]

Who might get this information?

The sponsor of this research. "Sponsor" means any persons or companies that are:

- working for or with the sponsor, or
- owned by the sponsor.

Your child's information <u>may</u> be given to:

- The U.S. Food and Drug Administration (FDA),
- Department of Health and Human Services (DHHS) agencies,
- Governmental agencies in other countries,
- The institution where the research is being done,
- Governmental agencies to whom certain diseases (reportable diseases) must be reported, and
- Institutional Review Board (IRB)

Why will this information be used and/or given to others?

- to do the research,
- to study the results, and

- to make sure that the research was done right.

If the results of this study are made public, information that identifies your child will not be used.

What if I decide not to give permission to use and give out my child's health information?
Then your child will not be able to be in this research study.

May I review or copy my child's information?
Yes, but only after the research is over.

May I withdraw or revoke (cancel) my permission?
This permission will be good until December 31, 2070.

You may withdraw or take away your permission to use and disclose your child's health information at any time. You do this by sending written notice to the study doctor. If you withdraw your permission, your child will not be able to stay in this study.

When you withdraw your permission, no new health information identifying your child will be gathered after that date. Information that has already been gathered may still be used and given to others.

Is my child's health information protected after it has been given to others?
There is a risk that your child's information will be given to others without your permission.

Authorization:
I have been given the information about the use and disclosure of my child's health information for this research study. My questions have been answered.

I authorize the use and disclosure of my child's health information to the parties listed in the authorization section of this consent for the purposes described above.

AUTHORIZATION SIGNATURE:

____ _____________

Signature of Parent/Guardian **Date**

Protocol 21-0011 Summary of changes: Informed consent (Parental)
Version 2.0 (22 APR 2021) to Version 6.0 (20 Aug 2021)

**Location of Change**	**Change/modification to version 6.0 (Final version)**
Page 1	Header formatting updated
Page 3	Clarified that analyzing nasal swabs for SARS-CoV-2 is investigational.<br>Clarified that the laboratory that will be testing the swabs is at the University of Maryland School of Medicine.<br>Extended duration of the study beyond the cross-over phase, and to up to 7 months.
Page 7	Restated that the US government funds the study/sites.

IRB Approved at the
Study Level
May 11, 2021

#29937551.1

Nasal Swab Self-Collection for COVID-19 Testing

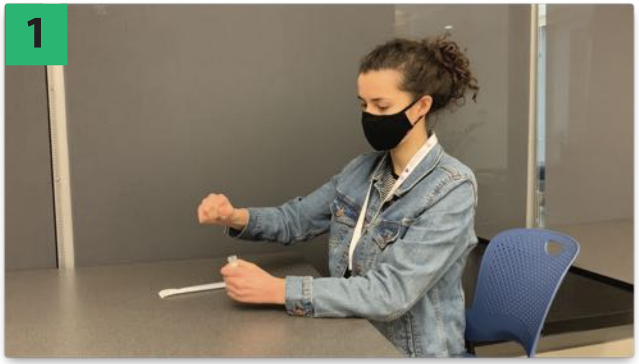

Carefully open the snapcap of the sample collection tube and place it on a clean flat surface.

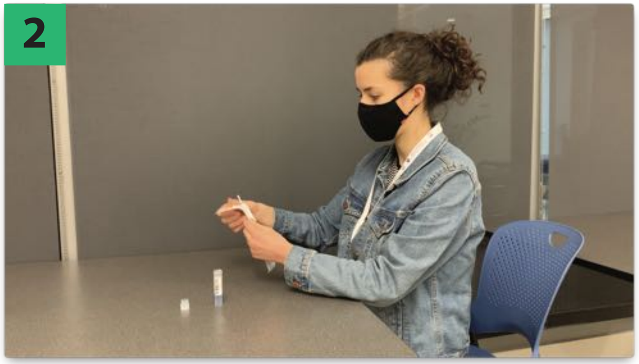

Open the nasal swab pouch and remove the swab.
Tip: Before sampling, gently blow you nose in a tissue.

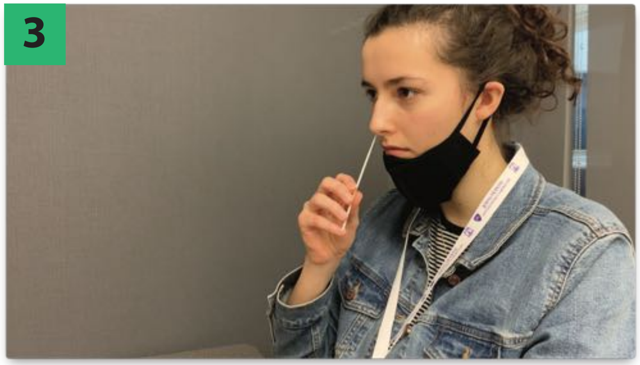

Insert the swab at least 1 inch into one nostril and rotate against the inside wall of the nose at least 2-3 times. It should feel uncomfortable but not painful.

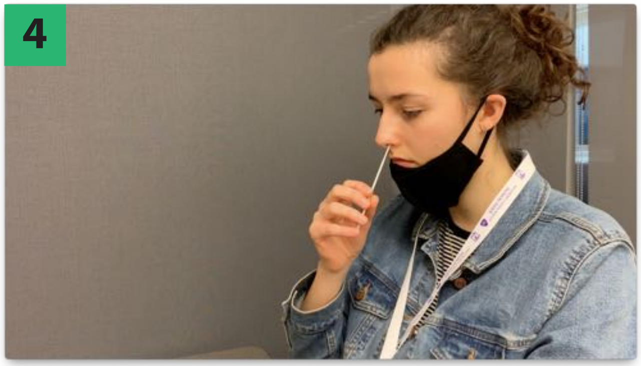

Using the same swab repeat with your other nostril.

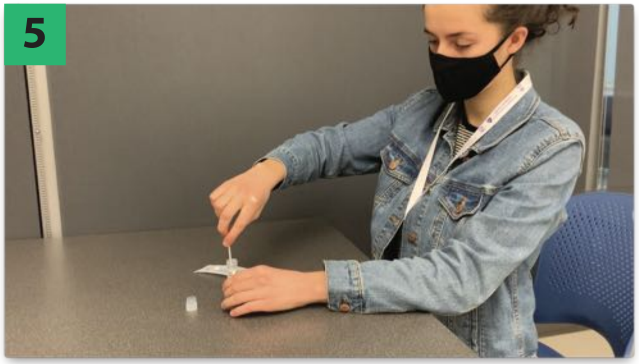

Place the swab into the tube and swirl the swab in the liquid for 15-30 seconds. Leave the tip of the swab in the tube. Snap off the end of the swab by bending the plastic handle against the edge of the tube.

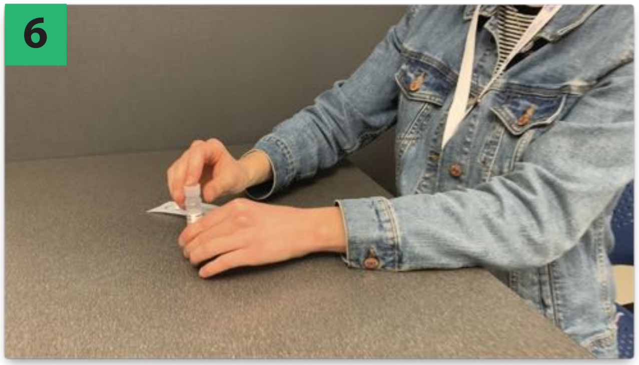

Snap the cap securely back on the collection tube.

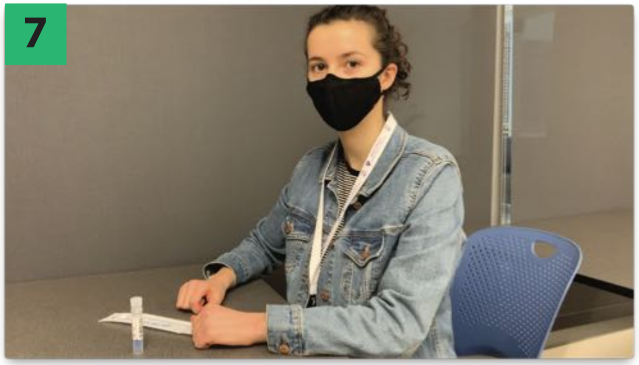

All done!

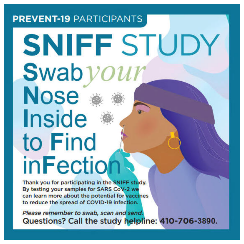

This kit is used to collect your swabs every Monday and Thursday for the SNIFF study

PREVENT-19 PARTICIPANTS

SNIFF STUDY

Swab *your* **N**ose **I**nside to **F**ind in**F**ection

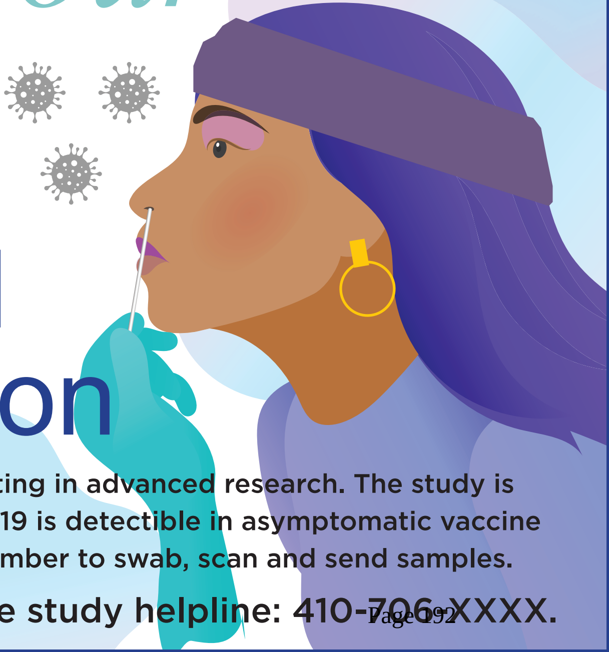

Thank you for participating in advanced research. The study is testing whether COVID-19 is detectible in asymptomatic vaccine volunteers. Please remember to swab, scan and send samples.

Questions? Call the study helpline: 410-706-XXXX.

PREVENT-19 PARTICIPANTS

SNIFF STUDY

Swab *your* **N**ose **I**nside to **F**ind in**F**ection

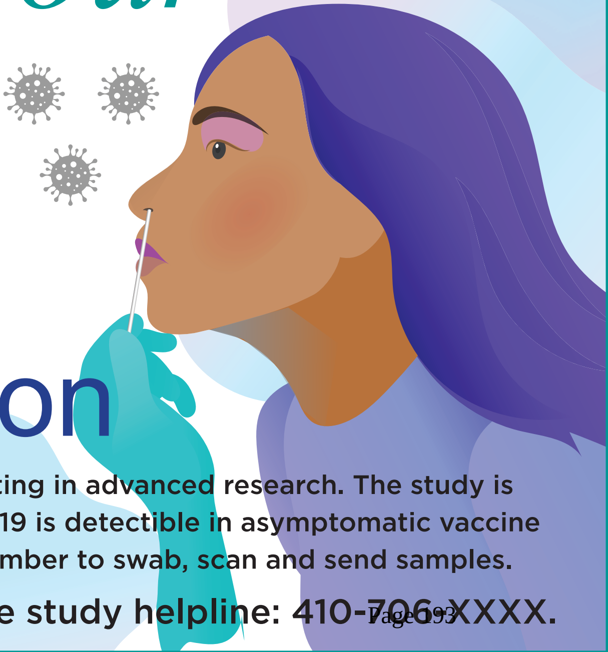

Thank you for participating in advanced research. The study is testing whether COVID-19 is detectible in asymptomatic vaccine volunteers. Please remember to swab, scan and send samples.

Questions? Call the study helpline: 410-706-XXXX.

SNIFF STUDY

Swab *your* Nose Inside to Find inFection

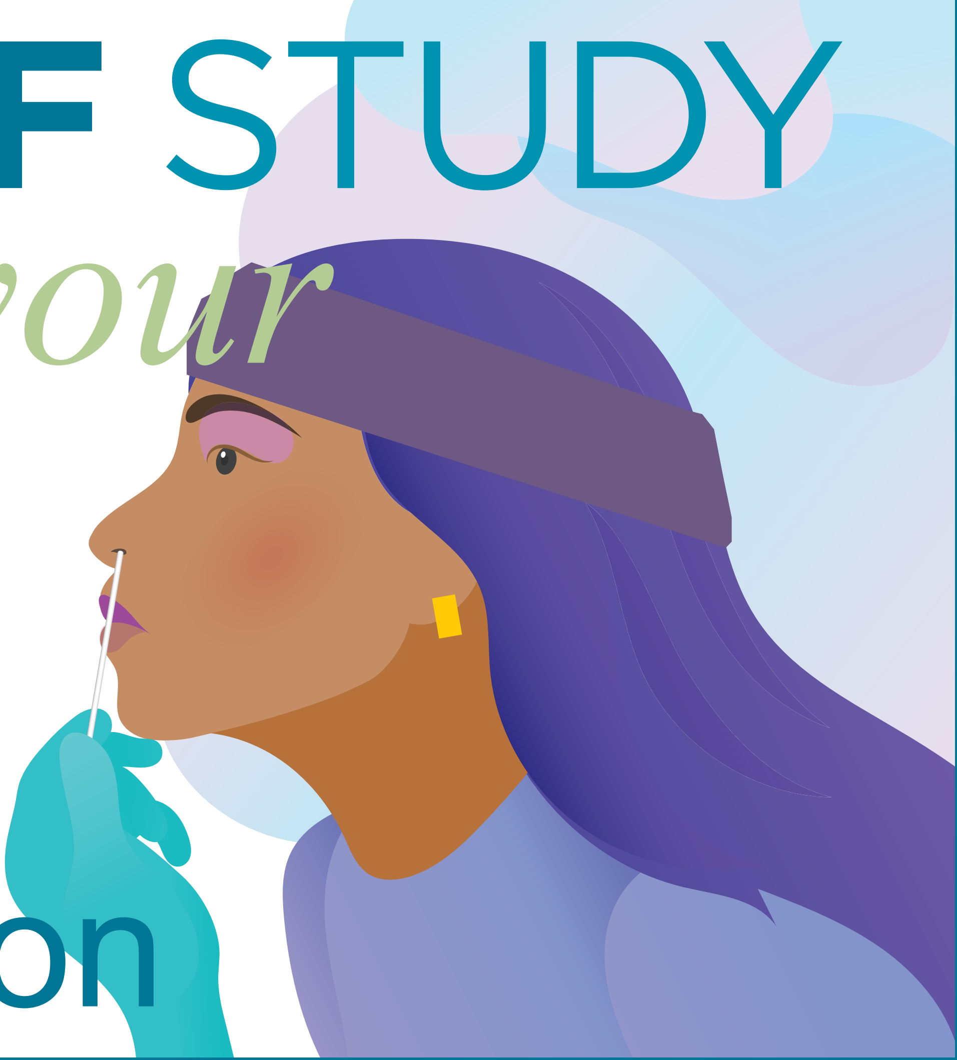

Thank you for participating in the SNIFF study. We'd like to invite you to continue your participation in the study.

By testing your samples for SARS CoV-2 we can learn more about the potential for vaccines to reduce the spread of COVID-19 infection.

As the new school year begins, we plan to extend the twice-weekly swab collection to better understand how variants, like Delta, might impact infection among people who are vaccinated, and how vaccination affects transmission to those around them.

You will have the opportunity to swab, scan, send in samples through December 17th 2021. Please talk to your study team to get more information. You must sign a new consent form to participate in this extension.

We look forward to your continued participation in this very important study!

ESTUDIO SNIFF

Si en tu Nariz hay Infección, descúbrelo Frotando Fácilmente

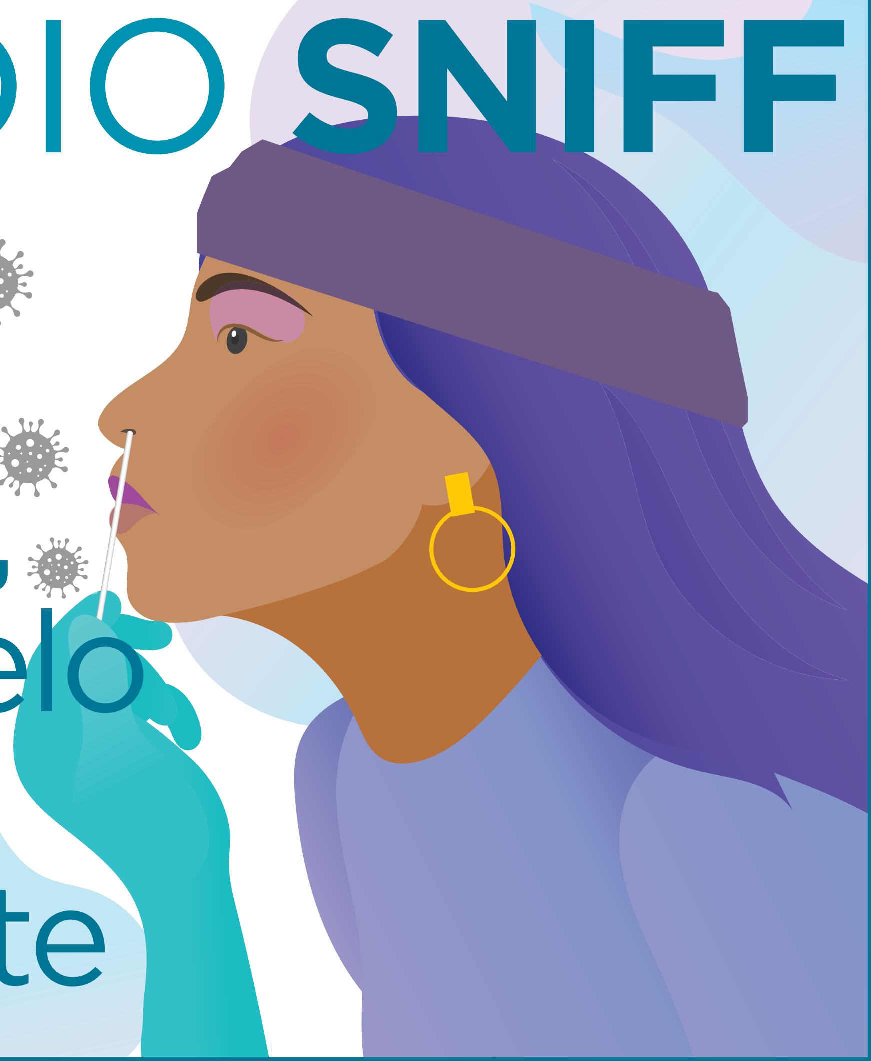

¡Hola, participante de SNIFF!

Gracias por participar en el estudio SNIFF. Nos ha ayudado a estudiar una vacuna COVID y si puede prevenir incluso infecciones muy leves. Su contribución a estos estudios de investigación puede ayudarnos a detener la propagación de COVID en nuestras comunidades, en nuestro país y en todo el mundo. ¡Estamos muy agradecidos por su participación en este estudio de investigación! Sin la generosidad de personas como usted que se ofrecen como voluntarios para la investigación de vacunas, nunca hubiéramos encontrado vacunas COVID efectivas. ¡Deténgase un minuto para pensar en eso!

Este es un recordatorio de que el hisopo final para el estudio SNIFF debe recolectarse a más tardar el viernes 17 de diciembre de 2021. Recuerde escanear la muestra el mismo día de la recolección y enviar sus muestras al laboratorio. Si tiene suministros sin usar después del día 17, puede desecharlos. Si le proporcionamos un teléfono móvilpara escanear los hisopos, no es necesario que lo devuelva.

Si tiene alguna inquietud, comuníquese con su sitio local o con la línea de ayuda del estudio al 410-706-3890.

¡Gracias nuevamente por su participación en este importante estudio!

El equipo SNIFF

SNIFF STUDY

Swab *your* Nose Inside to Find inFection

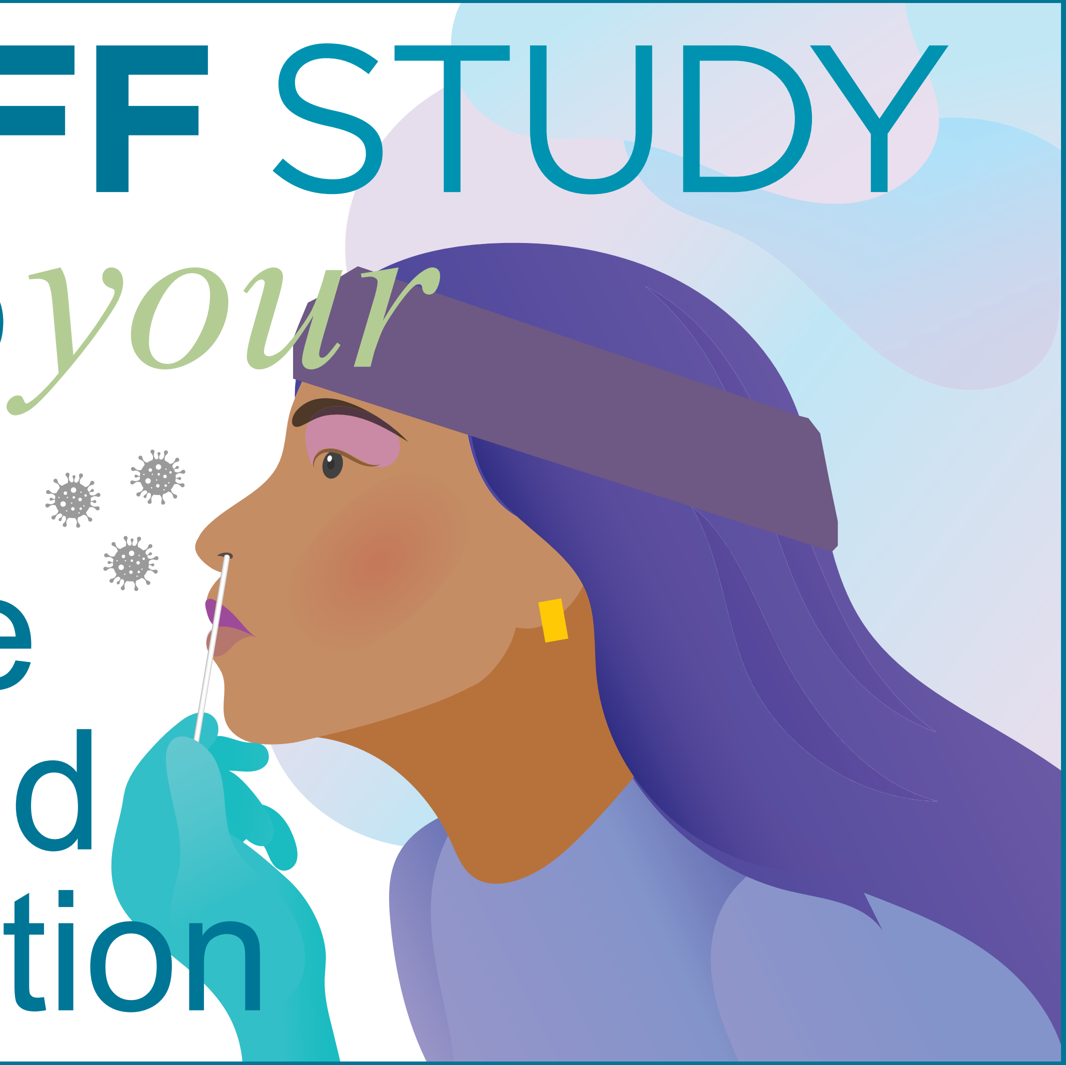

Hello SNIFF participant!

Thank you for participating in the SNIFF study. You have helped us study a COVID vaccine and whether it can prevent even very mild infections. Your contribution to these research studies may help us to stop COVID from spreading in our communities, in our country, and around the world. We are very thankful for your participation in this research study! Without the generosity of people like you who volunteer for vaccine research, we would never have found effective COVID vaccines – stop for a minute to think about that!

This is a reminder that the final swab for the SNIFF study should be collected no later than Friday December 17, 2021. Please remember to scan the sample on the same day as collection and ship your samples back to the lab.

If you have any unused supplies after the 17th they can be discarded. If we gave you a device for scanning the swabs it does not need to be returned.

If you have any concerns, please reach out to your local site, or the study helpline at 410-706-3890.

Thank you again for your participation in this important study!

The SNIFF Team

SNIFF STUDY

Swab *your* **N**ose **I**nside to **F**ind in**F**ection

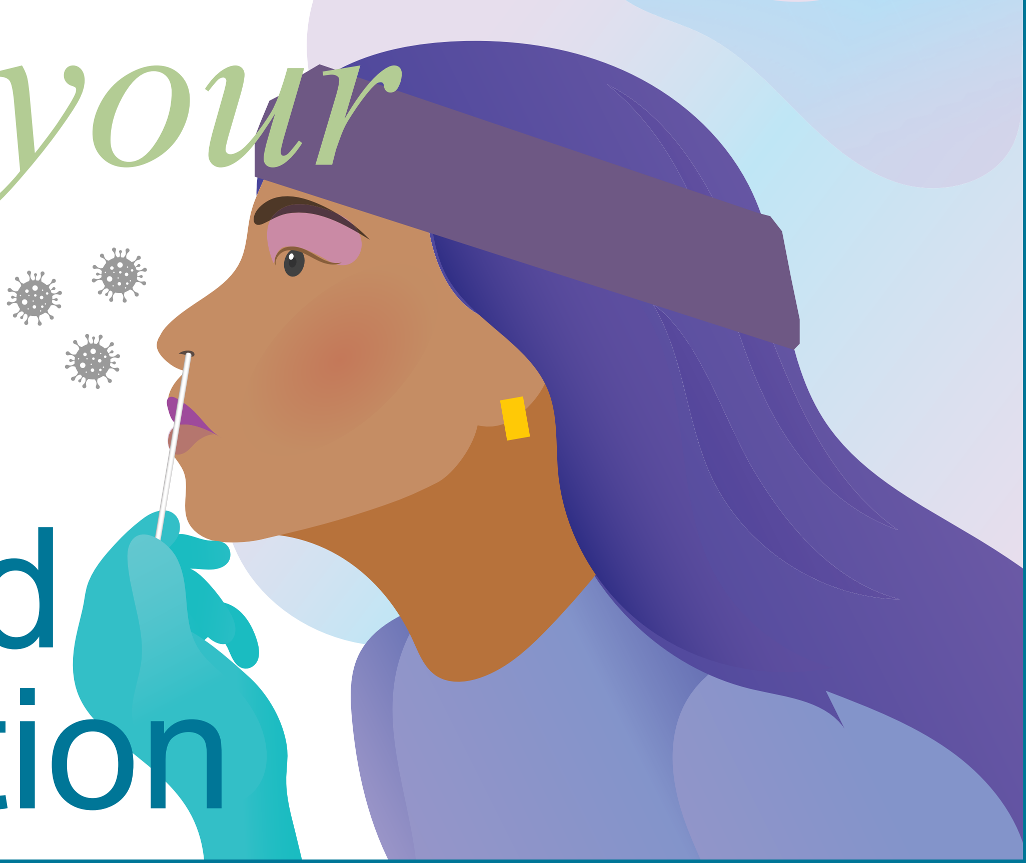

¡Hola, participante de SNIFF!

Gracias por participar en el estudio SNIFF. Nos ha ayudado a estudiar una vacuna COVID y si puede prevenir incluso infecciones muy leves. Su contribución a estos estudios de investigación puede ayudarnos a detener la propagación de COVID en nuestras comunidades, en nuestro país y en todo el mundo. ¡Estamos muy agradecidos por su participación en este estudio de investigación! Sin la generosidad de personas como usted que se ofrecen como voluntarios para la investigación de vacunas, nunca hubiéramos encontrado vacunas COVID efectivas. ¡Deténgase un minuto para pensar en eso!

Este es un recordatorio de que el hisopo final para el estudio SNIFF debe recolectarse a más tardar el viernes 17 de diciembre de 2021. Recuerde escanear la muestra el mismo día de la recolección y enviar sus muestras al laboratorio. Si tiene suministros sin usar después del día 17, puede desecharlos. Si le proporcionamos un teléfono móvilpara escanear los hisopos, no es necesario que lo devuelva.

Si tiene alguna inquietud, comuníquese con su sitio local o con la línea de ayuda del estudio al 410-706-3890.

¡Gracias nuevamente por su participación en este importante estudio!

El equipo SNIFF
